# Potential therapeutic effects of Chinese meteria medica in mitigating drug-induced acute kidney injury

**DOI:** 10.3389/fphar.2023.1153297

**Published:** 2023-04-03

**Authors:** Jun Li, Tonglu Li, Zongping Li, Zhiyong Song, Xuezhong Gong

**Affiliations:** Department of Nephrology, Shanghai Municipal Hospital of Traditional Chinese Medicine, Shanghai University of Traditional Chinese Medicine, Shanghai, China

**Keywords:** drug-induced acute kidney injury, Chinese meteria medica, metabolites, molecular mechanisms, review

## Abstract

Drug-induced acute kidney injury (DI-AKI) is one of the leading causes of kidney injury, is associated with high mortality and morbidity, and limits the clinical use of certain therapeutic or diagnostic agents, such as antineoplastic drugs, antibiotics, immunosuppressants, non-steroidal anti-inflammatory drugs, and contrast media. In recent years, numerous studies have shown that many Chinese meteria medica, metabolites derived from botanical drugs, and Chinese medicinal formulas confer protective effects against DI-AKI by targeting a variety of cellular or molecular mechanisms, such as oxidative stress, inflammatory, cell necrosis, apoptosis, and autophagy. This review summarizes the research status of common DI-AKI with Chinese meteria medica interventions, including cisplatin, gentamicin, contrast agents, methotrexate, and acetaminophen. At the same time, this review introduces the metabolites with application prospects represented by ginseng saponins, tetramethylpyrazine, panax notoginseng saponins, and curcumin. Overall, this review provides a reference for the development of promising nephroprotectants.

## 1 Introduction

Acute kidney injury (AKI) is characterized by the sudden loss of renal function and is a common condition in hospitalized patients (Khwaja, 2012). The total incidence and mortality of AKI in adults is 21.6% and 23.9%, respectively, and the associated medical and economic burden is increasing (Lewington et al., 2013; Susantitaphong et al., 2013). In addition to higher mortality, AKI is also associated with the development of chronic kidney disease (Chawla et al., 2014). Drug-induced AKI (DI-AKI) is an important branch of AKI that is caused primarily by kidney exposure to toxic or potentially toxic drugs, typically antimicrobials, chemotherapy drugs, analgesics, and immunosuppressants (Perazella, 2018). The epidemiology of DI-AKI is largely based on the AKI literature, and available data indicate that it accounts for approximately 20% of AKI cases in hospitalized patients (Mehta et al., 2015). However, most of the prevention and treatment strategies for DI-AKI are still concentrated in the preclinical research stage, so there is an urgent need to explore therapies for DI-AKI (Perazella and Rosner, 2022). For centuries, botanical drugs and Chinese medicinal formulas derived from traditional Chinese medicine (TCM) theory and practice have been used to treat many ailments in China. At present, Chinese medicine has received strong support from the World Health Organization and is included in Chapter 26 of the 11th edition of the Global Medical Program. The renoprotective effects of some Chinese medicinal formulas and metabolites have received widespread attention and have been studied in multiple AKI models, including DI-AKI (Wang et al., 2019; Li and Gong, 2022; Rui et al., 2022; Zou et al., 2022). In this review, whether Chinese meteria medica and their preparations or monomers can improve DI-AKI and their potential mechanisms of action were explored.

## 2 Research status of DI-AKI

As kidneys and liver are the main organs involved in drug metabolism, many drugs pose a potential risk of causing DI-AKI. Available data suggest that drugs such as antibiotics, antifungals, antivirals, chemotherapeutic agents, analgesics, radiographic contrast agents, calcium phosphatase inhibitors, bisphosphonates, proton pump inhibitors, anticonvulsants, and diuretics cause DI-AKI (Perazella and Rosner, 2022). Antibiotics and antineoplastic drugs are the most frequently reported nephrotoxic drugs (Izzedine and Perazella, 2017; Rey et al., 2022; Williams et al., 2022). Based on the KDIGO guideline classification, 50.2% of DI-AKI cases were in AKI stage 1% and 49.8% were in stages 2 or 3 (Rolland et al., 2021). In addition, community- and hospital-acquired DI-AKI differ in several ways. A retrospective study in France showed that antibiotics, diuretics, and contrast agents were significantly more associated with cases of hospital-acquired DI-AKI, while antineoplastics, lipid-lowering drugs, hypoglycemic agents, and immunosuppressive agents were mostly associated with cases of community-acquired DI-AKI (Rey et al., 2022). Besides the common drugs mentioned, the nephrotoxicity of some botanical drugs should also be considered, though all of them do not drive DI-AKI (Su et al., 2011; Yang et al., 2018). According to available studies, botanical drugs with potential nephrotoxicity mainly include the following categories: a) *Isotrema manshuriense* (Kom.) H. Huber (aristolochic acid) and *Asarum heterotropoides* F. Schmidt, which contain organic acids; b) *Aconitum carmichaelii* Debeaux (Aconitine), *Strychnos nux-vomica* L. (Brucine), *Leonurus japonicus* Houtt. (Leonurine), *etc.*, which all contain alkaloids; c) Flavonoid-containing herbs such as *Scutellaria barbata* D. Don; d) Anthraquinone-containing herbs such as *Rheum officinale* Baill. (Emodin, Aloeemodin); e) *Gardenia jasminoides* J. Ellis (Geniposide), *Phytolacca acinosa* Roxb. (Esculentoside), *etc.*, which all contain glycosides; f) *Tripterygium wilfordii* Hook. f. (Triptolide) and *Andrographis paniculata* (Burm.f.) Nees (Andrographolide), *etc.*, which contain terpenoids and lactones; g) *Xanthium strumarium* L., and *Scolopendra subspinipes mutilans* L. Koch., *etc.*, Containing toxic proteins; h) Other toxic components include *Mylabris phalerata* Pallas (Cantharidin), *Cnidium monnieri* (L.) Cusson (Osthole), etc., (Rao et al., 2022; Tian et al., 2022; Zhang et al., 2023). Incidences of DI-AKI caused by botanical drugs are unknown, which should be considered by clinicians when acquiring patient history. Some drugs may also cause pseudo-AKI by blocking tubular creatinine secretion or affecting renal hemodynamics, which are generally not included in the category of DI-AKI. In addition to the drug characteristics, underlying patient characteristics, such as the presence of chronic kidney diseases or anemia, old age, and female sex, could increase their susceptibility to the drug effects (Robert et al., 2019; Liu et al., 2021). To a large extent, the treatments of DI-AKI and AKI are similar, focusing on discontinuation of the causative drug. Several studies have focused on risk prediction models, molecular probes, and sensitive biomarkers to predict early DI-AKI and prevent the use of potentially nephrotoxic drugs (Weng et al., 2021; Yousif and Awdishu, 2023). Given that effective therapies for DI-AKI are still lacking, the development of promising treatments is imperative.

## 3 Methodology

According to the Pharmacopoeia of the People’s Republic of China revised by the State Food and Drug Administration in 2021, Chinese meteria medica refers to treatments based on plant herbal products, botanical drugs, and materials of mineral and animal origin. This review aimed to include *in vivo* and *in vitro* experimental studies of interventions for DI-AKI, including Chinese meteria medica and their extracts, metabolites, and Chinese medicinal formulas. Literature published prior to 1 December 2021, on DI-AKI treated with Chinese meteria medica was reviewed (from the PubMed and Embase databases). The following combinations of terms were used as search keywords: “herbal medicine”, “phytochemical”, “botanical drugs”, “drug-induced nephropathy”, and “acute kidney injury”. Specified exclusion criteria included a) randomized controlled trials, case reports, case series, editorials, reviews, *etc.*,; b) interventions containing ingredients other than Chinese meteria medica; c) the model involved in the study not being DI-AKI; d) the full text not being available; and e) articles not written in English.

## 4 Results

**FIGURE 1:**
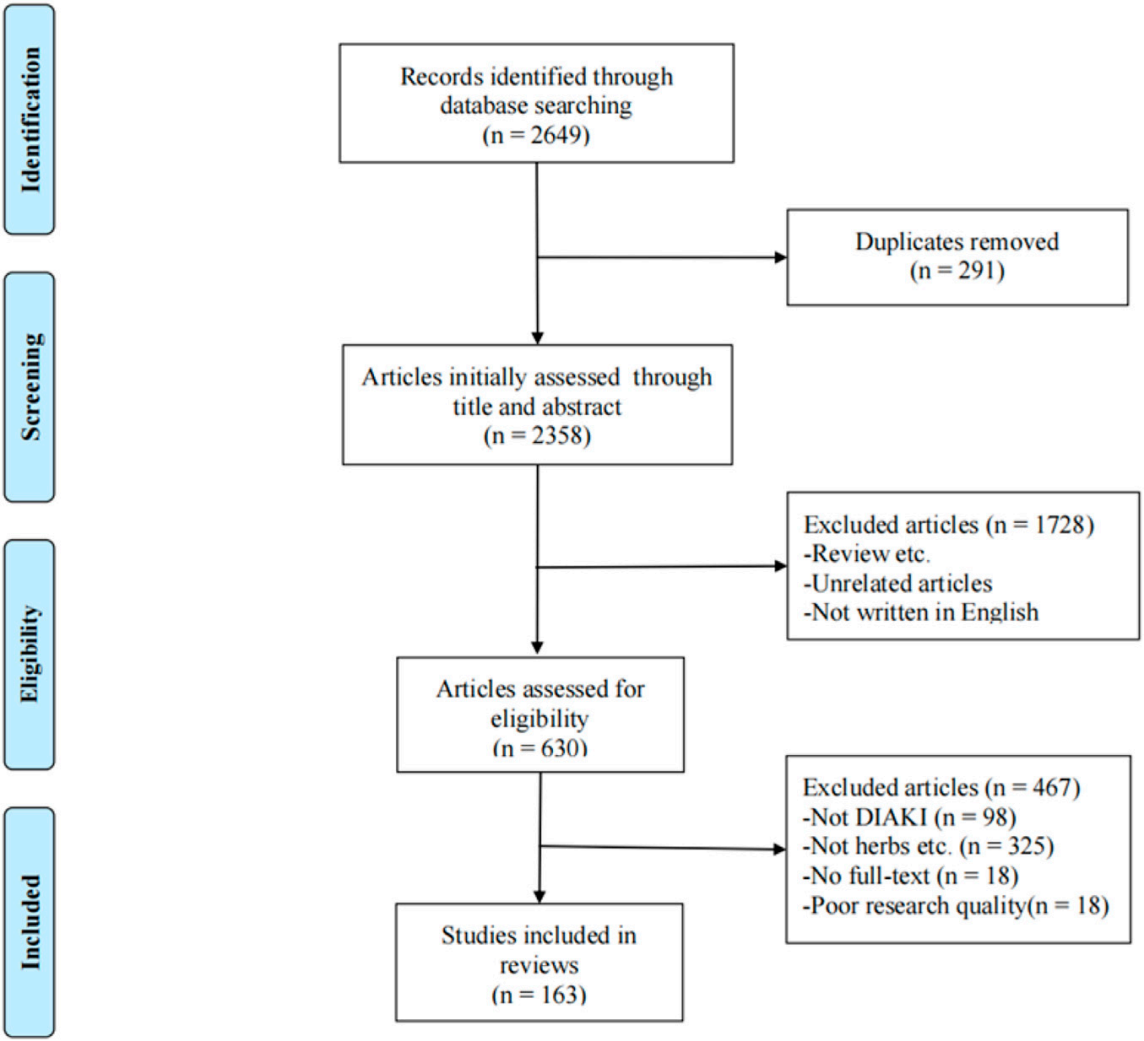
Summary of the literature search process.

The flow chart (Figure 1) of article processing shows that the search yielded 2,649 articles, and 2,486 articles were excluded based on the exclusion criteria. After excluding these 2,486 articles, 163 articles were included, consisting of 113 *in vivo* studies, 17 *in vitro* studies, and 33 studies that combined both *in vitro* and *in vivo* experiments. Based on further reading of these studies, 140 *in vivo* and *in vitro* studies were divided into five groups based on the drug used: chemotherapy-related DI-AKI, antimicrobial-related DI-AKI, NSAID-related DI-AKI, contrast media-related DI-AKI, and other drug-related DI-AKI (Supplementary Tables S1–S5). The compositions of the Chinese medicinal formulas included in the studies are provided in (Supplementary Table S6).

## 5 Pathological mechanisms of DI-AKI

**FIGURE 2:**
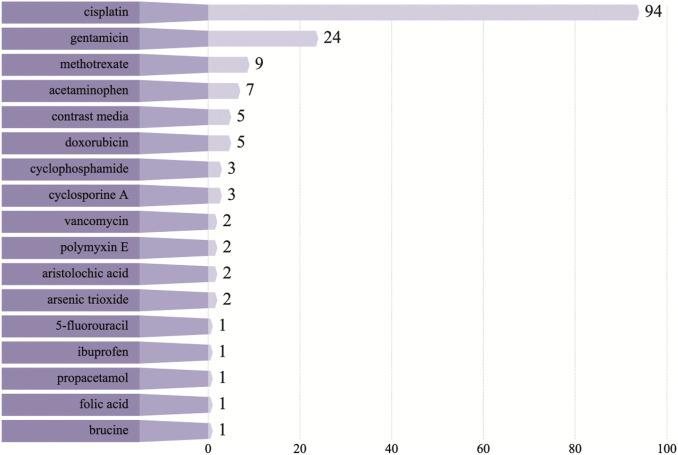
Frequency distribution of nephrotoxic drugs in the included studies. Drug-induced acute kidney injury caused by cisplatin, gentamicin, methotrexate, acetaminophen, and contrast media is the focus of Chinese meteria medica research.

Seventeen nephrotoxic drugs: gentamicin, methotrexate (MTX), acetaminophen, contrast media, cyclophosphamide, cyclosporine A, doxorubicin, vancomycin, carboplatin, 5-fluorouracil, polymyxin E, ibuprofen, propacetamol, aristolochic acid, folic acid, and brucine, were included in the studies considered for this review. It is not difficult to speculate that studies related to cisplatin, gentamicin, MTX, acetaminophen, and contrast media are hot spots for Chinese meteria medica interventions in DI-AKI (Figure 2). Although many nephrotoxic drugs have been described, the mechanisms by which they cause kidney damage mainly include the following three types. The first is the direct toxic effect of the drug on renal tubules, which can induce cell damage and death, leading to acute tubular necrosis (ATN). The most important phenotypes of ATN in DI-AKI and common nephrotoxic agents associated with ATN include cisplatin, gentamicin, contrast agents, acetaminophen, and cyclosporine (Mehta et al., 2015). The pathological mechanisms of ATN mainly involve oxidative stress and direct cytotoxic effects associated with mitochondrial damage (Hosohata, 2016; Gai et al., 2020). Acute interstitial nephritis (AIN) is another immune-mediated DI-AKI phenotype characterized by the infiltration of immune cells into the tubular interstitium. Studies have reported about 15% observable AIN in biopsies of AKI (Perazella and Markowitz, 2010; Muriithi et al., 2013). Many drugs can cause AIN, including antibiotics (e.g., β-lactams, fluoroquinolones, and sulfonamides), non-steroidal anti-inflammatory drugs (NSAIDs), proton pump inhibitors (PPI), and immune checkpoint inhibitors (Muriithi et al., 2014). These drugs can bind to the tubular basement membrane and act as hapsumins or can be deposited in the tubular interstitium and manifest as implant antigens. Dendritic cells scattered between tubular cells recognize these drug-associated antigens and migrate to local lymph nodes, driving the immune processes associated with T cells and leading to AIN (Spanou et al., 2006; Caravaca-Fontán et al., 2019). The third important route of injury in DI-AKI is the insolubility of a drug in urine, which causes it to precipitate into crystals in the renal tubules and is accompanied by an inflammatory response, also known as crystalline nephropathy (CN). Crystalline deposition of a drug in the kidneys is mainly due to an increase in the supersaturation of a drug in the urine and may be associated with an overdose, unsuitable urine pH, and underlying kidney disease, and common drugs that induce CN include MTX, sulfadiazine, and ciprofloxacin (Daudon et al., 2018; Perazella and Herlitz, 2021). In addition, there are drugs that cause AKI by affecting renal blood vessels and perfusion, but they are not covered in this review. In the subsequent text, the mechanism of Chinese meteria medica intervention in DI-AKI caused by several common nephrotoxic drugs based on the above three injury phenotypes is explored.

## 6 Mechanism of Chinese meteria medica intervention in DI-AKI

### 6.1 Cisplatin-induced AKI (CP-AKI)

**FIGURE 3:**
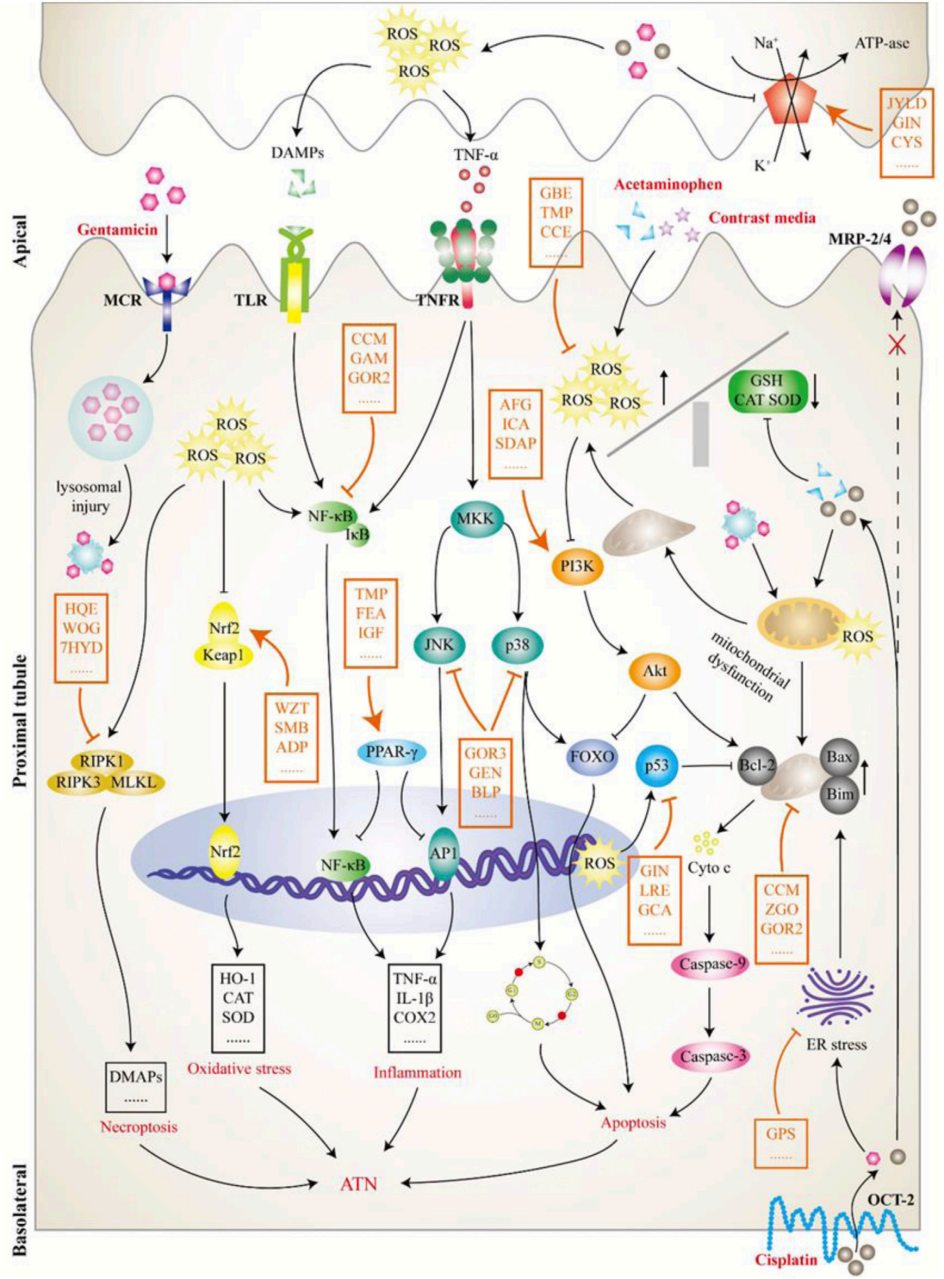
The pathological mechanism of acute tubular necrosis (ATN). This review focused on the induced ATN caused by four drugs: cisplatin, gentamicin, acetaminophen, and contrast media. Cisplatin (lower right) is transported to tubular cells *via* organic cation transporter 2 on the lateral side of the basolateral and excreted by apical transporters, including multidrug resistance protein transporter 2/4, to the tubular lumen. However, increased uptake or insufficient excretion can lead to cisplatin accumulation in cells, producing a variety of substances, including reactive oxygen species (ROS) and tumor necrosis factor α, and leading to mitochondrial dysfunction and tubular damage. Gentamicin (upper left) is attracted to an anionic phospholipid membrane in the tubular lumen and endocytoses into cells by interacting with megalin-cubilin receptor on the apical surface. Intracellular gentamicin is transported to lysosomes, resulting in lysosomal injury with myeloid formation and further leading to a mitochondrial injury cascade. Contrast media (upper right) has a direct toxic effect on tubular cells and can cause vasoconstriction and hypoxia in the renal medulla, which further leads to enhanced ROS formation and mitochondrial dysfunction. Acetaminophen (upper right) mainly inhibits prostaglandin synthesis and leads to hemodynamic changes, further tubular deficiency, and hypoxia, thereby activating a series of pathological processes, such as oxidative stress. Although the initial pathological processes of TN caused by the above nephrotoxic drugs are different, they all activate or inhibit a variety of downstream signaling molecules or pathways, thereby affecting pathological processes, such as cell death and inflammation. As shown in this figure, the intervention effect of Chinese meteria medica (orange mark) on ATN is a complex process involving multiple pathways, targets, and components. ADP, Andrographis paniculata; AFG, arginyl-fructosyl-glucose; BLP, butyl pyroglutamate; CCE, Curcuma comosa extract; CCM, curcumin; CYS, Cordyceps sinensis; FEA, ferulic acid; GAM, Ganoderma lucidum; GBE, ginkgo biloba extract; GCA, glycyrrhizic acid; GEN, genipin; GIN, ginseng; GOR2, ginsenoside Rh (2); GOR3, ginsenoside 20(S)-Rg3; GPS, ginseng polysaccharide; HQE, Huaiqihuang extractum; ICA, icariin; IGF, iridoid glycosides fraction; JYLD, jian-pi yi-qi li-shui decoction; LRE, licorice extract; MCR, megalin-cubilin receptor; MRP, multidrug resistance protein transporter; OCT2, human organic cation transporter 2; SDAP, sika deer antler protein; SMB, Salvia miltiorrhiza Bge.; TMP, tetramethylpyrazine; WOG, wogonin; WZT, WZ tablet; ZGO, zingerone; 7HYD, 7-hydroxycoumarin.

Cisplatin is a highly potent broad-spectrum chemotherapy agent whose accumulation in proximal tubular epithelial cells (PTECs) can cause the inflammation, damage, and death of cells, causing 30%–40% receptive cisplatin-treated patients to suffer from nephrotoxicity (Miller et al., 2010). As shown in Figure 3, cisplatin is transported into cells through human organic cation transporter 2 (OCT2) in the basolateral region of PTECs and excreted into the small lumen through various apical transporters. Owing to increased uptake or a lack of external drainage transferred into the urine, cisplatin accumulation in cells leads to nephrotoxicity by producing many substances, such as reactive oxygen species (ROS) and tumor necrosis factor α (TNF-α), thereby promoting mitochondrial toxicity and injury (Volarevic et al., 2019).

Under normal circumstances, oxidation and antioxidant systems work together to maintain the dynamic balance of ROS in the body. The accumulation of cisplatin in PTECs leads to downregulation of the antioxidant system, resulting in increased ROS production and oxidative stress (Hosohata, 2016; Perazella, 2019). Cisplatin can also cause mitochondrial respiratory chain dysfunction, thereby increasing ROS production (Kruidering et al., 1997). In addition to causing damage to various organelles within the cell, oxidative stress is involved in the activation of a series of downstream signaling pathways. Therefore, many botanical drugs have an interventional effect on cisplatin-induced AKI (CP-AKI) in the regulation of oxidative stress. Curcuma comosa extract protects against cisplatin-induced tubular necrosis in mice by increasing the renal glutathione (GSH) content as well as superoxide dismutase (SOD), GSH peroxidase (GPx), and catalase (CAT) activity (Jariyawat et al., 2009). Malondialdehyde (MDA) is a product of lipid and oxygen radical formation, and its content represents the degree of lipid peroxidation, which is related to damage to cell membranes, lipoproteins, and other lipid-containing structures (Sies, 1997). Many botanical drugs, such as Salviae Radix extract, Rubia cordifolia extract, and aged garlic extract, not only improve the activity of the antioxidant system in the body but also inhibit lipid peroxidation, which is represented by MDA (Jeong et al., 2001; Joy and Nair, 2008; Nasr and Saleh, 2014). NF-E2-related factor 2 (Nrf2) is an important regulator of antioxidant systems; however, under basic conditions, Keap1/Nrf2 complexes are easily degraded by ubiquitination. However, under oxidative stress conditions, Keap1 is oxidized and Nrf2 is introduced into the nucleus and binds to the antioxidant reaction element in the gene promoter region, initiating the transcription of a range of antioxidant factors, such as heme oxidase-1 (HO-1) and quinone oxidoreductase 1 (NQO1) (Saito, 2013; Suzuki and Yamamoto, 2015). Both WZ tablets (a preparation of an ethanol extract of Schisandra sphenanthera) and Salvia miltiorrhiza Bge. Can promote the nuclear accumulation of Nrf2 and the expression of its target genes HO-1 and NQO1, thereby exerting a protective effect against cisplatin-induced nephrotoxicity (Jin et al., 2015; Cao et al., 2017). As mitochondria-rich organs, the kidneys are extremely susceptible to oxidative stress-mediated injury; therefore, reducing mitochondria-derived ROS may be another important way to protect the kidneys from oxidative stress injury (Gorin, 2016). Cisplatin increases the expression of mitochondrial fission 1 protein (FIS1) and decreases the expression of optic atrophy 1 protein (OPA1), prompting the transition of mitochondrial homeostasis to fission. Curcumin attenuates FIS1 level increments and ultrastructural changes, restores OPA1 levels, and, thus, restores mitochondrial dynamic imbalances (Morigi et al., 2015).

Inflammation is thought to play an important role in the pathogenesis of DI-AKI, and all immune cells, such as neutrophils, monocytes/macrophages, and NK cells, are involved to varying degrees (Akcay et al., 2009; Rabb et al., 2016). Activation of the inflammatory process in DI-AKI is caused by a variety of pathways. In CP-AKI models, initial injury occurs in the tubular epithelium of the kidneys, and these injuries induce inflammatory mediators (including inflammatory factors, chemokines, and adhesion factors) (Manohar and Leung, 2018). In addition, oxidative stress promotes inflammation, and cell damage caused by inflammation further exacerbates oxidative stress (Tucker et al., 2015). In a CP-AKI model, pomegranate rind extract and loganin not only reduced oxidative stress, but also inhibited the release of inflammatory factors, such as TNF-α, interleukin (IL)-1β, and IL-6 (Karwasra et al., 2016; Kim et al., 2021). Activated nuclear factor-κB (NF-κB) is a key survival and inflammatory cell transcription factor that is induced by cisplatin (Ozkok et al., 2016). TNF-α can upregulate the activation of NF-κB and positive feedback upregulation of the expression of inflammatory factors to form a vicious circle that aggravates the inflammatory response (Moon et al., 2010). Asiatic acid prevents CP-AKI by anti-inflammatory mechanisms, which may be associated with the inhibition of the expression of pro-inflammatory cytokine IL-1β, TNF-α, monocyte chemotactic protein-1 (MCP-1), caspase-1, and NF-κB activation (Yang et al., 2018). Toll-like receptors (TLRs) are also considered to be potential drivers of cisplatin-induced pathology and toxicity. Cisplatin acting on PTECs causes the release of damage-associated molecular pattern molecules (DAMPs), which can excite TLRs and further exacerbate inflammation by activating the NF-κB pathway (Volarevic et al., 2019). In addition, MAPK signaling cascade activation, represented by c-Jun N-terminal kinase (JNK) and activation protein 1 (AP-1), is also closely related to the production of inflammatory mediators (Francescato et al., 2007; Jung et al., 2020). Peroxisome proliferation activation receptors (PPARs) are members of the ligand-activated nuclear transcription factor superfamily and can inhibit the inflammatory response by competing with inflammatory signaling pathways, such as AP-1 and NF-κB (Ju et al., 2020). TLR4/NF-κB signaling was significantly upregulated in CP-AKI rats, PPAR-γ expression was significantly reduced, and early administration of tetramethylpyrazine (TMP) significantly reversed these changes (Michel and Menze, 2019). In conclusion, inhibition of the inflammatory process in CP-AKI is a potential intervention mechanism.

In addition to oxidative stress and inflammatory damage, programmed cell death, such as apoptosis, necroptosis, and autophagy, also plays an important role in the pathogenesis of CP-AKI. Apoptosis refers to the biochemical process by which a cell is broken down by a specific set of proteins that interact with and program death-inducing signals. When a cell receives an apoptosis signal, it activates the initial caspase through different signaling pathways, reactivates the effector caspase, and degrades the associated substrate, ultimately leading to apoptosis (Chota et al., 2021). Cisplatin-induced apoptosis has been recognized to play an important role in CP-AKI, while inhibiting apoptosis is an effective strategy to improve kidney injury (Tsuruya et al., 2003). The mitochondrial apoptosis pathway is an important pathway for apoptosis, and caspase 3 plays a key role in the development of mitochondrial apoptosis (Kim et al., 2018). The B-cell lymphoma-2 (Bcl-2) family of molecules is involved in upstream regulatory pathways for the reception and transmission of apoptotic signals. When apoptotic proteins receive an apoptotic signal, they release cytochrome C from the mitochondria, activate downstream caspase 3, and cause apoptosis. In tubular epithelial cells, members of the Bcl-2 family, Bax and Bak, lead to increased mitochondrial membrane permeability, and Bcl-2 and Bcl-XL combat this membrane attack effect (Youle and Strasser, 2008). Ginsenoside Rk1 and Terminalia chebula extract can reverse cisplatin-induced elevated levels of Bax, caspase 3, and caspase 9, and decrease the levels of Bcl-2 in human embryonic kidney 293 (HEK-293) cells and rats (Kalra et al., 2019; Hu et al., 2020). Multiple signaling molecules or pathways are involved in the regulation of mitochondrial apoptosis. The phosphatidylinositol3-kinase (PI3K)/protein kinase B (Akt) pathway mitigates apoptosis, and PI3K knockout leads to excessive apoptosis in renal tubular epithelial cells, indicating its presence in CP-AKI, supporting the key role of kidney function (Kuwana et al., 2008). The specific PI3K inhibitor LY294002 eliminates the anti-apoptotic effect of icariin, indicating that icariin partially inhibits apoptosis *via* the PI3K/Akt pathway to prevent cisplatin-induced HEK-293 cell damage (Zhou et al., 2019). The mitogen-activated protein kinase (MAPK) pathway may be one of the causes of cisplatin-induced apoptosis, and galangin can inhibit apoptosis and improve CP-AKI by inhibiting the expression of MAPK pathways, including p38, JNK, and extracellular regulated protein kinases (ERK) (Tomar et al., 2017; Jung et al., 2020). The tumor protein p53 is a key molecule for cisplatin to induce apoptosis, and the activation of p53, as well as an unbalanced mitochondrial state, can activate caspase 3 and induce apoptosis in tubular cells (Overstreet et al., 2022). Some botanical drugs that protect CP-AKI from kidney damage have been linked to modulating p53 to inhibit apoptosis, such as Trichosanthes kirilowii extract and ginsenoside 20(S)-Rg3 (Seo et al., 2015; Han et al., 2016). Necroptosis is a new form of programmed cell death observed in many AKI models. Blocking necroptosis pathway molecules, including receptor-interacting protein 1 (RIPK1), RIPK3, and mixed lineage kinase domain-like protein (MLKL), significantly inhibits necroptosis and mitigates kidney injury (Linkermann et al., 2012; Lu et al., 2020). In cisplatin-induced mouse CP-AKI models, wogonin inhibited necroptosis *via* the RIPK1/RIPK3/MLKL pathway, effectively preventing kidney damage and decreasing renal function (Meng et al., 2018). In addition, selective degradation of damaged mitochondria through autophagy may also be a potential therapeutic strategy for CP-AKI, but current research is insufficient. The protective effect of Panax notoginsenoside on cisplatin-induced nephrotoxicity is mainly due to its ability to enhance mitophagy in kidney tissue through the hypoxia inducible factor-1α (HIF-1α)/BCL2 interacting protein 3 (BNIP3) pathway, significantly improving the expression of autophagy-related genes microtubule-associated protein light chain 3II (LC3II)/LC3I, autophagy protein 5 (Atg5), and Beclin-1 (Liu et al., 2015).

In summary, studies show that the core links of Chinese meteria medica intervention in CP-AKI are mainly oxidative stress, inflammation, and apoptosis processes. There is growing evidence that apoptosis is an important mechanism underlying cisplatin-induced nephrotoxicity. The effect of Chinese meteria medica on endogenous mitochondrial apoptosis has been proven, but it is not known whether they play a role in the apoptosis pathway of exogenous and endoplasmic reticulum stress (ERS).

### 6.2 Gentamicin-induced AKI (GM-AKI)

Gentamicin is the most commonly used aminoglycoside antibiotic and is mainly removed by glomerular filtration, where 10%–20% of the drug is taken up into PTECs through the megalin or cubilin receptor (Nagai and Takano, 2014; Hori et al., 2017). The cationic structure of gentamicin depends on the number of amino groups and their distribution within the molecule, which appear to play an important role in its toxicity (Jospe-Kaufman et al., 2020). Megalin is a ligand of many low-molecular weight proteins, including albumin, retinol-binding protein, alpha-1-microglobulin, and β2-microglobulin, and is highly expressed in PTECs, which also explains the cellular and tissue specificity of this toxicity (Christensen and Birn, 2001). In PTECs, gentamicin accumulates in lysosomes, Golgi, endoplasmic reticulum, and cell membranes, and inhibits phospholipase activity, leading to lysosomal phospholipid disease (Lopez-Novoa et al., 2011). Lysosomal structural damage and cell membrane instability eventually lead to the leakage of gentamicin into the cytoplasm. Gentamicin in the cytoplasm can act directly or indirectly on mitochondria, activating endogenous mitochondrial apoptosis or necrosis. Mitochondrial injury also leads to electron transport chain dysfunction, promotes the production of ROS, and leads to oxidative stress (Golstein and Kroemer, 2007; McWilliam et al., 2017). As with other cytotoxic drugs, such as cisplatin, the specific phenotype of death induced by gentamicin may depend on the concentration of the drug in the cell (El Mouedden et al., 2000). In addition, gentamicin inhibits endoplasmic reticulum function and protein synthesis, leading to ERS and the activation of apoptosis pathways (Peyrou et al., 2007). Gentamicin can also reduce renal blood flow, leading to renal parenchymal ischemia and further exacerbating acute tubular injury (Seçilmiş et al., 2005).

Overall, the core mechanism of gentamicin-induced gentamicin-induced AKI (GM-AKI) involves mitochondrial injury, which induces cell death. The subsequent oxidative stress and inflammatory processes exacerbate this cellular injury. Gentamicin significantly induces apoptosis in NRK-52E cells in a dose-dependent manner, whereas pretreatment with TMP inhibits the release of cytochrome c, inhibits the activation of caspase-3/8/9, increases the expression of Bcl-XL, inhibits the activation of NF-κB, and reduces ROS production, suggesting that TMP may be able to inhibit apoptosis and inflammatory and oxidative stress processes to relieve tubular injury in GM-AKI (Juan et al., 2007). As mentioned earlier, PPAR-γ activation inhibits the transduction of multiple inflammatory signaling pathways, including NF-κB. The anti-inflammatory and renal protective effects of ferulic acid on GM-AKI rat models may be exerted through PPAR-γ agonist activity (El-Ashmawy et al., 2018). Pharmacological activation of Nrf2 can be used as an important method to delay oxidative stress. Daphnetin can upregulate the expression of Nrf2 and its regulated antioxidant enzymes HO-1, NQO1, glutamate cysteine ligase, catalytic (GCLC), and glutamate cysteine ligase, modifier subunit (GCLM), *in vivo* and *in vitro*, thereby protecting against oxidative stress damage in GM-AKI (Fan et al., 2021).

### 6.3 Contrast-induced AKI (CI-AKI)

Contrast-induced AKI (CI-AKI) refers to AKI that appears after the use of iodized contrast agents, also known as contrast-induced nephropathy (Golshahi et al., 2014). Since being reported in 1955, CI-AKI has become the third leading cause of hospital-acquired kidney injury (Faucon et al., 2019). CI-AKI is common in up to 15% of patients with risk factors, including diabetes, cardiovascular disease, diuretic use, hypertension, and hyperuricemia, while the incidence in general patients is ≤1% (Mamoulakis et al., 2017). In addition to patient-specific factors, both the quantitative and qualitative features of contrast media may influence the incidence of CI-AKI. Higher contrast media usage and osmotic pressure are now generally considered to be positively correlated with the risk of CI-AKI (Owen et al., 2014; Li and Pan, 2022). All studies related to CI-AKI included in this review used hypotonic non-ionic contrast media, such as iopamidol, iohexol, iopromide, and ioversol (Supplementary Table S4). CI-AKI can increase the risk of cardiovascular and renal adverse events and long-term mortality. At present, there is no effective preventive measure to intervene in the occurrence of CI-AKI, and hydration therapy is still a commonly used preventive treatment clinically; therefore, it is necessary to urgently explore the pathogenesis of CI-AKI to seek more effective treatments (Mandurino-Mirizzi et al., 2022). The pathogenesis of CI-AKI is complex and has not yet been fully elucidated; however, the main mechanisms include renal medullary ischemia and direct toxicity of contrast media to tubular epithelial cells. Renal medullary ischemia in CI-AKI is associated with local vasoconstriction, osmotic diuresis, decreased renal blood flow, and increased tubular oxygen consumption (Sendeski et al., 2009; Caiazza et al., 2014). Another important pathogenic mechanism is that contrast media can directly cause cytotoxicity in tubular epithelial cells and endothelial cells, leading to mitochondrial dysfunction, apoptosis or necrosis, and interstitial inflammation (Quintavalle et al., 2011; Andreucci et al., 2014). In addition, medullary hypoxia leads to enhanced ROS formation, which can lead to mitochondrial dysfunction and oxidative stress (Heyman et al., 2010).

Mitochondrial dysfunction and oxidative stress play important roles in the pathophysiology of CI-AKI. Therefore, reducing oxidative stress and protecting mitochondrial function are potential strategies for preventing CI-AKI. Phyllanthus emblica extract mitigates the extent of pathological damage to the kidneys in CI-AKI rats, and its effects may be associated with alleviating oxidative stress, including decreasing MDA levels and increasing total antioxidant capacity (TAC), SOD, and CAT levels (Tasanarong et al., 2014). The induction of ROS in CI-AKI activates the MAPK signaling pathway, including JNK and p38, contributing to caspase-3/9 activation, thus, inducing apoptosis (Pisani et al., 2013). Upregulation of p38 MAPK phosphorylation, elevated caspase-3 activity, increased Bax expression, and decreased Bcl-2 expression can be observed in the kidney tissue of CI-AKI rats, but these changes can be prevented by astragaloside IV pretreatment (Gui et al., 2013). Contrast media administration also upregulates PPAR-γ co-activator-1α-fork-cassette transcription factor 1 (PGC-1α-FoxO1) signaling-mediated oxidative stress and apoptosis, and this process can be reversed by TMP (Gong et al., 2013; Hong et al., 2017). Apoptosis is closely associated with mitochondrial dynamics. Previous studies have shown that in the early stages of apoptosis, Bax is transferred from the cytoplasm to the mitochondria before caspase activation, while dynein-related protein 1 (DRP1) is also transferred from the cytoplasm to the mitochondrial division site, which then mediates mitochondrial division (Jiang and Okazaki, 2022). Inhibition of Drp1 activity inhibits mitochondrial division, caspase activation, and apoptosis (Hoppins and Nunnari, 2012). Additionally, high expression of mitochondrial fusion protein 1 (Mfn1) and Mfn2 can inhibit apoptosis (Jian et al., 2018). TMP can improve abnormal mitochondrial dynamics by upregulating Mfn2 and downregulating Drp1, as well as mitigating apoptosis of tubular epithelial cells caused by contrast medias (Gong et al., 2019). According to existing research, the mechanism of botanical drugs in alleviating CI-AKI is mainly protecting against mitochondrial dysfunction and alleviating oxidative stress.

### 6.4 Acetaminophen-induced AKI (AP-AKI)

Acetaminophen belongs to the NSAIDs and is one of the commonly used antipyretic analgesics in clinical practice. NSAID-related AKI accounts for 7% of all AKI cases and 35% of DI-AKI (Musu et al., 2011). Inhibition of renal prostaglandin (PG) synthesis is the primary mechanism of NSAID-related AKI (Lucas et al., 2019). Depending on the drug, there are two main types of AKI after renal PG synthesis is inhibited: ATN and AIN (Caravaca-Fontán et al., 2019). In general, ATN is dose-dependent in NSAID-related AKIs, whereas AIN is a non-dose-dependent idiosyncratic response. Acetaminophen-induced AKI (AP-AKI) is also caused by excessive or long-term use of acetaminophen; therefore, its pathology is usually ATN (Hiragi et al., 2018). The pathological mechanism of AP-AKI is very complex, and mainly includes oxidative stress, mitochondrial dysfunction, inflammatory response, ERS, and glomerular hemodynamic changes, and deeper molecular mechanisms need to be further studied (Lorz et al., 2004; Lorz et al., 2005; Hua et al., 2018). Oxidative stress is currently known to be one of the most critical factors in AP-AKI. ROS further trigger apoptosis through the Bcl-2 and caspase families, indicating severe cell injury (Hosohata, 2016). Acetaminophen can lead to a significant decrease in SOD and GPx activity and elevated expression of caspase-3 and NF-κB in the kidney tissue, whereas corn silk methanolic extract treatment reverses these changes and activates the transcription of Nrf2 to combat drug-induced oxidative stress and apoptosis (Wans et al., 2021). Acetaminophen also inhibits the PI3K/Akt pathway, leading to FoxO1 nuclear translocation, which activates Bax and inhibits Bcl-2 to exacerbate the apoptosis of tubular epithelial cells. Sika deer antler protein can reduce the occurrence of the apoptosis cascade by activating the PI3K/Akt pathway to inhibit the nuclear translocation of FoxO1 (Ruan et al., 2019). In conclusion, oxidative stress and apoptosis remain the main targets of botanical drugs intervention in AP-AKI, and the role of other mechanisms needs to be further elucidated.

### 6.5 Methotrexate-induced AKI (MTX-AKI)

**FIGURE 4:**
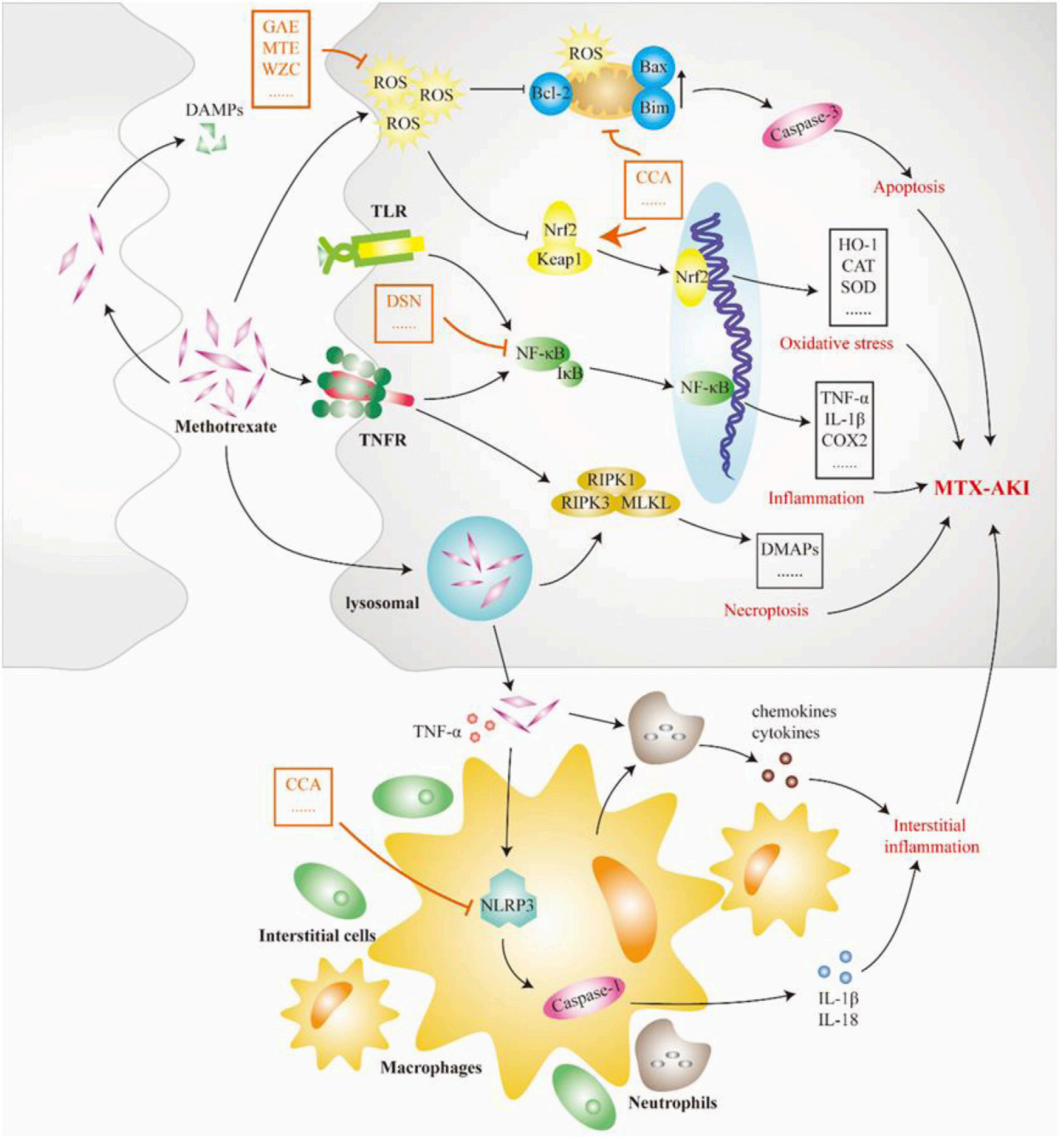
Pathological mechanism of GN caused by methotrexate (MTX). MTX crystal deposition in the renal tubules causes tubular blockage and induces tubular cell death by activating many pathways. Lysosomes become instable and lysopage after phagocytosis of MTX crystals, further triggering the formation of the receptor-interacting protein kinase 3–mixed lineage kinase domain-like protein complex, leading to renal tubular cell necroptosis and the release of damage-associated molecular patterns (DAMPs). DAMPs again induce Toll-like receptor-dependent inflammation and cell necrosis. In addition, MTX crystals in the renal stromitium stimulate macrophages, dendritic cells, and neutrophils, resulting in the production of cytokines and chemokines, including interleukin (IL)-1β and IL-18, which further leads to tubular damage and increased inflammation. Therefore, limiting the development of inflammatory processes is an important way for Chinese meteria medica (orange marker) to intervene in MTX-induced acute kidney injury. CCA, chicoric acid; DAMP, damage-associated molecular pattern; DSN, dioscin; GAE, garlic aqueous extract; MLKL, mixed lineage kinase domain-like protein; MTE, mistletoe extract; NLRP3, NOD-like receptor thermal protein domain associated protein 3; RIPK1, receptor-interacting protein kinase 1; TLR, Toll-like receptor; WZC, Wuzhi capsule.

MTX is an antifolate antitumor drug that can be used in the treatment of most patients with malignant tumors, but it can also cause serious toxic side effects in multiple systems, common gastrointestinal reactions, skin and mucosal damage, liver and kidney function damage, bone marrow suppression, *etc.*, of which kidney injury is the most important (Widemann and Adamson, 2006). A recent study in Spain showed that approximately 11.6% of 1,475 patients who received high-dose MTX treatment each year developed MTX-induced AKI (MTX-AKI) (Gros et al., 2022). The current view is that most nephrotoxicity of MTX is CN, mainly due to the precipitation of MTX and its terminants in the renal tubules (Howard et al., 2016). The nephrotoxicity of MTX is thought to be closely related to two factors. First, MTX causes arteriolar vasoconstriction due to decreased renal perfusion. Second, as shown in Figure 4, MTX is taken up by the tubules and causes direct tubular toxic injury (Perazella and Herlitz, 2021). At the same time, the occurrence of MTX-AKI further leads to delayed excretion of MTX, so that tubular cells are exposed to high doses of MTX for a longer time, which exacerbates renal toxicity, thereby aggravating delayed MTX excretion and circulating malignancy (Ramsey et al., 2018). Precipitated MTX crystals block the lumen and induce necrosis of TECs by activating multiple pathways. State instability after lysosomal uptake of MTX crystals or direct stimulation of crystals degrades RIPK1 and triggers the formation of RIPK3-MLKL necrotic complexes, resulting in the necroptosis of TECs. Necroptosis stimulates the release of DAMPs, thereby inducing TLR/NF-κB signal-dependent inflammation and cell necrosis while promoting the secretion of chemokines and cytokines (Elmansy et al., 2021). MTX crystals deposited in the renal interstitium are engulfed by macrophages or dendritic cells and activate the nucleotide-binding domain-like receptor protein 3 (NLRP3) inflammasome, promoting IL-1β, IL-18, and other cytokines (Komada and Muruve, 2019). The production of these cytokines and chemokines exacerbates tubular injury and interstitial inflammation. Overall, the auto-amplification of intrarenal necrosis and inflammation induced by MTX crystals may be the core mechanism of kidney injury in MTX-AKI. Mitochondrial dysfunction and oxidative stress also play a role in the mechanism of MTX nephrotoxicity. MTX treatment increases ROS levels, reduces tissue antioxidant capacity, increases lipid peroxidation, and depletes GSH reserves (Abraham et al., 2010). MTX treatment also increases mitochondrial permeability and respiratory chain deletion, leading to the activation of apoptosis and further accumulation of ROS (Heidari et al., 2018). Chicoric acid can inhibit the levels of NF-κB, NLRP3, caspase-1, and IL-1β in the kidney tissue of rats with MTX-AKI while reversing Bcl-2 and the expression of Bax and caspase-3, thus, exerting anti-inflammatory effects and inhibiting apoptosis (Abd El-Twab et al., 2019). The Keap1/Nrf2 complexes are important antioxidant regulatory systems in cells. Dioscin alleviates oxidative stress damage in MTX-AKI by directly targeting the expression of miR-145–5p in Sirt5 and modulating the expression levels of SOD1, Nrf2, GST, HO-1, GCLC, and NQO1 (Li et al., 2021).

## 7 Botanical drug metabolites with research potential

### 7.1 Ginseng saponins

**FIGURE 5:**
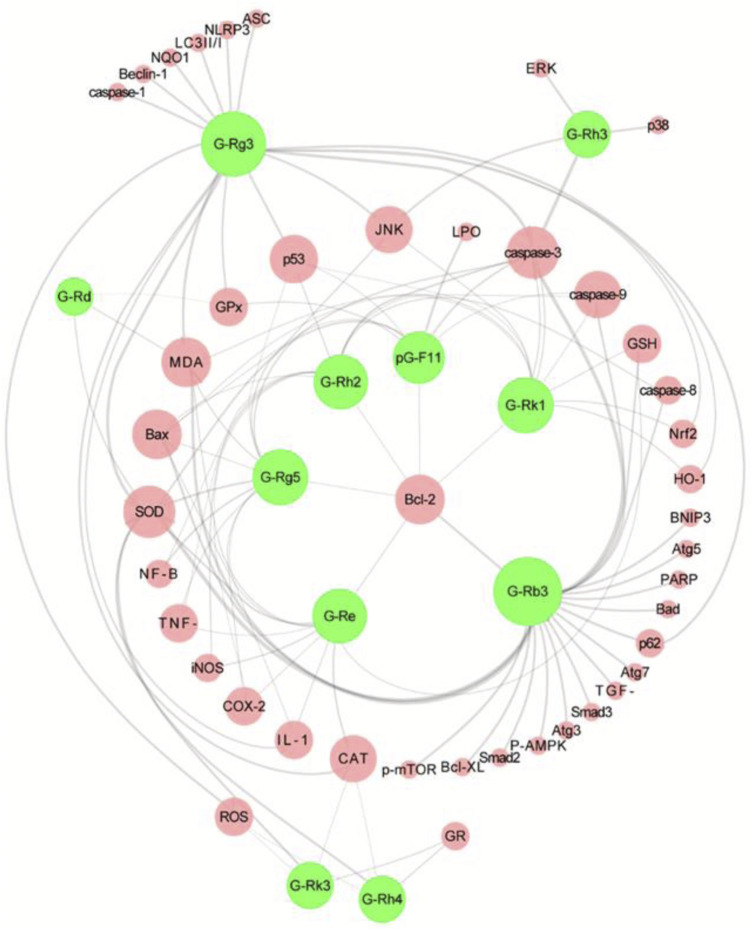
Overview of the molecular targets of 11 ginsenoside interventions in cisplatin-induced acute kidney injury (CP-AKI). Ginsenosides Rg3 and Rb3 are the most studied interventions, which suggests that they have high potential applications. The pharmacological effects of other ginsenosides in CP-AKI need to be further explored. G-Rg3, ginsenoside Rg3; G-Rg5, ginsenoside Rg5; G-Rk1, ginsenoside Rk1; G-Rh2, ginsenoside Rh2; G-Rd, ginsenoside Rd; G-Rk3, ginsenoside Rk3; G-Rh4, ginsenoside Rh4; G-Rh3, ginsenoside Rh3; G-Re, ginsenoside Re; G-Rb3, ginsenoside Rb3; pG-F11, pseudoginsenoside F11.

Ginseng is the dried root and rhizome of the Araliaceae plant *Panax ginseng* C.A.Mey [Araliaceae], also known as Korean ginseng (Choi et al., 2013). As an important botanical drugs, ginseng has been used in East Asian countries for thousands of years. In TCM, ginseng is used as a reinforcing drug, primarily for qi deficiency patterns. The Chinese Pharmacopoeia contains suncured ginseng (SGS) and red ginseng (RGS), of which SGS refers to ordinary dry ginseng and RGS is obtained by single steaming and sunbathing. Black ginseng (BGS) is an uncommon ginseng product that is mainly prepared by multiple steaming and drying processes or combined with microbial fermentation processing (Metwaly et al., 2019). Ginseng contains many active ingredients, of which ginsenosides are undoubtedly the characteristic metabolites that contribute the most to its pharmacological effects (Kang et al., 2013; Shin et al., 2015). Additionally, some ginsenosides are found in other Araliaceae plants, such as *Panax quinquefolius* L. and *Panax notoginseng* (Burk.) F. H. Chen. As shown in Supplementary Materials, 11 ginsenosides have been extensively studied in the CP-AKI model: ginsenosides Rg3, Rg5, Rk1, Rh2, Rd, Rk3, Rh4, Rh3, Re, and Rb3 as well as pseudoginsenoside F11. Among the above metabolites, some are rare saponins from RGS and BGS, such as ginsenosides Rh3, Rg5, Rk1, Rh2, and Rg3, which have received increasing attention because of their broader and more efficient pharmacological activities (Li et al., 2022). In summary, these ginsenosides intervene in CP-AKI *via* inflammation, oxidative stress, and cell death (Figure 5). As a rare saponin, ginsenoside Rg5 has a multifaceted protective effect on cisplatin-induced nephrotoxicity in mice, increases SOD activity and GSH content to restore the antioxidant capacity of the kidneys, inhibits the expression of a series of inflammatory factors (including TNF-α, IL-1β, and COX-2) in the NF-κB pathway, and can also significantly inhibit the apoptosis of renal tubular cells (Li et al., 2016). In addition to inhibiting apoptosis, ginsenosides have a regulatory effect on autophagy. Ginsenoside Rg3 improves kidney damage in HK-2 cells and mice by upregulating LC3II/I and Beclin-1 while inhibiting p62, NLRP3, caspase-1, and IL-1β, suggesting that activating the autophagy-mediated inhibition of NLRP3 may be a novel way to prevent CP-AKI (Zhai et al., 2021). Owing to the hemolytic effects of ginsenosides, they are often used for oral administration. Therefore, oral availability is another important factor that affects the activity of ginsenosides. In general, raw ginsenosides, such as Rb1, Rb2, and Rd, have lower oral availability, whereas rare ginsenosides, including Rg3, Rg5, and Rk1, may have higher absorption rates (Ryu et al., 2013). In addition, chemical modifications and derivative designs of ginsenosides can improve their bioavailability and activity (Ma et al., 2021). Overall, ginsenosides are promising anti-CP-AKI metabolites, and the correct combination of these ingredients may help improve this effect.

### 7.2 Tetramethylpyrazine (TMP)

**FIGURE 6:**
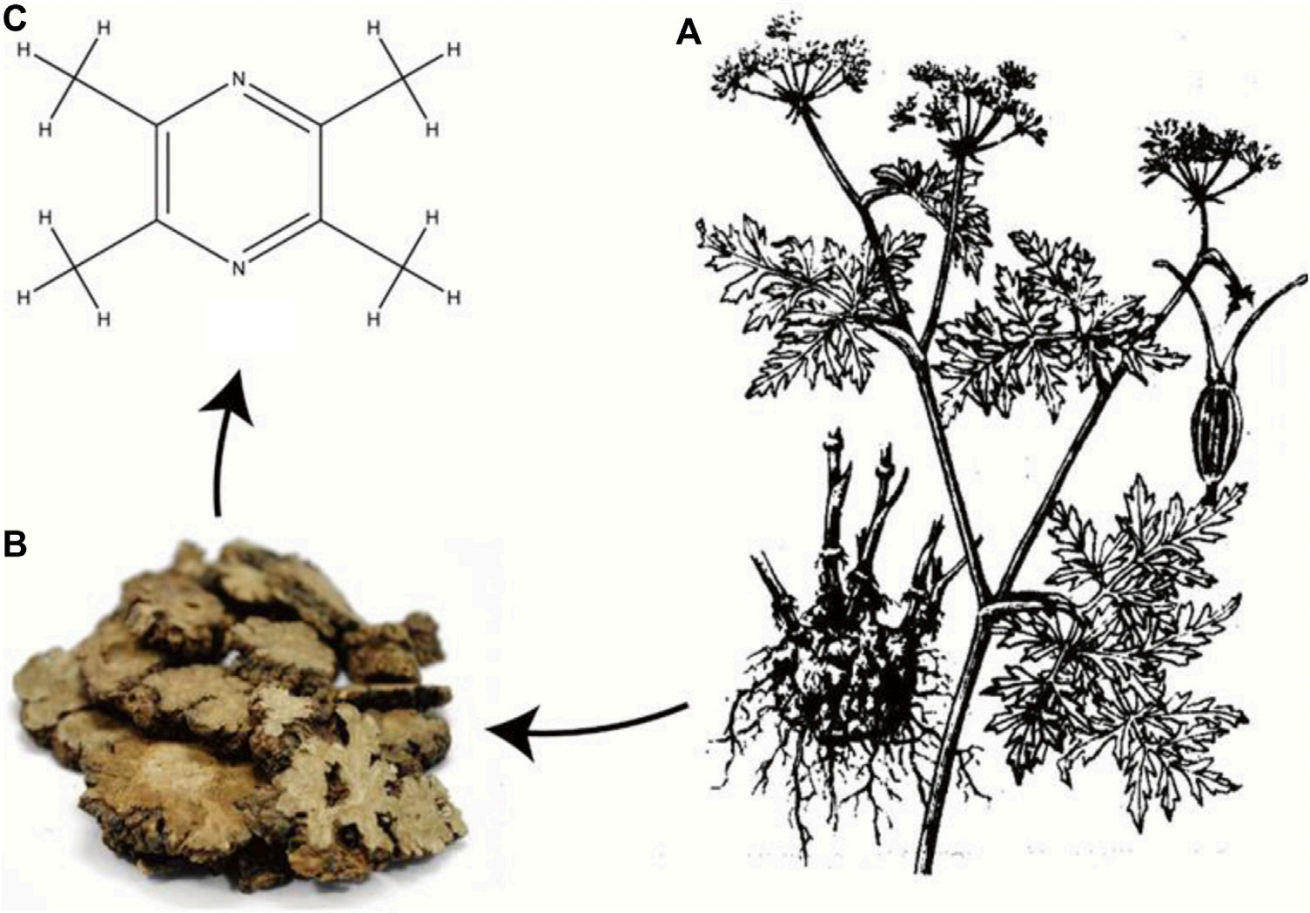
Pharmaceutical source of tetramethylpyrazine (TMP). TMP is derived from the rhizome of the botanical drug *Conioselinum anthriscoides* ‘Chuanxiong’ [Apiaceae] **(A)**. Chinese cut crude drug of Ligusticum chuanxiong hort **(B)**. The molecular structure of TMP **(C)**.

TMP is the active ingredient and characteristic alkaloid of the *Conioselinum anthriscoides* ‘Chuanxiong’ (Figure 6). TCM theory suggests that the effect of *C. anthriscoides* ‘Chuanxiong’ [Apiaceae] is that it invigorates blood and regulates qi. Pharmacological studies have found that TMP inhibits platelet aggregation, reduces blood viscosity, scavenges free radicals, protects cardiovascular and cerebrovascular vessels, and dilates renal blood vessels (Zhao et al., 2016). Given its antioxidant, anti-inflammatory, and anti-apoptotic effects, many studies have focused on the benefits of TMP in AKI (Li and Gong, 2022). Cisplatin causes an increase in MDA, Nitric Oxide (NO), and inducible nitric oxide synthase (iNOS) levels and a decrease in GSH, GST, and SOD levels, which can be restored to near-normal levels by TMP intervention, suggesting that TMP inhibits oxidative stress, thus, has a dose-dependent protective effect against cisplatin-induced tubular toxicity (Ali et al., 2008; Liu et al., 2008). The anti-inflammatory, anti-apoptotic, and antioxidant effects of TMP on GM-AKI, AP-AKI, and CI-AKI have been described above. Nephrotoxicity due to arsenic exposure remains an area of less concern, and studies have shown that TMP may protect the kidneys from sodium arsenite toxicity. Exposure to sodium arsenite can lead to oxidative stress, mitochondrial dysfunction, activation of NF-κB- and p38 MAPK-dependent pro-inflammatory signaling, and induction of apoptosis, whereas these pathological processes can be inhibited by adding TMP to HK-2 cells (Gong et al., 2015). Although the pathological mechanisms of AKI caused by various drugs differ, these data support the potential application of TMP as a new therapeutic drug for DI-AKI. Additionally, deficiencies in the molecular structure may limit the application of TMP. The piperazine ring is a key group for TMP molecules to exert pharmacological effects, but the methyl group in its side chain is easily excreted by oxidative metabolism, resulting in its short half-life and weakened pharmacological effects (Wang et al., 2019). Thus far, the development of TMP-related derivatives may provide a feasible solution to these problems.

### 7.3 Panax notoginseng saponins

*Panax notoginseng* saponins (PNSs) are derived from the rhizome of *Panax notoginseng* (Burkill) F.H.Chen [Araliaceae], a plant belonging to the family Araliaceae that contains a variety of monomeric saponins (mainly ginsenosides Rb1, Rg1, Rd, and Re as well as notoginsenoside R1) (Supplementary Materials) (Liu et al., 2020). Among the above metabolites, ginsenosides Rb1 and Rg1 as well as PNS R1 were selected as standard metabolites to evaluate the quality of *Panax notoginseng* (Burkill) F.H.Chen. Based on the TCM theory, *Panax notoginseng* (Burkill) F.H.Chen is primarily used to promote blood circulation and relieve pain (Qiao et al., 2018). Based on the included studies, PNSs may mitigate mitochondrial damage through a variety of pathways, thereby protecting renal tubular cells. PNSs supplementation was found to reduce apoptosis and apoptosis-related protein expression induced by cisplatin or cisplatin plus HIF-1α knockdown, suggesting that PNSs may pass the HIF-1α/BNIP3 pathway to inhibit mitochondrial apoptosis, thereby alleviating CP-AKI (Li et al., 2021). In a polymyxin E-induced nephrotoxicity model, PNSs could also inhibit oxidative stress and prevent apoptosis through the mitochondrial pathway (Zhang et al., 2019). PNSs has also been reported to mitigate downstream oxidative stress and mitochondrial injury *via* the autophagy pathway. In cisplatin-induced mitochondrial damage, PNSs enhances HIF-1α-mediated mitophagy and selectively removes damaged mitochondria, thereby reducing ROS production (Liu et al., 2015; Li et al., 2020). It is worth noting that some PNSs injections and special PNSs components have been shown to cause some biological toxicity at certain doses (Chen et al., 2014; Xu et al., 2019). Therefore, this issue needs to be approached with caution in PNSs studies of DI-AKI.

### 7.4 Curcumin

**FIGURE 7:**
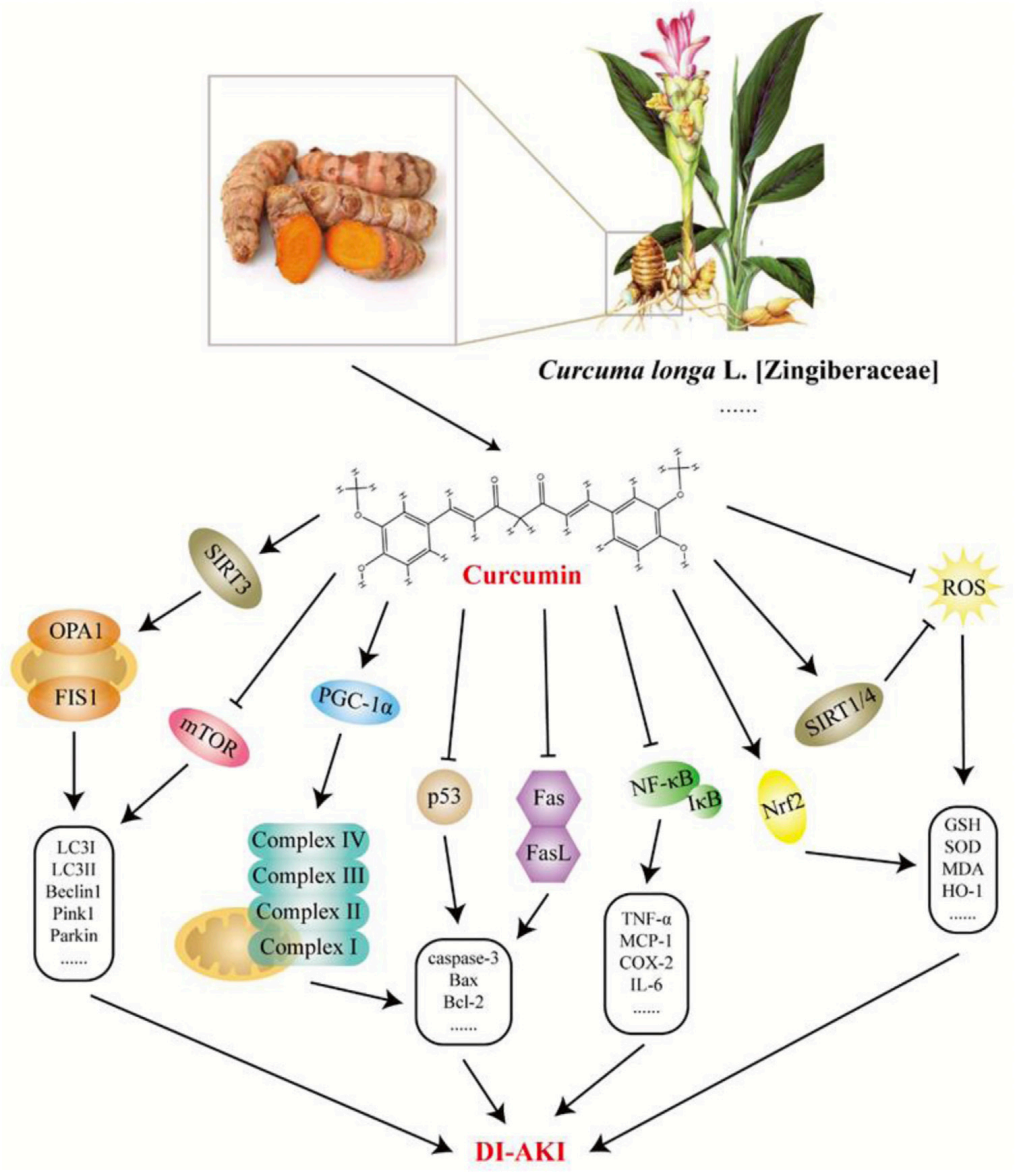
Potential mechanisms of curcumin intervention in drug-induced acute kidney injury (DI-AKI). Curcumin is mainly derived from the rhizome of *Curcuma longa* L. [Zingiberaceae], which has good anti-inflammatory, antioxidant, and other pharmacological effects. Based on existing research, curcumin exerts a protective effect on DI-AKI caused by cisplatin, gentamicin, doxorubicin, polymyxin E, cyclosporine A, and arsenic by regulating inflammation, oxidative stress, apoptosis, mitochondrial autophagy, *etc.*

Curcumin is a natural polyphenolic metabolite derived from a variety of Chinese meteria medica, including *Curcuma longa* L [Zingiberaceae], *Curcuma aromatica* Salisb. [Zingiberaceae], and *Curcuma zedoaria* (Christm.) Roscoe [Zingiberaceae]. Owing to its non-toxic and harmless characteristics, curcumin is widely used as a natural pigment in the food, textile, and cosmetic industries (Sohn et al., 2021). In the theory and practice of TCM, the abovementioned curcumin -rich botanical drugs promote blood circulation and eliminate blood stasis and have long been used to treat pain, inflammation, and other diseases. To date, an increasing number of studies have demonstrated the various biological properties of curcumin, including antioxidant, anti-inflammatory, anticancer, antiproliferative, pro-apoptotic, antiviral, and anti-atherosclerotic effects (Bolger et al., 2022). Here, the studies reviewed imply that curcumin has been extensively investigated in cisplatin-, gentamicin-, doxorubicin-, polymyxin E−, cyclosporine A-, and arsenic-induced nephrotoxicity. As shown in Figure 7, curcumin antagonizes the pathological process of DI-AKI through multiple pathways, including inflammation, oxidative stress, apoptosis, and mitochondrial autophagy. Sirtuins (SIRT) are a family of proteins composed of seven enzymes that play an important role in resisting stress and regulating cell death thresholds and appear to be important targets for the nephrotoxicity of curcumin mitigation drugs (Corsello et al., 2018). SIRT1 has also been shown to repair DNA breaks caused by oxidative stress. In GM-AKI, curcumin treatment exerts anti-apoptotic and antioxidant effects by upregulating Nrf2/HO-1 and SIRT1 expression (He et al., 2015). Similarly, curcumin increased SIRT protein (including SIRT1, SIRT3, and SIRT4) levels in cisplatin-treated rats, thereby reducing chemotherapy-induced nephrotoxicity (Ugur et al., 2015). SIRT3 is mainly localized to the mitochondria and catalyzes the deacetylation of mitochondrial proteins, which, in turn, affects the regulation of mitochondrial activity and energy metabolism (Afzaal et al., 2022). It has been found that the protective effect of curcumin in CP-AKI is related to the maintenance of mitochondrial bioenergetics, ultrastructure, and redox balance by regulating SIRT3 levels (Ortega-Domínguez et al., 2017). The modulatory effect of curcumin on inflammation is well-known (Peng et al., 2021). Curcumin significantly downregulated the level of mincle in CP-AKI kidneys, reduced the expression of iNOS, and inhibited the activation of Syk and NF-κB, indicating that it may inhibit M1 macrophages in a mincle-dependent mode (Tan et al., 2019). Curcumin is also involved in signaling during apoptosis in various ways. In the CP-AKI model, curcumin reduced the activation of cell death-related proteins Fas, Fas-L, and p53 to prevent the development of renal tubular apoptosis in rats (Topcu-Tarladacalisir et al., 2016). Curcumin also relieves gentamicin-induced nephrotoxicity by protecting the mitochondrial respiration complex to prevent mitochondrial dysfunction and apoptosis (Negrette-Guzmán et al., 2015). Despite its potential applications, numerous preclinical studies have reported its low bioavailability (Kunnumakkara et al., 2019). Fortunately, synthetic derivatives and various nanoforms of curcumin are being used in various biomedical applications, creating more space to solve this problem.

## 8 Discussion

Above, research on Chinese meteria medica and their metabolites to intervene in DI-AKI was reviewed in detail, and the highly researched toxic drugs and active ingredients were further discussed. As the most common pathological phenotype of DI-AKI, ATN is the focus of most studies related to Chinese meteria medica. According to the results of these studies, various botanical drugs and metabolites not only improve kidney function and reduce the level of kidney injury markers (including Kim-1, CysC, and NGAL), but also reduce the degree of pathological damage to the kidneys. Although the pathological mechanisms of DI-AKI caused by various nephrotoxic drugs are different, the preventive effect of Chinese meteria medica on DI-AKI is mainly exerted by regulating oxidative stress, inflammatory processes, and cell death. There have been many reports of links between DI-AKI and autophagy, most of which suggest a protective effect of autophagy on tubular epithelial cells. In CP-AKI, ginsenoside Rg3 effectively prevents cisplatin-induced kidney damage by activating the autophagy-mediated inhibition of the NLRP3 pathway, and this process can be blocked by autophagy inhibitor 3-methyladenine (Zhai et al., 2021). In another CP-AKI study, cisplatin induced autophagy activation, which was inhibited by ginsenoside Rb3, suggesting that autophagy adversely affected cisplatin-induced kidney damage. This paradoxical result also suggests that the role of autophagy in DI-AKI remains controversial, and that there may be more complex and elaborate regulatory mechanisms behind it. Overall, the evidence laid out in this review supports the potential of Chinese meteria medica as a therapeutic agent for DI-AKI. Research on Chinese Materia Medica for DI-AKI treatment mainly focuses on metabolites or single botanical drugs. Plant-based drug development utilizes metabolites and monomeric components of botanicals. However, the theoretical connotations and characteristics of TCM should not be ignored. Based on sufficient experimental data, future studies should consider appropriate combinations of the most beneficial botanical drugs or metabolites under the guidance of the TCM theory. As discussed above, ginseng saponins, tetramethylpyrazine, panax notoginseng saponins, and curcumin are the most widely studied botanical drug metabolites for DI-AKI treatment. Among them, ginseng extracts, ginsenosides, and their combinations have shown the most promising results. Their major treatment mechanisms involve antioxidant, anti-inflammatory, and anti-apoptotic effects as well as regulation of autophagic signaling. In addition to nephrotoxicity, ginseng and ginsenosides have shown good efficacy against cardio-, hepato-, neuro-, and reproductive toxicity (Hou et al., 2018; Zhang et al., 2018; Ren et al., 2019; Liu et al., 2021). It is important to note that the information presented here is mainly from preclinical studies, and most drugs or metabolites mentioned are not supported by double-blind clinical trials in disease settings. Therefore, more rigorous clinical research data are urgently needed.
