# Supplementary material for: Potential therapeutic effects of Chinese meteria medica in mitigating drug-induced acute kidney injury
Source: Front Pharmacol. 2023 Apr 3;14:1153297. doi: 10.3389/fphar.2023.1153297 (PMC10106589; doi:10.3389/fphar.2023.1153297)
Supplement: Supplementary file 1 [file Table1.DOCX]

**Supplementary materials**

**Table 1 Chemotherapy induced DI-AKI**

| **Type** | **Herbal/ Active ingredients/ Formula** | **Species, Source** | **Drug** | **Animal/Cell** | **Dose, Duration, Route of H/A/F** | **Dose, Duration, Route of drug** | **Main outcome** | **Histopathology**  **Score^a^** | **Study** |
| --- | --- | --- | --- | --- | --- | --- | --- | --- | --- |
| In vivo | jian-pi yi-qi li-shui decoction | - | cisplatin | male, female Wistar rats | 1 ml/d for 7d, ig | 0.3 mg/d for 3d, sc | SrCr↓, BUN↓, NAG↓, β2-mG↓, Na/K ATPase↑ | N | (Cheng, 1992) |
| In vitro | magnesium lithospermate B | *Salvia miltiorrhiza* Bunge [Lamiaceae] | cisplatin | LLC-PK1 cells | 2.5, 12.5, 25, 50,125 μM | 0.25 μM | LDH↑, MDA↓ | N | (Yokozawa et al., 1997) |
| In vivo | ginsenoside-Rd | *Panax ginseng* C.A.Mey [Araliaceae] | cisplatin | male Wistar rats | 100 mg/kg/d for 30d, ig | 6 mg/kg for single injection, ip | SrCr↓, BUN↓, MDA↓, SOD↑, GPx↑ | N | (Yokozawa and Liu, 2000) |
| In vivo and in vitro | Panax notoginseng saponins | *Panax notoginseng* (Burkill) F.H.Chen [Araliaceae] | cisplatin | rabbits/ rabbit proximal tubular cells | 100, 200 mg/kg/d for 6d, ig; 10/ 100mg for 24h | 5 mg/kg/d for 4d, ip | SrCr↓, BUN↓, cell viability↑, DNA interstrand cross-link↓, DNA-protein interstrand cross-link↓, cytosolic free [Ca2+]i overload↓ | N | (Liu and Zhou, 2000) |
| In vivo | Radix Salviae Miltiorrhizae extract | *Salvia miltiorrhiza* Bunge [Lamiaceae] | cisplatin | rabbits | 0.1 g/d/kg BW for 7d, ig | 5 mg/kg for single injection, ip | SrCr↓, MDA↓, LDH↓, GFR↑ | + | (Jeong et al., 2001) |
| In vivo and in vitro | ginsenoside Rd | *Panax ginseng* C.A.Mey [Araliaceae] | cisplatin | male Wistar rats/ LLC-PK1 cells | 1.5 mg/kg/d for 30d, ig; 25, 50, 125 μM | 6 mg/kg for single injection, ip | SrCr↓, BUN↓, DNA fragmentation↓ | N | (Yokozawa and Dong, 2001) |
| In vivo | Folium Ginkgo extract | *Ginkgo biloba* L. [Ginkgoaceae] | cisplatin | male SD rats | 100 mg/ml/kg/d for 10d, ig | 7 mg/kg for single injection, ip | SrCr↓, BUN↑, NO↓, MPO↓, MDA↓, MTP↓ | N | (Gulec et al., 2006) |
| In vivo | zingiber officinale Roscoe | *Zingiber officinale* Roscoe [Zingiberaceae] | cisplatin | male Swiss Albino mice | 250, 500 mg/kg for single dose, ig | 10 mg/kg BW for single injection, ip | SrCr↓, BUN↓, SOD↑, CAT↑, GPx↑, GSH↑, MDA↓ | N | (Ajith et al., 2007) |
| In vivo | curcumin | *Curcuma aromatica* Salisb. [Zingiberaceae] et al. | cisplatin | male Wistar rats | 15, 30, 60 mg/ml/kg/d for 5d, ig | 5 mg/kg for single injection, ip | SrCr↓, BUN↓, TNF-α↓, SOD↑, GSH↑, CAT↑, LPO↓ | + | (Kuhad et al., 2007) |
| In vivo | sesame oil | *Sesamum indicum* L. [Pedaliaceae] | cisplatin | female C57BL/6 mice | 0, 2, 4, 8 mL/kg for 3d, ig | 20 mg/kg for single injection, sc | SrCr↓, BUN↓, MDA↓, hydroxyl radical↓, peroxynitrite↓, nitrite↓ | + | (Hsu et al., 2007) |
| In vivo | tetramethylpyrazine | *Conioselinum anthriscoides* 'Chuanxiong' [Apiaceae] et al. | cisplatin | male Wistar  rats | 80 mg/kg/d for 7d, ig | 6 mg/kg for single injection, ip | SrCr↓, BUN↓, NAG↓, GSH↑, SOD↑, TOX↑ | + | (Ali et al., 2008) |
| In vivo | tetramethylpyrazine | *Conioselinum anthriscoides* 'Chuanxiong' [Apiaceae] et al. | cisplatin | male SD rats | 50, 100mg/kg/d for 7d, ip | 8mg/kg for single injection, iv | SrCr↓, BUN↓, MDA↓, NAG↓, SOD↑, GSH↑, GST↑, iNOS↓, NO↓ | N | (Liu et al., 2008) |
| In vivo | Radix Rubiae extract | *Rubia cordifolia* L. [Rubiaceae] | cisplatin | male Swiss Albino mice | 250, 500 mg/kg BW for single dose, ig | 12 mg/kg BW for single injection, ip | SrCr↓, BUN↓, GSH↑, GPx↑, SOD↑, MDA↓ | N | (Joy and Nair, 2008) |
| In vivo | Rhizoma Curcumae Longae extract | *Curcuma longa* L. [Zingiberaceae] | cisplatin | male ICR mice | 100-200 mg/kg BW for 4d, ig | 12.5 mg/kg BW for single injection, ip | SrCr↓, BUN↓, GSH↑, GPx↑, SOD↑, MDA↓, CAT↑ | + | (Jariyawat et al., 2009) |
| In vivo | Acorus calamus L. extracts | *Acorus calamus* L. [Acoraceae] | cisplatin | male Swiss albino mice | 250 mg/kg BW for single dose, ig | 12mg/kg BW for single injection, ip | SrCr↓, BUN↓, GSH↑, GPx↑, SOD↑, CAT↑, | + | (Sandeep and Krishnan Nair, 2010) |
| In vivo | glycyrrhizic acid | *Glycyrrhiza glabra* L. [Fabaceae] | cisplatin | male Swiss albino mice | 75, 150 mg/kg/d for 7d, ig | 7mg/kg BW for single injection, ip | SrCr↓, BUN↓, DNA fragmentation↓, GSH↑, MDA↓, CAT↑, GPx↑, GR↑, QR↑ | + | (Arjumand and Sultana, 2011) |
| In vitro | chrysanthemum indicum extract | *Chrysanthemum indicum* L. [Asteraceae] | cisplatin | HK-2 cells | 0.025–0.2 %v/v for 1h | 50 mM | GSH↑, NAC↑, apoptosis↓ | _ | (Pongjit et al., 2011) |
| In vivo and in vitro | ginsenoside Rg3 | *Panax ginseng* C.A.Mey [Araliaceae] | cisplatin | male  BALB/c mice / LLC-PK1 cells | 5, 10 mg/kg/d for 15d, ig; 10, 20 μM | 2/5 mg/kg/d for 15d, ip; 50 μM | SrCr↓, BUN↓, MDA↓, ROS↓, GPx↑, SOD↑, CAT↑, Nrf2↓, NQO1↓, HO-1↓ | N | (Lee et al., 2012) |
| In vivo | curcumin | *Curcuma aromatica* Salisb. [Zingiberaceae] et al. | cisplatin | male C57BL/6 mice | 100 mg/kg for single injection, i.p | 20 mg/kg for single injection, i.p | SrCr↓, BUN↓, TNF-α↓, MCP-1↓, ICAM-1↓ | + | (Ueki et al., 2013) |
| In vivo | curcumin | *Curcuma aromatica* Salisb. [Zingiberaceae] et al. | cisplatin | male Wistar rats | 200 mg/kg for single dose, ig | 6 mg/kg for single injection, ip | SrCr↓, BUN↓, LPO↓, GSH↑, GST↑, CAT↑,  MnSOD↓ | N | (Waseem et al., 2013) |
| In vivo | hesperidin | *Citrus × aurantium f. deliciosa* (Ten.) M.Hiroe [Rutaceae] et al. | cisplatin | male Wistar  rats | 200 mg/kg/day for 10d, ig | 7.5 mg/kg for single injection, i.p | SrCr↓, BUN↓, MDA↓, ROS↓, TNF-α↓, GR↑, MPO↓, GSH↑, CAT↑, GPx↑, caspase-3↓ | + | (Sahu et al., 2013) |
| In vivo and in vitro | Chungsimyeonja  tang | - | cisplatin | male SD rats/ PK15 cells | 100, 300, 500 mg/kg for 28d, ig | 5 mg/kg for single injection, ip | SrCr↓, BUN↓, MDA↓, GSH↑ | + | (Kim et al., 2014) |
| In vivo | red ginseng | *Panax ginseng* C.A.Mey [Araliaceae] | cisplatin | male SD rats | 100, 300, 500 mg/kg for 28d， ig | 5 mg/kg for single injection, ip | SrCr↓, BUN↓, MDA↓, GSH↑, TNF-α↓, apoptosis↓ | + | (Kim et al., 2014) |
| In vivo | aged garlic extract | *Allium sativum* L. [Amaryllidaceae] | cisplatin | male Wistar  rats | 250 mg/kg/d for 21d, ig | 7.5 mg/kg for single injection, ip | SrCr↓, BUN↓, MDA↓, GSH↑, CAT↑, SOD↑ | + | (Nasr and Saleh, 2014) |
| In vivo | pseudoginsenoside F11 | *Panax quinquefolius* L. [Araliaceae] | cisplatin | male SD rats | 10 mg/kg/d for 9d, ig | 6 mg/kg for single injection, ip | SrCr↓, BUN↓, LPO↓, GPx↑, SOD↑, Bax/Bcl2↓, p53↓, caspase-3/9↓ | + | (Wang et al., 2014) |
| In vivo | zingiber officinale Roscoe | *Zingiber officinale* Roscoe [Zingiberaceae] | cisplatin | adult male albino rats | 120 mg/kg every other day for 4w, ig | 3.3 mg/kg BW for 3d, ip | SrCr↓, BUN↓, Bax↓, Bcl-2↑, | + | (Ali et al., 2015) |
| In vivo and in vitro | Radix Ginseng, ginsenoside Rg3/Rg5/Rk1 | *Panax ginseng* C.A.Mey [Araliaceae] | cisplatin | male C57/BL6 mice/ LLC-PK1 cells | 120 mg/kg/d for 10d, ig; 50, 100, 250 μg/mL | 16 mg/kg for single injection, ip; 25 mM | JNK↓, p53↓, caspase-3↓ | N | (Park et al., 2015) |
| In vivo | curcumin | *Curcuma aromatica* Salisb. [Zingiberaceae] et al. | cisplatin | male Wistar  Rats | 100 mg/kg/d for 10d, ig | 7 mg/kg for single injection, ip | SrCr↓, BUN↓, MDA↓, NAMPT↑, SIRT1↑, SIRT3↑, SIRT4↑ | N | (Ugur et al., 2015) |
| In vitro | Angelica Sinensis Radix | *Angelica sinensis* (Oliv.) Diels [Apiaceae] | cisplatin | HK-2 cells | 5, 50 mg/ml | 10 mM | cell proliferation↑, apoptosis↓, ROS↓ | N | (Bunel et al., 2015) |
| In vitro | ferulic acid | *Angelica sinensis* (Oliv.) Diels [Apiaceae] et al. | cisplatin | HK-2 cells | 1, 10, 50 μM | 10 μM | cell proliferation↑, collagen deposition↓, apoptosis↓, ROS↓ | N | (Bunel et al., 2015) |
| In vivo and in vitro | WZ tablet | *Schisandra chinensis* (Turcz.) Baill. [Schisandraceae] | cisplatin | male NIH mice/ HK-2 cells | 0.5 g/kg/d for 10d, ig; 2, 5, 10, 20 μg/ml | 15 mg/kg for single injection, ip; 30 μM | SrCr↓, BUN↓, MDA↓, ROS↓, GSH↑, LDH↓, SOD↑, Nrf2↑, NQO1↑, HO-1↑, GCL↑ | + | (Jin et al., 2015) |
| In vivo | Panax notoginseng saponins | *Panax notoginseng* (Burkill) F.H.Chen [Araliaceae] | cisplatin | male SD rats | 31.35 mg/kg/d for single dose, ip | 5 mg/kg for single injection, ip | SrCr↓, BUN↓, HIF-1↑, Bcl-2↑, Atg5↑, Beclin-1↑, BNIP3↑, LC3II/LC3I↑ | + | (Liu et al., 2015) |
| In vivo | resveratrol | *Reynoutria japonica* Houtt. [Polygonaceae] et al. | cisplatin | female Swiss albino mice | 25 mg/kg/d for 45d, ip | 5 mg/kg/d for 45d, ip | SrCr↓, BUN↓, GSH↑ | + | (Osman et al., 2015) |
| In vivo and in vitro | trichosanthes kirilowii extract | *Trichosanthes kirilowii* Maxim. [Cucurbitaceae] | cisplatin | rats/ PK15 cells | 100 mg/kg/d for 4w, ig; 50 μg/mL | 5 mg/kg for single injection, ip; 15 μg/mL | SrCr↓, BUN↓, GSH↑, MDA↓, ROS↓, p53↓ | + | (Seo et al., 2015) |
| In vivo and in vitro | curcumin | *Curcuma aromatica* Salisb. [Zingiberaceae] et al. | cisplatin | male Wistar  Rats/ LLC-PK1 cells | 80 mg/kg/d for 10d, ig; 12.5, 25, 50, 100 µM | 7.5 mg/kg BW for single injection, ip;  25 µM | SrCr↓, BUN↓, COX-2↓, caspase-3↓ | + | (Song et al., 2015) |
| In vivo | Radix Ginseng | *Panax ginseng* C.A.Mey [Araliaceae] | cisplatin | male SD rats | 100 mg/kg/d BW for 15d, ig | 7.5 mg/kg BW for single injection, ip | SrCr↓, BUN↓, p53↓, IL-6↓, TNF-α↓, NO↓, TBARS↓, GST↑, GP_X_↑, CAT↑, SOD↑, GSH↑, Na/K ATPase↑ | + | (Yousef and Hussien, 2015) |
| In vivo | aged garlic extract | *Allium sativum* L. [Amaryllidaceae] | cisplatin | male Wistar  rats | 250 mg/kg/d for 21d ig | 7.5 mg/kg for single injection, ip | SrCr↓, BUN↓, SOD↑, CAT↑, MDA↓, GSH↑ | + | (Youssef Nasr and Al Shahat Ibrahim, 2015) |
| In vitro | aqueous extract of cinnamon bark | *Neolitsea cassia* (L.) Kosterm. [Lauraceae] | cisplatin | Vero cells | 10, 20, 40, 50 μg/mL | 10, 15, 20, 25 μg/mL | caspase-3↓, HO-1↑, Cyto c↓, Bax↓, ROS↓ | N | (ElKady and Ramadan, 2016) |
| In vitro | Radix Ginseng, ginsenoside Rg3 | *Panax ginseng* C.A.Mey [Araliaceae] | cisplatin | LLC-PK1 cells | 100, 250, 500, 1000 μg/mL; 25, 50, 125, 250 μg/mL | 25 µM | JNK↓, p53↓, caspase-3↓ | N | (Han et al., 2016) |
| In vivo | eisenia foetida extract | *Pheretimaas pergillum(E Perrier)* | cisplatin | male SD rats | 300, 500 mg/kg/d for 5d, ip and/or ig | 7.5 mg/kg/d for 5d, ip | SrCr↓, BUN↓, MDA↓ | + | (Jamshidzadeh et al., 2016) |
| In vivo | ginsenoside Rg5 | *Panax ginseng* C.A.Mey [Araliaceae] | cisplatin | male ICR mice | 10, 20 mg/kg/d for 10d, ig | 25 mg/kg for single injection, ip | SrCr↓, BUN↓, MDA↓, SOD↑, TNF-α↓, IL-1β↓, COX-2↓, NF-κB↓, Bax↓, Bcl-2↑, CYP2E1↓, 4-HNE↓ | + | (Li et al., 2016) |
| In vivo | curcumin | *Curcuma aromatica* Salisb. [Zingiberaceae] et al. | cisplatin | male Wistar  rats | 200 mg/kg/d for 4d, ig | 7 mg/kg for single injection, ip | SrCr↓, BUN↓, MPO↓, TNF-α↓, Il-1β↓, IL-6↓, IL-10↑, MDA↓, caspase-3↓, Fas↓, FasL↓, p53↓ | + | (Topcu-Tarladacalisir et al., 2016) |
| In vivo | curcumin | *Curcuma aromatica* Salisb. [Zingiberaceae] et al. | cisplatin | male Wistar rats | 200 mg/kg/dose for 3 dose, ig | 5 mg/kg for single injection, ip | SrCr↓, BUN↓, NGAL↓, Kim-1↓, caspase-3↓, TGFβ1↓, collagen I↓, collagen IV↓, α-SMA↓, MDA↓, CAT↑, GR↑, Nrf2↑, Hsp70/72↓, 3-NT↓, PKCβ2↓, claudin-2↑, occludin↑, E-cadherin↑, β-catenina↑ | + | (Trujillo et al., 2016) |
| In vivo | pomegranate rind extract | *Punica granatum* L. [Lythraceae] | cisplatin | Wistar rats | 50, 100, 200 mg/kg/d for 10d, ig | 8 mg/kg for single injection, ip | SrCr↓, BUN↓, SOD↑, MDA↓, GSH↑, TNF-α↓, caspase-3↓, Il-1β↓, IL-6↓ | + | (Karwasra et al., 2016) |
| In vivo | plantago major | *Plantago asiatica* L. [Plantaginaceae] | cisplatin | male Wistar  rats | 300, 600,1200 mg/kg/d for 20d, ig | 7 mg/kg BW for single injection, ip | SrCr↓, BUN↓, MDA↓, CAT↑ | N | (Parhizgar et al., 2016) |
| In vivo | Radix Salviae Miltiorrhizae | *Salvia miltiorrhiza* Bunge [Lamiaceae] | cisplatin | male ICR mice | 3 g/kg/d for 7d, ig | 20 mg/kg for single injection, ip | SrCr↓, BUN↓, GSH↑, MDA↓, Nrf2↑, HO-1↑, NQO1↑ | + | (Cao et al., 2017) |
| In vitro | licorice extract, glycyrrhizic acid | *Glycyrrhiza glabra* L. [Fabaceae] | cisplatin | HK-2 cells | 50, 100, 200, 400 μg/mL; 1, 2, 5, 10 µM | 40 µM | caspase-3↓, PARP↓, p53↓, ROS↓, p21↑ | N | (Ju et al., 2017) |
| In vitro | ginsenosides Rk3 and Rh4 | *Panax ginseng* C.A.Mey [Araliaceae] | cisplatin | male SD rats / LLC-PK1 cells | 2, 6 mg/kg/d for 5d, ig; 1, 5, 10, 25, 50 μg/ mL | 5 mg/kg for single injection, ip; 20μM | SrCr↓, BUN↓, SOD↑, CAT↑, GR↑, ROS↓ | + | (Baek et al., 2017) |
| In vitro | ginsenoside Rh3 | *Panax ginseng* C.A.Mey [Araliaceae] | cisplatin | LLC-PK1 cells | 100 μM | 25 μM | JNK↓, ERK↓, p38↓, caspase-3↓ | N | (Lee and Kang, 2017) |
| In vivo | saponins from the leaves of *Panax quiquefolium L.* | *Panax quinquefolius* L. [Araliaceae] | cisplatin | male ICR mice | 150, 300 mg/kg/d for 10d, ig | 20 mg/kg for single injection, ip | SrCr↓, BUN↓, SOD↑, MDA↓, GSH↑, TNF-α↓, IL-1β↓, caspase-3/9↓, NF-κB↓, CYP2E1↓, HO-1↓, NOX4↓, COX-2↓, iNOS↓, Bax↓, Bcl-2↑ | + | (Ma et al., 2017) |
| In vivo | Zhen-Wu-Tang | - | cisplatin | male Wistar rats | 1 ml/100g/d for 4d or 10d, ig | 6 mg/kg for single injection, ip | SrCr↓, BUN↓, TGF-β↓, Wnt↓, β-catenin↓, Nrf2↑, PI3K↑, Akt↑, Bax↓, caspase-3↓, α-SMA↓ | + | (Liu et al., 2017) |
| In vivo | genipin | *Gardenia jasminoides* J.Ellis [Rubiaceae] | cisplatin | male C57BL/6J  mice | 1-10 mg/kg for single dose, ip | 20 mg/kg for single injection, ip | SrCr↓, BUN↓, cystatin C↓, Kim-1↓, SOD↑, GSH↑, NADPH oxidase↓, 4-HNE↓, 3-NY↓, TNF-α↓, IL-1β↓, caspase-3/7↓, NF-κB↓, p38↓, JNK↓, PARP↓, DNA fragmentation↓ | + | (Mahgoub et al., 2017) |
| In vivo | curcumin | *Curcuma aromatica* Salisb. [Zingiberaceae] et al. | cisplatin | male Wistar rats | 200 mg/kg/d for 3d, ig | 5 mg/kg for single injection, ip | SrCr↓, BUN↓,  FIS1↓, OPA1↑, parkin↓, SIRT3↑, PINK1↓, Mitochondrial membrane potential↑ | + | (Ortega-Domínguez et al., 2017) |
| In vivo | *Panax quiquefolium L.* berry extract | *Panax quinquefolius* L. [Araliaceae] | cisplatin | male ICR mice | 150, 300 mg/kg/d for 10d, ig | 20 mg/kg for single injection, ip | SrCr↓, BUN↓, MDA↓, CYP2E1↓, 4-HNE↓, GSH↑, SOD↑, TNF-α↓, IL-1β↓, COX-2↓, iNOS↓, Bax↓, Bcl-2↑, caspase-3↓, NF-κB↓, p38↓, JNK↓, ERK↓ | + | (Ma et al., 2017) |
| In vivo and in vitro | Dendrobii Caulis extract | *Dendrobium nobile* Lindl. [Orchidaceae] | cisplatin | male SD rats/ PK15 cells | 100, 300, 500 mg/kg/d for 28d, ig;  50 μg/mL | 5 mg/kg for single injection, ip; 15 μg/mL | SrCr↓, BUN↓, p53↓, GSH↑, ROS↓, MDA↓ | + | (Shin et al., 2017) |
| In vivo | galangin | *Alpinia officinarum* Hance [Zingiberaceae] | cisplatin | male Wistar rats | 25, 50, 100 mg/kg/d for 10d, ig | 8mg/kg for single injection, ip | SrCr↓, BUN↓, SOD↑, MDA↓, GSH↑, TNF-α↓, IL-6↓, Bax↓, Bcl-2↑,  caspase-3↓, DNA fragmentation↓, NF-κB↓, p38↓, JNK↓, ERK↓ | + | (Tomar et al., 2017) |
| In vivo | fruits of Radix Ginseng | *Panax ginseng* C.A.Mey [Araliaceae] | cisplatin | male ICR mice | 200, 400 mg/kg/d for 10d, ig | 25 mg/kg for single injection, ip | SrCr↓, BUN↓, MDA↓, GSH↑, SOD↑, CAT↑, CYP2E1↓, 4-HNE↓, TNF-α↓, IL-1β↓, COX-2↓, iNOS↓, Bax↓, Bcl-2↑ | + | (Qi et al., 2017) |
| In vivo | curcumin | *Curcuma aromatica* Salisb. [Zingiberaceae] et al. | cisplatin | female Wistar rats | 200 mg/kg/d for 10d, ig | 7.5 mg/kg for single injection, ip | SrCr↓, BUN↓, TNF-α↓, IL-1β↓, IL-6↓, IL-8↓, IL-10↑ | + | (Kumar et al., 2017) |
| In vivo and in vitro | dioscin | *Dioscorea oppositifolia* L. [Dioscoreaceae] | cisplatin | male Wistar rats, male C57BL/6J mice/ NRK-52E, HK-2 cells | 10, 20, 40 mg/kg/d for 12d, 15, 30, 60 mg/kg/d for 10d, ig; 0, 50, 100, 200 400, 800, 1600, 3200 ng/mL | 7 mg/kg(rats) and 25 mg/kg (mice) for single injection, ip; 1-12 μg/mL | SrCr↓, BUN↓, GCLC↑, GCLM↑, HO-1↑, Keap1↓, Nrf2↑, AP-1↓, COX-2↓, HMGB1↓, IκB-α↑, IL-1β↓, IL-6↓, TNF-α↓, NF-κB↓, miR-34a↓, Sirt1↑, ROS↓, GSH↑, MDA↓, GPx↑ | + | (Zhang et al., 2017) |
| In vivo | andrographis paniculata | *Andrographis paniculata* (Burm.f.) Nees [Acanthaceae] | cisplatin | male Wistar rats | 200, 400 mg/kg/d for 7d, ig | 10 mg/kg/d for 2d, ip | SrCr↓, BUN↓, Kim-1↓, Nrf2↑, MDA↓, NO↓, MPO↓, GSH↑, SOD↑, GPx↑ | + | (Adeoye et al., 2018) |
| In vivo and in vitro | Huaiqihuang extractum | - | cisplatin | male C57BL/6 mice/ TECs | 6 g/kg/d for 3d, ig; 0/6/12/18 mg/ml | 20mg/kg for single injection, ip; 75μM | SrCr↓, BUN↓, Kim-1↓, HMGB1↓, TLR4↓, p65↓, NF-κB↓, TNF-α↓, Bax↓, Bcl-2↑, RIP3↓, MLKL↓ | + | (Guo et al., 2018) |
| In vitro | butyl pyroglutamate | *Morus alba* L. [Moraceae] | cisplatin | LLC-PK1 cells | 0, 10, 25 μM | 25 μM | JNK↓, p38↓, caspase-3↓ | N | (Lee et al., 2018) |
| In vivo | ginsenoside Re | *Panax ginseng* C.A.Mey [Araliaceae] | cisplatin | male ICR mice | 25 mg/kg/d for 10d, ig | 25 mg/kg for single injection, ip | SrCr↓, BUN↓, MDA↓, GSH↑, SOD↑, CAT↑, CYP2E1↓, 4-HNE↓, TNF-α↓, IL-1β↓, COX-2↓, iNOS↓, Bax↓, Bcl-2↑ | N | (Wang et al., 2018) |
| In vivo | wogonin | *Scutellaria baicalensis* Georgi [Lamiaceae] et al. | cisplatin | male C57BL/6 mice | 12.5, 25, 50 mg/kg/d for 3d, ip | 20 mg/kg for single injection, ip | SrCr↓, BUN↓, Kim-1↓,  NF-κB↓, caspase-3/8↓, RIP3↓, MLKL↓, TNF-α↓, IL-6↓, p65↓ | + | (Meng et al., 2018) |
| In vivo | asiatic acid | *Centella asiatica* (L.) Urban [Apiaceae] et al. | cisplatin | male C57BL/6 mice | 50, 100 mg/kg for single injection, ip | 20 mg/kg for single injection, ip | SrCr↓, BUN↓, Kim-1↓, IL-1β↓, TNF-α↓, MCP-1↓, caspase-1↓, Smad7↑, NF-κB↓, IκBα↑ | + | (Yang et al., 2018) |
| In vivo | tetramethylpyrazine | *Conioselinum anthriscoides 'Chuanxiong'* [Apiaceae] et al. | cisplatin | male SD rats | 50, 100 mg/kg/d for 2w, ig | 7 mg/kg for single injection, ip | SrCr↓, BUN↓, HMGB1↓, TLR4↓, NF-κB↓, HO-1↑, TNF-α↓, IL-1β↓, GSH↑, SOD↑, PPAR-γ↑, Nrf2↑, Bax↓, Bcl2↑, COX-2↓, caspase-3↓, NQO1↑, iNOS↓, Kim-1↓ | + | (Michel and Menze, 2019) |
| In vivo and in vitro | anemoside B4 | *Pulsatilla chinensis* (Bunge) Regel [Ranunculaceae] | cisplatin | ICR mice/ HEK 293 cells | 50, 100 mg/kg/d for 3d, ip; 3-12 μM | 20 mg/kg for single injection, ip; 20 μM | SrCr↓, BUN↓, GSH↑, CAT↑, ROS↓, SOD↑, PARP↓ | N | (He et al., 2019) |
| In vivo and in vitro | ginsenoside Rb3 | *Panax ginseng* C.A.Mey [Araliaceae] | cisplatin | ICR mice/ HEK 293 cells | 10, 20 mg/kg/d for 10d, ig; 0.25, 0.5, 1, 2 μM | 25 mg/kg for single injection, ip; 20 μM | SrCr↓, BUN↓, GSH↑, SOD↑, MDA↓, p62↓, Atg3↓, Atg5↓, Atg7↓, BNIP3↓, P-AMPK/AMPK↓, p-mTOR/mTOR↑, caspase-3/9↓, Bax↓, Bad↓, Bcl-2↑, Bcl-XL↑ | + | (Xing et al., 2019) |
| In vivo | curcumin | *Curcuma aromatica* Salisb. [Zingiberaceae] et al. | cisplatin | male SD rats | 100 mg/kg/d for 9d, ig | 7 mg/kg for single injection, ip | SrCr↓, BUN↓, TNF-α↓, IL-6↓, IL-1β↓, KIM-1↓, NGAL↓, Bax↓, Bcl-2↑, NF-κB↓, OCT2↓, Ctr1↓, MDA↓, GSH↑ | + | (Soetikno et al., 2019) |
| In vivo | Scutellariae Radix | *Scutellaria baicalensis* Georgi [Lamiaceae] | cisplatin | male C57BL/6J mice | 300 mg/kg/d for 16d, ig | 3 mg/kg/d for 14d, ip | SrCr↓, BUN↓, apoptosis↓ | N | (Huang et al., 2019) |
| In vivo | terminalia chebula extract | *Terminalia chebula* Retz. [Combretaceae] | cisplatin | male Wister rats | 50, 100, 200 mg/kg/d for 10d, ig | 8 mg/kg for single injection, ip | SrCr↓, BUN↓, GSH↑, SOD↑, MDA↓, TNF-α↓, IL-1↓, IL-6↓, caspase-3↓, Bax↓, Bcl-2↑ | + | (Kalra et al., 2019) |
| In vivo | zingerone | *Zingiber officinale* Roscoe [Zingiberaceae] et al. | cisplatin | female Wistar rats | 25, 50 mg/kg/d for 7d, ig | 7 mg/kg for single injection, ip | SrCr↓, BUN↓, SOD↑, CAT↑, GPx↑, GSH↑, AQP1↑, TNF-α↓, IL-1↓, IL-6↓, IL-33↓, NF-κB↓, iNOS↓, COX-2↓, p53↓, caspase-3↓, Bax↓, Bcl-2↑ | + | (Kandemir et al., 2019) |
| In vivo | arginyl-fructosyl-glucose | *Panax ginseng* C.A.Mey [Araliaceae] | cisplatin | male ICR mice | 40, 80 mg/kg/d for 10d, ig | 20 mg/kg for single injection, ip | SrCr↓, BUN↓, SOD↑, CAT↑, MDA↓, NF-κB↓, IκBα↓, TNF-α↓, IL-1β↓, IL-6↓, iNOS↓, COX-2↓, PI3K↑, Akt↑, caspase-3↓, Bax↓, Bcl-2↑ | + | (Li et al., 2019) |
| In vivo | ginsenoside Rh2 | *Panax ginseng* C.A.Mey [Araliaceae] | cisplatin | male ICR mice | 20, 40 mg/kg/d for 10d, ig | 20 mg/kg for single injection, ip | SrCr↓, BUN↓, Cyto c↓, p53↓, caspase-3/8/9↓, Bax↓, Bcl-2↑, iNOS↓, COX-2↓, TNF-α↓, NF-κB↓ | + | (Qi et al., 2019) |
| In vivo and in vitro | curcumin | *Curcuma aromatica* Salisb. [Zingiberaceae] et al. | cisplatin | male C57BL/6 mice/ RAW264.7 cells | 100mg/kg/d for 3d, ip | single injection, ip | SrCr↓, BUN↓, IL-1β↓, IL-6↓, TNF-α↓, MCP-1↓, iNOS↓, Syk↓, NF-κB↓, Mincle↓ | N | (Tan et al., 2019) |
| In vivo | propolis | *Colla Apis* | cisplatin | male SD rats | 50,100 mg/kg/d for 14d, ig | 7 mg/kg for single injection, ip | SrCr↓, BUN↓, TOS↑, TAS↓, OSI↓, MDA↓, | + | (Yuluğ et al., 2019) |
| In vitro | icariin | *Epimedium brevicornu* Maxim. [Berberidaceae] | cisplatin | HEK-293 cells | 0.25, 0.5, 1, 2 μM | 20 μM | MDA↓, ROS↓, GSH↑, NF-κB↓, IL-1β↓, TNF-α↓, iNOS↓, Bax↓, Bcl-2↑, caspase-3/9↓, PI3K↑, Akt↑ | N | (Zhou et al., 2019) |
| In vivo | sesamin | *Sesamum indicum* L. [Pedaliaceae] | cisplatin | male Wistar rats | 5 mg/kg/d for 10d, ig | 5 mg/kg for single injection, ip | SrCr↓, BUN↓, NGAL↓, Kim-1↓, IL-1β↓, TNF-α↓, TGFβ-1↓, cystatin C↓, MPO↓, SOD↑, CAT↑, GR↓, TAC↓, MDA↓, Nrf2↓ | + | (Ali et al., 2020) |
| In vivo | Astragalus propinquus Schischkin and Panax notoginseng | *Astragalus mongholicus* Bunge [Fabaceae]; *Panax notoginseng* (Burkill) F.H.Chen [Araliaceae] | cisplatin | male C57BL/6 mice | 1972, 3943 mg/kg/day or 3d, ig | 20 mg/kg for single injection, ip | SrCr↓, BUN↓, IL-1β↓, IL-6↓, TNFα↓, MCP-1↓, NF-κB↓, iNOS↓, Mincle↓ | N | (Hui et al., 2020) |
| In vitro | Inchenhao-tang, Liuweidihuang-tang, Longdanxiegan-tang | - | cisplatin | NRK-52E cells and HK-2 cells | 1.2 mg/mL | 50 µm | SOD↑, p38↓, JNK↓, caspase-3↓ | N | (Dachuri et al., 2020) |
| In vitro | ginsenoside Rk1 | *Panax ginseng* C.A.Mey [Araliaceae] | cisplatin | HEK-293 cells | 5, 10, 20, 30, 50, 75, 100, 125 μM | 5, 10, 20, 40, 80 μM | GSH↑, MDA↓, Nrf2↑, HO-1↑, caspase-3/9↓, Bax↓, Bcl-2↑ | N | (Hu et al., 2020) |
| In vivo | ganoderma lucidum | *Ganoderma lucidum* (Curtis) P. Karst. [Ganodermataceae] | cisplatin | male SD rats | 500 mg/kg/d for 10d, ig | 12 mg/kg for single injection, ip | SrCr↓, BUN↓, SOD↑, HMGB-1↓, NF-κB↓, caspase-3↓, LC3 II↑, TDT↓, EGFR↓ | + | (Mahran and Hassan, 2020) |
| In vivo and in vitro | dendropanoxide | *Campanula latifolia* L. [Campanulaceae] | cisplatin | male SD rats/ NRK-52E cells | 5, 10 mg/kg/d for 7d, ig; 5, 10 μg/mL | 6 mg/kg for single injection, ip; 20 μM | SrCr↓, BUN↓, Kim-1↓, SBP-1↓, NGAL↓, Bax↓, Bcl-2↑, p53↓, SOD↑, GSH↑, MDA↓, CAT↑, TNF-α↓, IL-6↓, IL-1β↓, IL-10↑, Akt↓, AMPK↓, mTOR↑ | + | (Park et al., 2020) |
| In vivo and in vitro | 7-Hydroxycoumarin | *Eucommia ulmoides* Oliv. [Eucommiaceae] et al. | cisplatin | male C57BL/6 mice/ HK2 cells | 30, 60, 90 mg/kg/d for 3d; ip; 0-80 µM | 20 mg/kg for single injection, ip; 20 µM | SrCr↓, BUN↓, Kim-1↓, RIPK1↓, RIPK3↓, MLKL↓, TNF-α↓, MCP-1↓, p65↓, IL-1β↓, caspase-3↓, cyclin D1↑, Sox9↑ | + | (Wu et al., 2020) |
| In vivo | Panax notoginseng saponins | *Panax notoginseng* (Burkill) F.H.Chen [Araliaceae] | cisplatin | male SD rats | 31.35 mg/kg/d for 3d; ip | 5 mg/kg for single injection, ip | SrCr↓, BUN↓, ROS↓, MDA↓, NO↓, SOD↑, GSH↑, CAT↑, ATP↑, MMP↑, MPTP↓, | N | (Li et al., 2020) |
| In vivo | pulchinenoside B4 | *Pulsatilla chinensis* (Bunge) Regel [Ranunculaceae] | cisplatin | male ICR mice | 150, 300 mg/kg/d for 10d, ig | 20 mg/kg for single injection, ip | SrCr↓, BUN↓, TNF-α↓, IL-1β↓, iNOS↓, COX-2↓, Bax↓, Bcl-2↑, caspase 3/9↓, IκKα↓, IκBα↓, NF-κB↓, p38↓, JNK↓, ERK↓ | + | (Wang et al., 2020) |
| In vivo | curcumin | *Curcuma aromatica* Salisb. [Zingiberaceae] et al. | cisplatin | female albino rats | 200 mg/kg/d for 7d, ig | 7.5 mg/kg for single injection, ip | SrCr↓, BUN↓, iNOS↓, GSH↑, MDA↓, Bax↓, TNF-α↓, caspases 3↓ | N | (Abd El-Kader and Taha, 2020) |
| In vivo | curcumin | *Curcuma aromatica* Salisb. [Zingiberaceae] et al. | cisplatin | male C57BL/6 mice | 20 mg/kg/d for 6d, ip | 20 mg/kg for single injection, ip | BUN↓, miR-181a↓, PTEN↑ | + | (Huang et al., 2020) |
| In vivo | loganin | *Strychnos nux-vomica* L. [Loganiaceae] | cisplatin | male C57BL/6 mice | 1, 10, 20 mg/kg for single dose, ig | 10 mg/kg for single injection, ip | SrCr↓, BUN↓, IL-1β↓, IL-6↓, TNF-α↓, ERK↓ | + | (Kim et al., 2021) |
| In vitro | panax notoginseng saponins | *Panax notoginseng* (Burkill) F.H.Chen [Araliaceae] | cisplatin | HK-2 cells | 6.25 mg/mL | 6.25 μg/mL | HIF-1α↑, BNIP3↑,  Cyto c↓, Bcl2↑, Bax↓,  caspases 3↓ | N | (Li et al., 2021) |
| In vivo | ginseng polysaccharide | *Panax ginseng* C.A.Mey [Araliaceae] | cisplatin | male ICR mice | 100, 200 mg/kg/d for 10d, ig | 20 mg/kg for single injection, ip | SrCr↓, BUN↓, eIF2α↓, ATF4↓, CHOP↓, NF-κB↓, TNF-α↓, caspases 3↓, PI3K↑, AKT↑, PERK↓, Bax↓, Bcl-2↑ | + | (Wei et al., 2021) |
| In vivo and in vitro | gypenoside XLIX | *Gynostemma pentaphyllum* (Thunb.) Makino [Cucurbitaceae] | cisplatin | male C57BL/6 mice/ HK-2 cells | 25, 50, 100 mg/kg/d for 3d, ip; 0.5-256 μg/ml | 20 mg/kg for single injection, ip; 20 μM | SrCr↓, BUN↓, Kim-1↓, IL-6↓, TNF-α↓, MCP-1↓, P65↓, RIPK1↓, RIPK3↓, MLKL↓, IGFBP7↓, IGF1R↑, caspases 3↓ | + | (Yang et al., 2021) |
| In vivo and in vitro | ginsenoside Rb3 | *Panax ginseng* C.A.Mey [Araliaceae] | cisplatin | male nude mice/ GP-293 cells | 10, 20 mg/kg/d for 28d, ig; 1, 2, 5 μM | 15 mg/kg/d every other day for 28d, ip; | SrCr↓, BUN↓, TGF-β↓, Smad2↓, Smad3↓, Bcl2↑, Bax↓, caspases 3/8/9↓, PARP↓ | N | (Wu et al., 2021) |
| In vivo and in vitro | ginsenoside Rg3 | *Panax ginseng* C.A.Mey [Araliaceae] | cisplatin | male  Kunming mice/ HK-2 cells | 5 mg/kg/d for 10d, ig; 20, 40, 80 μg/ml | 4 mg/kg/d every other day for 10d, ip; 10 μM | LC3II/I↑, Beclin-1↑, p62↓, NLRP3↓, ASC↓, caspase-1↓, IL-1β↓ | + | (Zhai et al., 2021) |
| In vivo | curcumin | *Curcuma aromatica* Salisb. [Zingiberaceae] et al. | doxorubicin | male Wistar rats | 200 mg/kg/d for 30d, ig | 7.5 mg/kg for single injection, ip | SrCr↓, BUN↓, MDA↓, GPx↑, mitochondrial lipid peroxidation ↓ | N | (Venkatesan et al., 2000) |
| In vitro | tetramethylpyrazine | *Conioselinum anthriscoides 'Chuanxiong'* [Apiaceae] et al. | doxorubicin | NRK-52E cells | 10, 100 μM | 4 μM | ROS↓, caspases 3/8/9↓, Bcl-xL↑, Cyto c↓, NF-κB↓, TNF-α↓ | N | (Cheng et al., 2006) |
| In vivo | ginger | *Zingiber officinale* Roscoe [Zingiberaceae] | doxorubicin | female SD rats | 200, 400 mg/kg for single dose, ig | 15 mg/kg for single injection, ip | SOD↑, CAT↑, GPx↑, GSH↑, GST↑, MDA↓ | N | (Ajith et al., 2008) |
| In vivo | curcumin | *Curcuma aromatica* Salisb. [Zingiberaceae] et al. | doxorubicin | male Wistar rats | 250 mg/kg for 56d, ig | 3.5 mg/kg for single injection, iv | SrCr↓, MCP-1↓, TGF-β1↓ | + | (Russo et al., 2018) |
| In vitro | wedelolactone | *Eclipta prostrata* (L.) L. [Asteraceae] | doxorubicin | MPC-5 cells | 1.25, 5, 20 μM | 4 μM | SOD↑, CAT↑, GPx↑, MDA↓, IL-6↓, MCP-1↓, TNF-α↓, TGF-β1↓, IκKα↓, IκBα↓, NF-κB↓ | N | (Zhu et al., 2019) |
| In vivo | Bu-zhong-yi-qi decoction | - | 5-fluorouracil | Kunming mice | 1, 2 g raw herb/kg/d for 10d, ig | 100 mg/kg/d for 6d, ip | SrCr↓, BUN↓, SOD↑, CAT↑, GPx↑, caspase-3↓ | + | (Xiong et al., 2016) |
| In vivo | garlic aqueous extract | *Allium sativum* L. [Amaryllidaceae] | methotrexate | male Wistar rats | 1 mL/100g BW for 7d, ig | 20 mg/kg for single injection, ip | SrCr↓, BUN↓, MDA↓, GSH↑, NO↓, | + | (Ahmed et al., 2015) |
| In vivo | Rhizoma Curculiginis | *Curculigo orchioides* Gaertn. [Hypoxidaceae] | cyclophosphamide | male Swiss albino mice | 1.5 mmol/kg BW for 5d, ip | 20 mg/kg BW for single injection, ip | SrCr↓, BUN↓, IFN-γ↓, TNF-α↓, IL-2↓, GSH↑, GPx↑ | + | (Murali and Kuttan, 2016) |
| In vivo | propolis | *Colla Apis* | methotrexate | male Wistar rats | 100 mg/kg/d for 15d, ig | 20 mg/kg for single injection, ip | HSP-70↓, apoptosis↓ | + | (Ulusoy et al., 2016) |
| In vivo | mistletoe extract | *Viscum coloratum* (Kom.) Nakai [Santalaceae] | methotrexate | female Wistar rats | 5 mg/kg/d BW for 10d, ip | 10 mg/kg/d BW for 3d, ip | SrCr↓, BUN↓, GSH↑, SOD↑, MPO↓ | + | (Sakalli Çetin et al., 2017) |
| In vivo | iridoid glycosides fraction | *Scrophularia ningpoensis* Hemsl. [Scrophulariaceae] et al. | cyclophosphamide | Male Swiss albino mice | 25, 50, 100 mg/kg/d for 21d, ig | 200 mg/kg/d for 2d, ip | SrCr↓, BUN↓, IL-1β↓, TNF-α↓, NF-κB↓, Bax↓, Bcl-2↑, caspase 3/9↓, PPAR-γ↑ | + | (Sharma et al., 2017) |
| In vivo | chicoric acid | *Matricaria chamomilla* L. [Asteraceae] | methotrexate | male Wistar rats | 25, 50 mg/kg/d for 19d, ig | 20 mg/kg for single injection, ip | SrCr↓, BUN↓, Bax↓, Bcl-2↑, Nrf2↑, NQO1↑, HO-1↑, Kim-1↓, IL-1β↓, caspase 1/3↓, NF-κB↓, NLRP3↓, MDA↓, ROS↓, NO↓, GSH↑, SOD↑, CAT↑, GPx↑ | + | (Abd El-Twab et al., 2019) |
| In vivo | Wuzhi capsule | *Schisandra chinensis* (Turcz.) Baill. [Schisandraceae] | methotrexate | male SD rats | 300 mg/kg/d for 3d or 7d, ig | 300 mg/kg for single injection, ip | SrCr↓, BUN↓, MDA↓, GSH↑, SOD↑, GPx↑ | + | (Chen et al., 2019) |
| In vivo | Folium Ginkgo extract | *Ginkgo biloba* L. [Ginkgoaceae] | methotrexate | male SD rats | 60 mg/kg for 10d, ig | 20 mg/kg for single injection, ip | SrCr↓, BUN↓, TGF-β↓, PI3K↓, Akt↓, mTOR↓ | + | (Sherif et al., 2019) |
| In vivo | amomum subulatum fruits | *Elettaria cardamomum* (L.) Maton [Zingiberaceae] | methotrexate | male BALB/c mice | 250 mg/kg/d for 10d, ig | 20 mg/kg for single injection, ip | SrCr↓, BUN↓, TNF-α↓, IL-1β↓, IL-6↓, SOD↑, GPx↑, GSH↑, CAT↑, MDA↓, NO↓, | + | (Drishya et al., 2021) |
| In vivo | rosmarinic acid | *Perilla frutescens* (L.) Britton [Lamiaceae] et al. | methotrexate | Wistar rats | 100,200 mg/kg/d for 12d, ip | 20 mg/kg/d for 3d, ip | SrCr↓, BUN↓, MDA↓, GSH↑, CAT↑ | + | (Jafaripour et al., 2021) |
| In vivo and in vitro | dioscin | *Dioscorea oppositifolia* L. [Dioscoreaceae] | methotrexate | male SD rats/ L-02 and NRK-52E cells | 15, 30, 60 mg/kg/d for 7d, ig; 25, 50, 100, 200, 400 nmol/L | 20 mg/kg for single injection, ip;  5 μM | SrCr↓, BUN↓, SOD↑, MDA↓, GSH↑, GPx↑, Nrf2↑, Keap1↓, HO-1↑, GCLC↑, NQO1↑ | N | (Li et al., 2021) |
| In vivo | Huaiqihuang granule | - | cyclophosphamide | male SD rats | 3, 6 mg/kg/d for 5d, ig | 200 mg/kg for single injection, ip | SrCr↓, BUN↓, SOD↑, MDA↓, CAT↑, NLRP3↓, caspase 3/9↓, NF-κB↓, Bax↓, Bcl-2↑, TNF-α↓, IL-1β↓, IL-6↓, p38↓, JNK↓, ERK↓ | + | (Zhang et al., 2021) |

**Table 2 Antimicrobial induced DI-AKI**

| **Type** | **Herbal/ Active ingredients/ Formula** | **Species, Source** | **Drug** | **Animal/Cell** | **Dose, Duration, Route of H/A/F** | **Dose, Duration, Route of drug** | **Main outcome** | **Histopathology**  **Score^a^** | **Study** |
| --- | --- | --- | --- | --- | --- | --- | --- | --- | --- |
| In vivo and in vitro | cordyceps sinensis | *Ophiocordyceps sinensis* (Berk.) G.H. Sung, J.M.Sung, Hywel-Jones & Spatafora [Clavicipitaceae] | gentamicin | male SD rats/ renal tubular cells | 0.5 g/kg/d for 7d, ig | 160 mg/kg/d for 7d, ip | SrCr↓, BUN↓, NAG↓, LDH↓, Na/K ATPase↑, MDA↓, SOD↑, tissue calcium↓, c-myc↑, EGF↑ | N | (Li et al., 1996) |
| In vivo | Folium Ginkgo extract | *Ginkgo biloba* L. [Ginkgoaceae] | gentamicin | male Wistar rats | 300 mg/kg/d for 10d, ig | 80 mg/kg/d for 8d, ip | SrCr↓, BUN↓, MDA↓ | + | (Naidu et al., 2000) |
| In vivo | Folium Ginkgo extract | *Ginkgo biloba* L. [Ginkgoaceae] | gentamicin | male SD rats | 100 mg/kg for 35d, ig | 5 mg/kg for single injection, iv | SrCr↓, BUN↓, GSH↑, NO↓, NAG↓, SOD↑ | N | (Abd-Ellah and Mariee, 2007) |
| In vitro and in vivo | tetramethylpyrazine | *Conioselinum anthriscoides 'Chuanxiong'* [Apiaceae] et al. | gentamicin | male SD rats/ NRK-52E | 80 mg/kg/d for 7d, ip; 50, 100 μM | 20 mg/kg/d for 7d, ip;  3 mM | Bcl-xL↑, TNF-α↓, NF-κB↓, caspase-3/8/9↓, Cyto c↓ | N | (Juan et al., 2007) |
| In vivo | VI-28 | - | gentamicin | male SD rats | 80, 240 mg/kg/d for 12d, ig | 100 mg/kg/d for 8d, ip | SrCr↓, BUN↓, GSH↑, SOD↑, GPx↑, GST↑ | N | (Poon et al., 2008) |
| In vivo and in vitro | tetramethylpyrazine | *Conioselinum anthriscoides 'Chuanxiong'* [Apiaceae] et al. | gentamicin | C57B6 mice/ NRK-52E | 80 mg/kg/d for 7d, ip; 0, 50, 100 mM | 20 mg/kg/d for 7d, ip; 3 mM | SrCr↓, BUN↓, HO-1↑, Bcl-xL↑, Hax-1↑, NADPH↓, NF-κB↓, COX-2↓, caspases-3/9↓ | + | (Sue et al., 2009) |
| In vivo | crocus sativus | *Carthamus tinctorius* L*.* [Asteraceae] | gentamicin | male Wistar rats | 40, 80 mg/kg/d for 10d, ig | 80 mg/kg/d for 5d, ip | SrCr↓, BUN↓, MDA↓ | + | (Ajami et al., 2010) |
| In vivo | sesame oil | *Sesamum indicum* L. [Pedaliaceae] | gentamicin | male SD rats | 1, 2, 4 ml/kg for single dose, ig | 100 mg/kg/day for 7d, sc | SrCr↓, BUN↓, MDA↓, NO↓, osteopontin↓, superoxide anion↓, peroxynitrite radical↓ | + | (Periasamy et al., 2010) |
| In vivo | curcumin | *Curcuma aromatica* Salisb. [Zingiberaceae] et al. | gentamicin | male Wistar rats | 200 mg/kg/d for 7, 15, 30d, ig | 100 mg/kg/d for 6d, ip | SrCr↓, BUN↓, LPO↓, SOD↑, GPx↑, GSH↑, GST↑, iNOS↓, NF-κB↓ | N | (Manikandan et al., 2011) |
| In vivo | sesame oil | *Sesamum indicum* L. [Pedaliaceae] | gentamicin+ iodinated contrast | male SD rats | 0.5 ml/kg for single dose, ig | 100 mg/kg BW for 5d, sc | SrCr↓, BUN↓, MDA↓, superoxide anion↓, MPO↓, iNOS↓ | + | (Hsu et al., 2011) |
| In vivo | Zingiber officinale and Curcuma longa | *Zingiber officinale* Roscoe [Zingiberaceae]; *Curcuma longa* L. [Zingiberaceae] | gentamicin | male Wistar rats | 2%, 4% for 30d, ig | 100 mg/kg BW for 3d, ip | SrCr↓, BUN↓, MDA↓, GSH↑, SOD↑, GST↑ | N | (Ademiluyi et al., 2012) |
| In vivo | licorice | *Glycyrrhiza glabra* L. [Fabaceae] | gentamicin | Wistar rats | 1kg licorice water extract for 12d, ig | 100 mg/kg/d for 12d, ip | SrCr↓, BUN↓, MDA↓, GSH↑, TAC↑ | + | (Aksoy et al., 2012) |
| In vivo | Radix Ginseng | *Panax ginseng* C.A.Mey [Araliaceae] | gentamicin | male SD rats | 100, 200 mg/kg/d for 10d, ip | 100 mg/kg/d for 10d, ip | SrCr↓, BUN↓, Bax↓, Bcl-2↑ | + | (Kalkan et al., 2012) |
| In vivo and in vitro | Zhibai Dihuang Wan | - | gentamicin | male BALB/c mice/ NRK-52E cells | 1, 2 g/kg/d for 10d, ig; 30 μg/ml | 20 mg/kg/d for 10d, ip; 3mM | SrCr↓, BUN↓, Bcl-2↑, caspase-3↓ | N | (Hsu et al., 2014) |
| In vivo and in vitro | red ginseng extract | *Panax ginseng* C.A.Mey [Araliaceae] | gentamicin | male SD rats/ NRK-52E cells | 100mg/kg/d for 40d, ig; 100 mg/ml | 100mg/kg/d for 10d, ip; 3 mM | SrCr↓, BUN↓, GSH↑,  caspase-9↓, caspase-3↓, Bax↓, Bcl-2↑, Cyto c↓ | + | (Shin et al., 2014) |
| In vivo | curcumin | *Curcuma aromatica* Salisb. [Zingiberaceae] et al. | gentamicin | male SD rats | 100 mg/kg/d for 8d, ig | 80 mg/kg/d for 8d, ip | SrCr↓, BUN↓, NGAL↓, Kim-1↓, Bcl-2↑, caspase-3↓, SOD↑, CAT↑, GSH↑, MDA↓, Nrf2↑, SIRT1↑, HO-1↑ | N | (He et al., 2015) |
| In vivo and in vitro | curcumin | *Curcuma aromatica* Salisb. [Zingiberaceae] et al. | gentamicin | male Wistar rats / LLC-PK1 cells | 400 mg/kg/d for 12d, ig; 10, 20, 30 μM | 75 mg/kg/12h for 7d, sc; 8mM GM | SrCr↓, BUN↓, Nrf2↑, PGC-1α↑, Complex I↑, Complex II↑, Complex III↑, Complex IV↑, MPT↓ | N | (Negrette-Guzmán et al., 2015) |
| In vivo | propolis | *Colla Apis* | gentamicin | Swiss albino male mice | 500 mg/kg BW for 7d, ig | 80 mg/kg BW for 7d, ig | SrCr↓, BUN↓, Kim-1↓, MDA↓, caspase-3↓ | + | (Aldahmash et al., 2016) |
| In vivo | leaf extract of Eclipta alba | *Eclipta prostrata* (L.) L. [Asteraceae] | gentamicin | female SD rats | 300, 600 mg/kg BW for 7d, ig | 80mg/kg/d for 7d, sc | SrCr↓, BUN↓, CAT↑, MDA↓ | + | (Dungca, 2016) |
| In vivo | rhizoma smilacis glabrae | *Smilax glabra* Roxb*.* [Smilacaceae] | gentamicin | male SD rats | 0.75, 1.5, 3g/kg for single dose, ig | 100 mg/kg for single injection, im | SrCr↓, BUN↓, SOD↑, CAT↑, GSH↑, caspase-3↓ | + | (Liu et al., 2017) |
| In vivo | ferulic acid | *Angelica sinensis* (Oliv.) Diels [Apiaceae] et al. | gentamicin | male Albino rats | 100 mg/kg/d for 19d, ig | 40 mg/kg/d for 9d, ip | SrCr↓, BUN↓, NAG↓, CAT↑, PPAR-γ↑ | + | (El-Ashmawy et al., 2018) |
| In vivo | aqueous garlic extract | *Allium sativum* L. [Amaryllidaceae] | gentamicin | male Wistar rats | 250 mg/kg/d for 21d, ig | 80 mg/kg/d for 21d, ip | SrCr↓, BUN↓, GFR↑, TNF-α↓, IL-6↓, INF-γ↓, MDA↓ Kim-1↓, SOD↑ | + | (Galal and Abd El-Rady, 2019) |
| In vivo | cinnamic acid | *Neolitsea cassia* (L.) Kosterm*.* [Lauraceae] | gentamicin | male Wistar rats | 50 mg/kg/d for 12d, ig | 100 mg/kg/d for 12d, ig | SrCr↓, BUN↓, MDA↓, CAT↑, GSH↑, GPx↑, NO↓ | + | (Babaeenezhad et al., 2021) |
| In vivo | daphnetin | *Chaenomeles speciosa* (Sweet) Nakai [Rosaceae] et al. | gentamicin | ICR mice | 40 mg/kg/d for 7d , ip | 100, 200 mg/kg/d for 7d, ip | SrCr↓, BUN↓, MDA↓, GSH↑, MPO↓, Nrf2↑, keap-1↓, HO-1↑, NQO1↑, GCLC↑, GCLM↑, NOX4↓, caspase-3↓, p53↓, Bax↓, Bcl-2↑, NF-κB↓ | + | (Fan et al., 2021) |
| In vivo and in vitro | panax notoginseng saponins | *Panax notoginseng* (Burkill) F.H.Chen [Araliaceae] | polymyxin E | female ICR mice/ TCMK-1 cells | 10 mg/kg/d for 14d, im; 0, 50, 100, 200 μg/ml | 15 mg/kg for 14d, im; 150 μg/ml | SrCr↓, BUN↓, SOD↑, MDA↓, GSH↑, GPx↑, caspase-3/9↓, Bax↓, Bcl-2↑, mitochondrial membrane↑ | + | (Zhang et al., 2019) |
| In vivo | curcumin | *Curcuma aromatica* Salisb. [Zingiberaceae] et al. | polymyxin E | BALB/c mice | 10, 100, 200 mg/kg/d for 7d, ip | 15 mg/kg for 7d, im | SrCr↓, BUN↓, ROS↓, LPO↓, GSH↑, GSSG↓, | + | (Vazin et al., 2020) |
| In vivo and in vitro | silybum marianum | *Silybum marianum* (L.) Gaertn. [Asteraceae] | vancomycin | male Swiss albino mice/ Vero cells | 400 mg/kg/d for 10d, ip; 400 mg/ml | 300 mg/kg/d for 10d, ip; 0, 1, 2, 3, 4, 5, 6, 7, 8, 9, 10 mg/ml | SrCr↓, BUN↓, p53↓, p21↓, Cyto c↓ | + | (Malkani et al., 2020) |
| In vivo | punica granatum peel ethanol extract | *Punica granatum* L. [Lythraceae] | vancomycin | male SD rats | 100 mg/kg/d for 14d, ig | 443.6 mg/kg/d every alternate day for 14d, ip | SrCr↓, BUN↓, MDA↓, SOD↑, CAT↑, GSH↑, CRP↓, caspase-3↓, Bax↓, Bcl-2↑, | + | (El Bohi et al., 2021) |

**Table 3 NSAIDs induced DI-AKI**

| **Type** | **Herbal/ Active ingredients/ Formula** | **Species, Source** | **Drug** | **Animal/Cell** | **Dose, Duration, Route of H/A/F** | **Dose, Duration, Route of drug** | **Main outcome** | **Histopathology**  **Score^a^** | **Study** |
| --- | --- | --- | --- | --- | --- | --- | --- | --- | --- |
| In vivo | yam | *Dioscorea oppositifolia* L. [Dioscoreaceae] | acetaminophen | male Wistar rats | 500 and 100 mg/kg for single time, ig | 1200 mg/kg for single injection, ip | SrCr↓, BUN↓, SOD↑, GPx↑, GSH↑, | + | (Lee et al., 2002) |
| In vivo | quercetin + curcumin | *Curcuma aromatica* Salisb. [Zingiberaceae] et al. | acetaminophen | male Wistar rats | 20 mg/kg/d + 50 mg/kg/d for 15d, ig | 650 mg/kg/d for 15d, ig | SrCr↓, BUN↓, SOD↑, GPx↑, GSH↑, CAT↑ | N | (Yousef et al., 2010) |
| In vivo | zingiber zerumbet Smith ethyl acetate extract | *Zingiber officinale* Roscoe [Zingiberaceae] | acetaminophen | male SD rats | 200, 400 mg/kg/d for 7d, ip | 750 mg/kg/d for 7d, ig | SrCr↓, BUN↓, MDA↓, SOD↑, GSH↑ | + | (Abdul Hamid et al., 2012) |
| In vivo | Jujubae Fructus | *Ziziphus jujuba* Mill. [Rhamnaceae] | ibuprofen | male SD rats | 500 mg/kg/d for 5d, ig | 400 mg/kg/d for 5d, ig | SrCr↓, BUN↓, MDA↓, GST↑, CAT↑ | + | (Awad et al., 2014) |
| In vivo | galangin | *Alpinia officinarum* Hance [Zingiberaceae] | acetaminophen | male BALB/c mice | 125 mg/kg for single dose, ig | 600, 1200 mg/kg for single dose, ip | SrCr↓, BUN↓, GSH↑ | + | (Tsai et al., 2015) |
| In vivo | cinnamomum zeylanicum L. | *Neolitsea cassia* (L.) Kosterm. [Lauraceae] | acetaminophen | BALB/c mice | 200 mg/kg/d for 14d, ig | 200 mg/kg BW for single dose, ig | SrCr↓, BUN↓, TAC↑, TOS↓ | + | (Hussain et al., 2019) |
| In vivo and in vitro | sika deer antler protein | *Cornu Cervi Pantotrichum* | acetaminophen | male C57BL/6 mice/ HK-2 cells | 40, 80, 160 mg/kg for 10d, ig; 2 mg/mL | 300 mg/kg for single injection, ip; 10 mM | SrCr↓, BUN↓, Kim-1↓, GSH↑, NGAL↓, SOD↑, Cyto c↓, CAT↑, MDA↓, Nrf2↑, NQO1↑, caspase-3↓, Bax↓, Bcl-2↑, HO-1↑, FoxO1↓, PI3K↑, Akt↑ | + | (Ruan et al., 2019) |
| In vivo | corn silk methanolic extract | *Zea mays* L. [Poaceae] | acetaminophen | male Wistar rats | 400 mg/kg/d for 5w, ig | 2 g/kg for single dose, ig | SrCr↓, BUN↓, MDA↓, SOD↑, GPx↑, caspase-3↓, NF-κB↓, SIRT5↑, Nrf2↑,  TGFβ 1↑, PCNA↑ | + | (Wans et al., 2021) |

**Table 4 Contrast induced DI-AKI**

| **Type** | **Herbal/ Active ingredients/ Formula** | **Species, Source** | **Drug** | **Animal/Cell** | **Dose, Duration, Route of H/A/F** | **Dose, Duration, Route of drug** | **Main outcome** | **Histopathology**  **Score^a^** | **Study** |
| --- | --- | --- | --- | --- | --- | --- | --- | --- | --- |
| In vivo | astragaloside IV | *Astragalus mongholicus* Bunge [Fabaceae] | iopamidol | male SD rats | 20 mg/kg/d for 7d, ig | 2.9 g iodine/kg for single injection, iv | SrCr↓, BUN↓, cystatin C↓, Kim↓, p38 MAPK↓, caspase-3↓, Bcl-2↑, Bax↓ | + | (Gui et al., 2013) |
| In vivo | tetramethylpyrazine | *Conioselinum anthriscoides 'Chuanxiong'* [Apiaceae] et al. | iohexol | male SD rats | 80 mg/kg/d for 4d, ip | 1.5-2g iodine/kg for single injection, ip | SrCr↓, BUN↓, p-p38 MAPK↓, FoxO1↓, Bcl-2↑, Bax↓, iNOS↓, CysC↓, UNAG↓, UGGT↓ | N | (Gong et al., 2013) |
| In vivo | Xuezhikang powder | *Monascus purpureus* Went. [Aspergillaceae] | iohexol | male SD rats | 2400 mg/kg/d for 3d, ig | 20 ml/kg for single injection, iv | SrCr↓, BUN↓, MDA↓, NO↑, TNF-ɑ↓, IL-6↓ | + | (Chu et al., 2016) |
| In vivo | tetramethylpyrazine | *Conioselinum anthriscoides 'Chuanxiong'* [Apiaceae] et al. | iohexol | male SD rats | 80 mg/kg/d for 4d, ip | 1.5-2g iodine/kg for single injection, ip | SrCr↓, BUN↓, Drp1↓, Mfn2↑, CCL2↓, CCR2↓, LC3B-II/I↓, Beclin-1↓, p62↑, procaspase 9↑, caspase 3↓, TNF-α↓, ROS↓, IL-6↓, CysC↓, UNAG↓, UGGT↓ | N | (Gong et al., 2019) |
| In vivo | Xuezhikang  powder | *Monascus purpureus* Went. [Aspergillaceae] | ioversol | male SD rats | 2400 mg/kg/d for 3d, ig | 3 g/kg or single injection, iv | SrCr↓, BUN↓, NGAL↓, Kim-1↓, MDA↓, SOD↑, TNF-ɑ↓, IL-6↓ | + | (Zhou et al., 2021) |

**Table 5 Other drugs induced DI-AKI**

| **Type** | **Herbal/ Active ingredients/ Formula** | **Species, Source** | **Drug** | **Animal/Cell** | **Dose, Duration, Route of H/A/F** | **Dose, Duration, Route of drug** | **Main outcome** | **Histopathology**  **Score^a^** | **Study** |
| --- | --- | --- | --- | --- | --- | --- | --- | --- | --- |
| In vivo and in vitro | schisandrin B | *Schisandra chinensis* (Turcz.) Baill. [Schisandraceae] | cyclosporine A | male KM mice/ HK-2 cells | 20 mg/kg/d for 4w, ig; 2.5, 5, 10 μM | 30 mg/kg/d for 4w, sc; 10 μM | SrCr↓, BUN↓, MDA↓, GSH↑, ROS↓, apoptosis↓ | + | (Zhu et al., 2012) |
| In vivo and in vitro | curcumin | *Curcuma aromatica* Salisb. [Zingiberaceae] et al. | cyclosporine A | male SD rats/ HK-2 cells | 30 mg/kg/d for 21d, ig; 10, 50, 100 µM | 20 mg/kg/d for 21d, sc; 2 µM | SrCr↓, BUN↓, GSH↑, SOD↑, MDA↓, ROS↓, CAT↑, Bax↓, Bcl-2↑, NF-κB↓ | + | (Huang et al., 2018) |
| In vivo | Schisandrae chinensis fructus extract | *Schisandra chinensis* (Turcz.) Baill. [Schisandraceae] | cyclosporine A | rats | 216 mg/kg/d for 7d, ig | 50 mg/kg/d for 7d, ig | SrCr↓, BUN↓, GSH↑, SOD↑, MDA↓, Nrf2↑, CAT↑, Bax↓, Bcl-2↑ | + | (Wei et al., 2021) |
| In vitro | Radix Ginseng extract | *Panax ginseng* C.A.Mey [Araliaceae] | aristolochic acid | HK-2 cells | 5, 50 µg/mL | 50 µM | cell proliferation↑, apoptosis↓, ROS↓ | N | (Bunel et al., 2015) |
| In vivo | panaxydol | *Panax ginseng* C.A.Mey [Araliaceae] | aristolochic acid | male Wistar rats | 10, 20 mg/kg/d for 20d, ig | 10 mg/kg/d for 10d, ip | SrCr↓, BUN↓, Nrf2↑, NQO1↑, HO-1↑, Keap1↓, 4HNE↓, NOX-4↓, SOD↑, CAT↑, GPx↑, LPO↓ | N | (Guo et al., 2021) |
| In vitro | tetramethylpyrazine | *Conioselinum anthriscoides 'Chuanxiong'* [Apiaceae] et al. | sodium arsenite | HK-2 cells | 50μM, 100μM | 2.5, 5, 10 µM | ROS↓, GSH↑, β-catenin↓, NF-κB↓, p38 MAPK↓, COX-2↓, TNF-α↓, cytochrome c oxidase↑, mitochondrial membrane potential↑ | N | (Gong et al., 2015) |
| In vivo | curcumin | *Curcuma aromatica* Salisb. [Zingiberaceae] et al. | arsenic trioxide | Sanshui white ducks | 400 mg/kg/d for 28d, ig | 4, 8 mg/kg/d for 28d, ig | MDA↓, Nrf2↑, caspase-3↓, Bax↓, p53↓, p62↑, Cyto c↓, mTOR↑, LC3II/LC3I↓, Atg-5↓, Beclin1↓, Pink1↓, Parkin↓, SOD↑, GSH↑, CAT↑ | + | (Wu et al., 2021) |
| In vivo | tanshinone IIA | *Salvia miltiorrhiza* Bunge [Lamiaceae] | folic acid | C57BL/6 mice | 15 mg/kg/d for 2d, iv | 250 mg/kg for single injection, ip | SrCr↓, BUN↓, PCNA↑, NGAL↓, Bcl-2↑, caspase-3↓, iNOS↓, MCP-1↓, fibronectin↓, collagen I↓ | + | (Jiang et al., 2016) |
| In vivo | licorice extract | *Glycyrrhiza glabra* L. [Fabaceae] | brucine | male SD rats | 3.6 g/kg/d for 7d, ig | 50 mg/kg/d for 7d, ip | SrCr↓, BUN↓, MDA↓, SOD↑, GSH↑, caspase-3/8/9↓, Bax↓, Bcl-2↑, STAT3↓, Akt↓, Fas↓, Cyto c↓ | + | (Zhang et al., 2019) |

**Table 6 Composition of herbal formulas**

| **Herbal formulas** | **Composition** |
| --- | --- |
| jian-pi yi-qi li-shui decoction | *Astragali Radix*, *Rhizoma Atractylodis Macrocephatae*, *Rhizoma Alismatis*, *Polyporus*, et al. |
| VI-28 | *Radix ginseng*, *Cornu cervi*, *Cordyceps*, *Radix salviae*, *Semen allii*, *Fructus cnidii*, *Fructus evodiae*, *Rhizoma kaempferiae* |
| Zhibai Dihuang Wan | *Anemarrhenae Rhizoma*, *Cortex Phellodendri Chinensis*, *Rehmanniae Radix*, *Dioscoreaopposita*, *Cornus officinalis Sieb.et Zucc.*, *Rhizoma Alismatis*, *Moutan Cortex*, *Poria cocos (Schw.) Wolf* |
| Chungsimyeonja-tang | *Nelumbo nucifera*, *Dioscorea japonica*, *Asparagus cochinchinensis*, *Liriope platyphylla*, *Polygala tenuifolia*, *Acorus gramineus*, *Zizyphus jujuba*, *Dimocarpus longan*, *Thuja orientalis*, *Scutellaria baicalensis*, *Raphanus sativus*, *Chrysanthemum indicum* |
| Bu-zhong-yi-qi decoction | *Astragali Radix*, *Glycyrrhizae Radix et Rhizoma*, *Radix Codonopsis*, *Angelicae Sinensis Radix*, *Citri Reticulatae Pericarpium*, *Cimicifugae Rhizoma*, *Bupleuri Radix*, *Rhizoma Atractylodis Macrocephata* |
| Zhen-Wu-Tang | *Zingiber officinale Roscoe*, *Paeoniae Radix Alba*, *Poria cocos (Schw.) Wolf*, *Rhizoma Atractylodis Macrocephatae*, *Aconiti Lateralis Radix Praeparata* |
| Huaiqihuang extractum | *Trametes robiniophila Murr.*, *Lycium barbarum*, *Polygonatum sibiricum* |
| Inchenhao-tang | *Artemisiae Scopariae Herba*, *Gardeniae Fructus*, *Rheum officinale Baill.* |
| Liuweidihuang-tang, | *Rehmanniae Radix*, *Dioscoreaopposita*, *Cornus officinalis Sieb.et Zucc.*, *Rhizoma Alismatis*, *Poria cocos (Schw.) Wolf*, *Moutan Cortex* |
| Longdanxiegan-tang | *Radix Gentianae*, *Gardeniae Fructus*, *Scutellariae Radix*, *Rehmannia glutinosa Libosch*, *Plantaginis Herba*, *Rhizoma Alismatis*, *Akebiae Caulis*, *Glycyrrhizae Radix et Rhizoma*, *Angelicae Sinensis Radix* |

*a: No score (N), pathological score reduced (+), no change in pathological score (-).

Abbreviation: Bcl-2, B cell lymphoma-2; BNIP3, adenovirus E1B 19 kDa-interacting protein 3; BUN, serum urea nitrogen; COX2, cyclooxygenase-2; EGF, epidermal growth factor; FIS1, mitochondrial fission 1 protein; FoxO1, Forkhead box O 1 transcription factors; GCL, glutamate cysteine ligase; GFR, glomerular filtration rate; GPx, glutathione peroxidase; HIF-1, hypoxia inducible factor; HO-1, heme oxygenase-1; HSPs, heat shock proteins; IGFBP7, IGF binding protein 7;IGF1R, IGFBP7 to IGF1 receptor; IS, Indoxyl sulfate; IMA, ischemia-modified albumin; JNK, c-Jun N-terminal kinase; Kim-1, kidney injury molecule-1; MDA, malondialdehyde; MPO, myeloperoxidase; MTP, micro total protein; NAG, N-acetyl-h-D-glucosaminidase; NGAL, neutrophil gelatinase-associated lipocalin; Nrf2, NF-E2-related factor 2; NF-kappaB, nuclear factor-kappa B; NQO1, NAD(P)H:(quinone acceptor) oxidoreductase 1; OPA1, optic atrophy 1 protein; OSI, oxidative stress index; PARP, poly(ADP-ribose) polymerase; PCNA, proliferating cell nuclear antigen; PINK1, (PTEN)-induced putative kinase protein 1; RPF, renal plasma flow; SBP-1, selenium binding protein-1; SOD, superoxide dismutase; TAC, total antioxidant capacity; TECs, tubular epithelial cells; TDT, terminal deoxynucleotidyl transferase; TGF-β, transforming growth factor-beta; TOS, total oxidant status; TOX, total antioxidant activity; 3-NY, 3-nitrotyrosin; 4-HNE, 4-hydroxynonenal; 8-OHdG, 8-hydroxy-2'-deoxyguanosine; α-SMA, alpha-smooth muscle actin.

**Reference**

Abd-Ellah, M. F. and A. D. Mariee. (2007). Ginkgo biloba leaf extract (EGb 761) diminishes adriamycin-induced hyperlipidaemic nephrotoxicity in rats: association with nitric oxide production. Biotechnol Appl Biochem. 46(Pt 1), 35-40. doi: 10.1042/ba20060085

Abd El-Kader, M. and R. I. Taha. (2020). Comparative nephroprotective effects of curcumin and etoricoxib against cisplatin-induced acute kidney injury in rats. Acta Histochem. 122(4), 151534. doi: 10.1016/j.acthis.2020.151534

Abd El-Twab, S. M., O. E. Hussein, W. G. Hozayen, M. Bin-Jumah and A. M. Mahmoud. (2019). Chicoric acid prevents methotrexate-induced kidney injury by suppressing NF-κB/NLRP3 inflammasome activation and up-regulating Nrf2/ARE/HO-1 signaling. Inflamm Res. 68(6), 511-523. doi: 10.1007/s00011-019-01241-z

Abdul Hamid, Z., S. B. Budin, N. Wen Jie, A. Hamid, K. Husain and J. Mohamed. (2012). Nephroprotective effects of Zingiber zerumbet Smith ethyl acetate extract against paracetamol-induced nephrotoxicity and oxidative stress in rats. J Zhejiang Univ Sci B. 13(3), 176-185. doi: 10.1631/jzus.B1100133

Ademiluyi, A. O., G. Oboh, O. B. Ogunsuyi and A. J. Akinyemi. (2012). Attenuation of gentamycin-induced nephrotoxicity in rats by dietary inclusion of ginger (Zingiber officinale) and turmeric (Curcuma longa) rhizomes. Nutr Health. 21(4), 209-218. doi: 10.1177/0260106013506668

Adeoye, B. O., E. R. Asenuga, A. A. Oyagbemi, T. O. Omobowale and A. A. Adedapo. (2018). The Protective Effect of the Ethanol Leaf Extract of Andrographis Paniculata on Cisplatin-Induced Acute Kidney Injury in Rats Through nrf2/KIM-1 Signalling Pathway. Drug Res (Stuttg). 68(1), 23-32. doi: 10.1055/s-0043-118179

Ahmed, W., A. Zaki and T. Nabil. (2015). Prevention of methotrexate-induced nephrotoxicity by concomitant administration of garlic aqueous extract in rat. Turk J Med Sci. 45(3), 507-516. doi: 10.3906/sag-1408-121

Ajami, M., S. Eghtesadi, H. Pazoki-Toroudi, R. Habibey and S. A. Ebrahimi. (2010). Effect of crocus sativus on gentamicin induced nephrotoxicity. Biol Res. 43(1), 83-90. doi:

Ajith, T. A., M. S. Aswathy and U. Hema. (2008). Protective effect of Zingiber officinale roscoe against anticancer drug doxorubicin-induced acute nephrotoxicity. Food Chem Toxicol. 46(9), 3178-3181. doi: 10.1016/j.fct.2008.07.004

Ajith, T. A., V. Nivitha and S. Usha. (2007). Zingiber officinale Roscoe alone and in combination with alpha-tocopherol protect the kidney against cisplatin-induced acute renal failure. Food Chem Toxicol. 45(6), 921-927. doi: 10.1016/j.fct.2006.11.014

Aksoy, N., Y. Dogan, M. Iriadam, M. Bitiren, E. Uzer, A. Ozgonul and S. Aksoy. (2012). Protective and therapeutic effects of licorice in rats with acute tubular necrosis. J Ren Nutr. 22(3), 336-343. doi: 10.1053/j.jrn.2011.07.002

Aldahmash, B. A., D. M. El-Nagar and K. E. Ibrahim. (2016). Reno-protective effects of propolis on gentamicin-induced acute renal toxicity in swiss albino mice. Nefrologia. 36(6), 643-652. doi: 10.1016/j.nefro.2016.06.004

Ali, B. H., M. Al-Moundhri, M. T. Eldin, A. Nemmar, S. Al-Siyabi and K. Annamalai. (2008). Amelioration of cisplatin-induced nephrotoxicity in rats by tetramethylpyrazine, a major constituent of the Chinese herb Ligusticum wallichi. Exp Biol Med (Maywood). 233(7), 891-896. doi: 10.3181/0711-rm-315

Ali, B. H., S. Al Salam, Y. Al Suleimani, M. Al Za'abi, M. Ashique, P. Manoj, M. Sudhadevi, et al. (2020). Ameliorative effect of sesamin in cisplatin-induced nephrotoxicity in rats by suppressing inflammation, oxidative/nitrosative stress, and cellular damage. Physiol Res. 69(1), 61-72. doi: 10.33549/physiolres.934142

Ali, D. A., A. M. Abdeen, M. F. Ismail and M. A. Mostafa. (2015). Histological, ultrastructural and immunohistochemical studies on the protective effect of ginger extract against cisplatin-induced nephrotoxicity in male rats. Toxicol Ind Health. 31(10), 869-880. doi: 10.1177/0748233713483198

Arjumand, W. and S. Sultana. (2011). Glycyrrhizic acid: A phytochemical with a protective role against cisplatin-induced genotoxicity and nephrotoxicity. Life Sciences. 89(13-14), 422-429. doi: 10.1016/j.lfs.2011.06.016

Awad, D. S., R. M. Ali, N. M. Mhaidat and A. M. Shotar. (2014). Zizyphus jujuba protects against ibuprofen-induced nephrotoxicity in rats. Pharm Biol. 52(2), 182-186. doi: 10.3109/13880209.2013.821665

Babaeenezhad, E., N. Nouryazdan, M. Nasri, H. Ahmadvand and M. Moradi Sarabi. (2021). Cinnamic acid ameliorate gentamicin-induced liver dysfunctions and nephrotoxicity in rats through induction of antioxidant activities. Heliyon. 7(7), e07465. doi: 10.1016/j.heliyon.2021.e07465

Baek, S. H., B. K. Shin, N. J. Kim, S. Y. Chang and J. H. Park. (2017). Protective effect of ginsenosides Rk3 and Rh4 on cisplatin-induced acute kidney injury in vitro and in vivo. J Ginseng Res. 41(3), 233-239. doi: 10.1016/j.jgr.2016.03.008

Bunel, V., M. H. Antoine, J. Nortier, P. Duez and C. Stévigny. (2015). In vitro effects of Panax ginseng in aristolochic acid-mediated renal tubulotoxicity: apoptosis versus regeneration. Planta Med. 81(5), 363-372. doi: 10.1055/s-0035-1545839

Bunel, V., M. H. Antoine, J. Nortier, P. Duez and C. Stévigny. (2015). Nephroprotective effects of ferulic acid, Z-ligustilide and E-ligustilide isolated from Angelica sinensis against cisplatin toxicity in vitro. Toxicol In Vitro. 29(3), 458-467. doi: 10.1016/j.tiv.2014.12.017

Bunel, V., M. H. Antoine, J. Nortier, P. Duez and C. Stévigny. (2015). Potential nephroprotective effects of the Chinese herb Angelica sinensis against cisplatin tubulotoxicity. Pharm Biol. 53(7), 985-994. doi: 10.3109/13880209.2014.951726

Cao, S. S., M. Yan, Z. Y. Hou, Y. Chen, Y. S. Jiang, X. R. Fan, P. F. Fang, et al. (2017). Danshen modulates Nrf2-mediated signaling pathway in cisplatin-induced renal injury. J Huazhong Univ Sci Technolog Med Sci. 37(5), 761-765. doi: 10.1007/s11596-017-1801-1

Chen, L., X. Xiong, X. Hou, H. Wei, J. Zhai, T. Xia, X. Gong, et al. (2019). Wuzhi capsule regulates chloroacetaldehyde pharmacokinetics behaviour and alleviates high-dose cyclophosphamide-induced nephrotoxicity and neurotoxicity in rats. Basic Clin Pharmacol Toxicol. 125(2), 142-151. doi: 10.1111/bcpt.13211

Cheng, C. Y., Y. M. Sue, C. H. Chen, C. C. Hou, P. Chan, Y. L. Chu, T. H. Chen, et al. (2006). Tetramethylpyrazine attenuates adriamycin-induced apoptotic injury in rat renal tubular cells NRK-52E. Planta Med. 72(10), 888-893. doi: 10.1055/s-2006-946695

Cheng, J. H. (1992). [Effect of preventive and therapeutical function of jian-pi yi-qi li-shui decoction on cisplatin nephrotoxicity in rats]. Zhongguo Zhong Xi Yi Jie He Za Zhi. 12(10), 614-616, 581-612. doi:

Chu, S., L. Hu, X. Wang, S. Sun, T. Zhang, Z. Sun, L. Shen, et al. (2016). Xuezhikang ameliorates contrast media-induced nephropathy in rats via suppression of oxidative stress, inflammatory responses and apoptosis. Ren Fail. 38(10), 1717-1725. doi: 10.1080/0886022x.2016.1207052

Dachuri, V., P. H. Song, Y. W. Kim, S. K. Ku and C. H. Song. (2020). Protective Effects of Traditional Polyherbs on Cisplatin-Induced Acute Kidney Injury Cell Model by Inhibiting Oxidative Stress and MAPK Signaling Pathway. Molecules. 25(23). doi: 10.3390/molecules25235641

Drishya, S., S. S. Dhanisha and C. Guruvayoorappan. (2021). Antioxidant-rich fraction of Amomum subulatum fruits mitigates experimental methotrexate-induced oxidative stress by regulating TNF-α, IL-1β, and IL-6 proinflammatory cytokines. Journal of food biochemistry. e13855. doi: 10.1111/jfbc.13855

Dungca, N. T. (2016). Protective effect of the methanolic leaf extract of Eclipta alba (L.) Hassk. (Asteraceae) against gentamicin-induced nephrotoxicity in Sprague Dawley rats. J Ethnopharmacol. 184, 18-21. doi: 10.1016/j.jep.2016.03.002

El-Ashmawy, N. E., N. F. Khedr, H. A. El-Bahrawy and S. A. Helal. (2018). Upregulation of PPAR-γ mediates the renoprotective effect of omega-3 PUFA and ferulic acid in gentamicin-intoxicated rats. Biomed Pharmacother. 99, 504-510. doi: 10.1016/j.biopha.2018.01.036

El Bohi, K. M., S. M. Abdel-Motal, S. R. Khalil, M. M. Abd-Elaal, M. M. M. Metwally and E. L. WM. (2021). The efficiency of pomegranate (Punica granatum) peel ethanolic extract in attenuating the vancomycin-triggered liver and kidney tissues injury in rats. Environ Sci Pollut Res Int. 28(6), 7134-7150. doi: 10.1007/s11356-020-10999-3

ElKady, A. I. and W. S. Ramadan. (2016). The aqueous extract of cinnamon bark ameliorated cisplatin-induced cytotoxicity in vero cells without compromising the anticancer efficiency of cisplatin. Biomed Pap Med Fac Univ Palacky Olomouc Czech Repub. 160(3), 363-371. doi: 10.5507/bp.2016.034

Fan, X., W. Gu, Y. Gao, N. Ma, C. Fan and X. Ci. (2021). Daphnetin ameliorated GM-induced renal injury through the suppression of oxidative stress and apoptosis in mice. Int Immunopharmacol. 96, 107601. doi: 10.1016/j.intimp.2021.107601

Galal, H. M. and N. M. Abd El-Rady. (2019). Aqueous garlic extract supresses experimental gentamicin induced renal pathophysiology mediated by oxidative stress, inflammation and Kim-1. Pathophysiology. 26(3-4), 271-279. doi: 10.1016/j.pathophys.2019.07.002

Gong, X., Y. Duan, J. Zheng, Z. Ye and T. K. Hei. (2019). Tetramethylpyrazine Prevents Contrast-Induced Nephropathy via Modulating Tubular Cell Mitophagy and Suppressing Mitochondrial Fragmentation, CCL2/CCR2-Mediated Inflammation, and Intestinal Injury. Oxid Med Cell Longev. 2019, 7096912. doi: 10.1155/2019/7096912

Gong, X., V. N. Ivanov, M. M. Davidson and T. K. Hei. (2015). Tetramethylpyrazine (TMP) protects against sodium arsenite-induced nephrotoxicity by suppressing ROS production, mitochondrial dysfunction, pro-inflammatory signaling pathways and programed cell death. Arch Toxicol. 89(7), 1057-1070. doi: 10.1007/s00204-014-1302-y

Gong, X., Q. Wang, X. Tang, Y. Wang, D. Fu, H. Lu, G. Wang, et al. (2013). Tetramethylpyrazine prevents contrast-induced nephropathy by inhibiting p38 MAPK and FoxO1 signaling pathways. Am J Nephrol. 37(3), 199-207. doi: 10.1159/000347033

Gui, D., J. Huang, W. Liu, Y. Guo, W. Xiao and N. Wang. (2013). Astragaloside IV prevents acute kidney injury in two rodent models by inhibiting oxidative stress and apoptosis pathways. Apoptosis. 18(4), 409-422. doi: 10.1007/s10495-013-0801-2

Gulec, M., M. Iraz, H. R. Yilmaz, H. Ozyurt and I. Temel. (2006). The effects of ginkgo biloba extract on tissue adenosine deaminase, xanthine oxidase, myeloperoxidase, malondialdehyde, and nitric oxide in cisplatin-induced nephrotoxicity. Toxicol Ind Health. 22(3), 125-130. doi: 10.1191/0748233705th255oa

Guo, Y., M. Hu, J. Ma, A. Chinnathambi, S. A. Alharbi, O. H. M. Shair and P. Ge. (2021). Protective effect of panaxydol against repeated administration of aristolochic acid on renal function and lipid peroxidation products via activating Keap1-Nrf2/ARE pathway in rat kidney. J Biochem Mol Toxicol. 35(1), e22619. doi: 10.1002/jbt.22619

Guo, Y., M. Wang, J. Mou, Z. Zhao, J. Yang, F. Zhu, G. Pei, et al. (2018). Pretreatment of Huaiqihuang extractum protects against cisplatin-induced nephrotoxicity. Sci Rep. 8(1), 7333. doi: 10.1038/s41598-018-25610-6

Han, M. S., I. H. Han, D. Lee, J. M. An, S. N. Kim, M. S. Shin, N. Yamabe, et al. (2016). Beneficial effects of fermented black ginseng and its ginsenoside 20(S)-Rg3 against cisplatin-induced nephrotoxicity in LLC-PK1 cells. J Ginseng Res. 40(2), 135-140. doi: 10.1016/j.jgr.2015.06.006

He, L., X. Peng, J. Zhu, G. Liu, X. Chen, C. Tang, H. Liu, et al. (2015). Protective effects of curcumin on acute gentamicin-induced nephrotoxicity in rats. Can J Physiol Pharmacol. 93(4), 275-282. doi: 10.1139/cjpp-2014-0459

He, L., Y. Zhang, N. Kang, Y. Wang, Z. Zhang, Z. Zha, S. Yang, et al. (2019). Anemoside B4 attenuates nephrotoxicity of cisplatin without reducing anti-tumor activity of cisplatin. Phytomedicine. 56, 136-146. doi: 10.1016/j.phymed.2018.10.035

Hsu, D. Z., K. T. Chen, T. H. Lin, Y. H. Li and M. Y. Liu. (2007). Sesame oil attenuates Cisplatin-induced hepatic and renal injuries by inhibiting nitric oxide-associated lipid peroxidation in mice. Shock. 27(2), 199-204. doi: 10.1097/01.shk.0000238063.54332.06

Hsu, D. Z., Y. H. Li, P. Y. Chu, S. Periasamy and M. Y. Liu. (2011). Sesame oil prevents acute kidney injury induced by the synergistic action of aminoglycoside and iodinated contrast in rats. Antimicrob Agents Chemother. 55(6), 2532-2536. doi: 10.1128/aac.01597-10

Hsu, Y. H., T. H. Chen, M. Y. Wu, Y. F. Lin, W. L. Chen, T. H. Cheng and C. H. Chen. (2014). Protective effects of Zhibai Dihuang Wan on renal tubular cells affected with gentamicin-induced apoptosis. J Ethnopharmacol. 151(1), 635-642. doi: 10.1016/j.jep.2013.11.031

Hu, J. N., X. Y. Xu, S. Jiang, Y. Liu, Z. Liu, Y. P. Wang, X. J. Gong, et al. (2020). Protective effect of ginsenoside Rk1, a major rare saponin from black ginseng, on cisplatin-induced nephrotoxicity in HEK-293 cells. Kaohsiung J Med Sci. 36(9), 732-740. doi: 10.1002/kjm2.12220

Huang, J., X. Yao, G. Weng, H. Qi and X. Ye. (2018). Protective effect of curcumin against cyclosporine A‑induced rat nephrotoxicity. Mol Med Rep. 17(4), 6038-6044. doi: 10.3892/mmr.2018.8591

Huang, S. J., J. Huang, Y. B. Yan, J. Qiu, R. Q. Tan, Y. Liu, Q. Tian, et al. (2020). The renoprotective effect of curcumin against cisplatin-induced acute kidney injury in mice: involvement of miR-181a/PTEN axis. Ren Fail. 42(1), 350-357. doi: 10.1080/0886022x.2020.1751658

Huang, T. H., T. H. Wu, Y. H. Guo, T. L. Li, Y. L. Chan and C. J. Wu. (2019). The concurrent treatment of Scutellaria baicalensis Georgi enhances the therapeutic efficacy of cisplatin but also attenuates chemotherapy-induced cachexia and acute kidney injury. J Ethnopharmacol. 243, 112075. doi: 10.1016/j.jep.2019.112075

Hui, D., T. Rui-Zhi, L. Jian-Chun, Z. Xia, W. Dan, F. Jun-Ming and W. Li. (2020). Astragalus propinquus Schischkin and Panax notoginseng (A&P) compound relieved cisplatin-induced acute kidney injury through inhibiting the mincle maintained macrophage inflammation. J Ethnopharmacol. 252, 112637. doi: 10.1016/j.jep.2020.112637

Hussain, Z., J. A. Khan, A. Arshad, P. Asif, H. Rashid and M. I. Arshad. (2019). Protective effects of Cinnamomum zeylanicum L. (Darchini) in acetaminophen-induced oxidative stress, hepatotoxicity and nephrotoxicity in mouse model. Biomed Pharmacother. 109, 2285-2292. doi: 10.1016/j.biopha.2018.11.123

Jafaripour, L., R. Naserzadeh, E. Alizamani, S. M. Javad Mashhadi, E. R. Moghadam, N. Nouryazdan and H. Ahmadvand. (2021). Effects of Rosmarinic Acid on Methotrexate-induced Nephrotoxicity and Hepatotoxicity in Wistar Rats. Indian J Nephrol. 31(3), 218-224. doi: 10.4103/ijn.IJN_14_20

Jamshidzadeh, A., R. Heidari, T. Golzar and A. Derakhshanfar. (2016). Effect of Eisenia foetida Extract against Cisplatin-Induced Kidney Injury in Rats. J Diet Suppl. 13(5), 551-559. doi: 10.3109/19390211.2015.1124163

Jariyawat, S., P. Kigpituck, K. Suksen, A. Chuncharunee, A. Chaovanalikit and P. Piyachaturawat. (2009). Protection against cisplatin-induced nephrotoxicity in mice by Curcuma comosa Roxb. ethanol extract. J Nat Med. 63(4), 430-436. doi: 10.1007/s11418-009-0345-5

Jeong, J. C., W. M. Hwang, C. H. Yoon and Y. K. Kim. (2001). Salviae radix extract prevents cisplatin-induced acute renal failure in rabbits. Nephron. 88(3), 241-246. doi: 10.1159/000045996

Jiang, C., W. Zhu, Q. Shao, X. Yan, B. Jin, M. Zhang and B. Xu. (2016). Tanshinone IIA Protects Against Folic Acid-Induced Acute Kidney Injury. Am J Chin Med. 44(4), 737-753. doi: 10.1142/s0192415x16500403

Jin, J., M. Li, Z. Zhao, X. Sun, J. Li, W. Wang, M. Huang, et al. (2015). Protective effect of Wuzhi tablet (Schisandra sphenanthera extract) against cisplatin-induced nephrotoxicity via Nrf2-mediated defense response. Phytomedicine. 22(5), 528-535. doi: 10.1016/j.phymed.2015.03.003

Joy, J. and C. K. Nair. (2008). Amelioration of cisplatin induced nephrotoxicity in Swiss albino mice by Rubia cordifolia extract. J Cancer Res Ther. 4(3), 111-115. doi: 10.4103/0973-1482.43139

Ju, S. M., M. S. Kim, Y. S. Jo, Y. M. Jeon, J. S. Bae, H. O. Pae and B. H. Jeon. (2017). Licorice and its active compound glycyrrhizic acid ameliorates cisplatin-induced nephrotoxicity through inactivation of p53 by scavenging ROS and overexpression of p21 in human renal proximal tubular epithelial cells. Eur Rev Med Pharmacol Sci. 21(4), 890-899. doi:

Juan, S. H., C. H. Chen, Y. H. Hsu, C. C. Hou, T. H. Chen, H. Lin, Y. L. Chu, et al. (2007). Tetramethylpyrazine protects rat renal tubular cell apoptosis induced by gentamicin. Nephrol Dial Transplant. 22(3), 732-739. doi: 10.1093/ndt/gfl699

Kalkan, Y., K. A. Kapakin, A. Kara, T. Atabay, A. Karadeniz, N. Simsek, E. Karakus, et al. (2012). Protective effect of Panax ginseng against serum biochemical changes and apoptosis in kidney of rats treated with gentamicin sulphate. J Mol Histol. 43(5), 603-613. doi: 10.1007/s10735-012-9412-4

Kalra, P., R. Karwasra, Y. K. Gupta, S. B. Ray and S. Singh. (2019). Terminalia chebula supplementation attenuates cisplatin-induced nephrotoxicity in Wistar rats through modulation of apoptotic pathway. Nat Prod Res. 33(11), 1641-1645. doi: 10.1080/14786419.2018.1425843

Kandemir, F. M., S. Yildirim, C. Caglayan, S. Kucukler and G. Eser. (2019). Protective effects of zingerone on cisplatin-induced nephrotoxicity in female rats. Environ Sci Pollut Res Int. 26(22), 22562-22574. doi: 10.1007/s11356-019-05505-3

Karwasra, R., P. Kalra, Y. K. Gupta, D. Saini, A. Kumar and S. Singh. (2016). Antioxidant and anti-inflammatory potential of pomegranate rind extract to ameliorate cisplatin-induced acute kidney injury. Food Funct. 7(7), 3091-3101. doi: 10.1039/c6fo00188b

Kim, D. U., D. G. Kim, J. W. Choi, J. Y. Shin, B. Kweon, Z. Zhou, H. S. Lee, et al. (2021). Loganin Attenuates the Severity of Acute Kidney Injury Induced by Cisplatin through the Inhibition of ERK Activation in Mice. Int J Mol Sci. 22(3). doi: 10.3390/ijms22031421

Kim, Y. J., T. W. Kim, C. S. Seo, S. R. Park, H. Ha, H. K. Shin and J. Y. Jung. (2014). Quatification of flavonoid contents in Chungsimyeonja-tang, a multi-herbal decoction, and its protective effect against cisplatin-induced nephrotoxicity. Natural Product Sciences. 20(4), 251-257. doi:

Kim, Y. J., M. Y. Lee, H. Y. Son, B. K. Park, S. Y. Ryu and J. Y. Jung. (2014). Red ginseng ameliorates acute cisplatin-induced nephropathy. Planta Med. 80(8-9), 645-654. doi: 10.1055/s-0034-1368571

Kuhad, A., S. Pilkhwal, S. Sharma, N. Tirkey and K. Chopra. (2007). Effect of curcumin on inflammation and oxidative stress in cisplatin-induced experimental nephrotoxicity. J Agric Food Chem. 55(25), 10150-10155. doi: 10.1021/jf0723965

Kumar, P., K. Sulakhiya, C. C. Barua and N. Mundhe. (2017). TNF-α, IL-6 and IL-10 expressions, responsible for disparity in action of curcumin against cisplatin-induced nephrotoxicity in rats. Mol Cell Biochem. 431(1-2), 113-122. doi: 10.1007/s11010-017-2981-5

Lee, C. K., K. K. Park, A. S. Chung and W. Y. Chung. (2012). Ginsenoside Rg3 enhances the chemosensitivity of tumors to cisplatin by reducing the basal level of nuclear factor erythroid 2-related factor 2-mediated heme oxygenase-1/NAD(P)H quinone oxidoreductase-1 and prevents normal tissue damage by scavenging cisplatin-induced intracellular reactive oxygen species. Food Chem Toxicol. 50(7), 2565-2574. doi: 10.1016/j.fct.2012.01.005

Lee, D., J. S. Yu, S. R. Lee, G. S. Hwang, K. S. Kang, J. G. Park, H. Y. Kim, et al. (2018). Beneficial Effects of Bioactive Compounds in Mulberry Fruits against Cisplatin-Induced Nephrotoxicity. Int J Mol Sci. 19(4). doi: 10.3390/ijms19041117

Lee, H. L. and K. S. Kang. (2017). Protective effect of ginsenoside Rh3 against anticancer drug-induced apoptosis in LLC-PK1 kidney cells. J Ginseng Res. 41(2), 227-231. doi: 10.1016/j.jgr.2017.01.011

Lee, S. C., C. C. Tsai, J. C. Chen, J. G. Lin, C. C. Lin, M. L. Hu and S. Lu. (2002). Effects of "Chinese yam" on hepato-nephrotoxicity of acetaminophen in rats. Acta Pharmacol Sin. 23(6), 503-508. doi:

Li, L. S., F. Zheng and Z. H. Liu. (1996). [Experimental study on effect of Cordyceps sinensis in ameliorating aminoglycoside induced nephrotoxicity]. Zhongguo Zhong Xi Yi Jie He Za Zhi. 16(12), 733-737. doi:

Li, Q., X. Liang, Y. Yang, X. Zeng, X. Zhong and C. Huang. (2020). Panax notoginseng saponins ameliorate cisplatin-induced mitochondrial injury via the HIF-1α/mitochondria/ROS pathway. FEBS Open Bio. 10(1), 118-126. doi: 10.1002/2211-5463.12760

Li, Q., Y. Zhang, Y. Yang, S. Huang, X. Zou, C. Wei, T. Liang, et al. (2021). Panax notoginseng saponins reduces the cisplatin-induced acute renal injury by increasing HIF-1α/BNIP3 to inhibit mitochondrial apoptosis pathway. Biomed Pharmacother. 142, 111965. doi: 10.1016/j.biopha.2021.111965

Li, R. Y., W. Z. Zhang, X. T. Yan, J. G. Hou, Z. Wang, C. B. Ding, W. C. Liu, et al. (2019). Arginyl-fructosyl-glucose, a Major Maillard Reaction Product of Red Ginseng, Attenuates Cisplatin-Induced Acute Kidney Injury by Regulating Nuclear Factor κB and Phosphatidylinositol 3-Kinase/Protein Kinase B Signaling Pathways. J Agric Food Chem. 67(20), 5754-5763. doi: 10.1021/acs.jafc.9b00540

Li, W., M. H. Yan, Y. Liu, Z. Liu, Z. Wang, C. Chen, J. Zhang, et al. (2016). Ginsenoside Rg5 Ameliorates Cisplatin-Induced Nephrotoxicity in Mice through Inhibition of Inflammation, Oxidative Stress, and Apoptosis. Nutrients. 8(9). doi: 10.3390/nu8090566

Li, Y., M. Gao, L. H. Yin, L. N. Xu, Y. Qi, P. Sun and J. Y. Peng. (2021). Dioscin ameliorates methotrexate-induced liver and kidney damages via adjusting miRNA-145-5p-mediated oxidative stress. Free Radic Biol Med. 169, 99-109. doi: 10.1016/j.freeradbiomed.2021.03.035

Liu, C., Y. Kang, X. Zhou, Z. Yang, J. Gu and C. Han. (2017). Rhizoma smilacis glabrae protects rats with gentamicin-induced kidney injury from oxidative stress-induced apoptosis by inhibiting caspase-3 activation. J Ethnopharmacol. 198, 122-130. doi: 10.1016/j.jep.2016.12.034

Liu, Q., S. Hu, Y. He, J. Zhang, X. Zeng, F. Gong and L. Liang. (2017). The protective effects of Zhen-Wu-Tang against cisplatin-induced acute kidney injury in rats. PLoS One. 12(6), e0179137. doi: 10.1371/journal.pone.0179137

Liu, S. J. and S. W. Zhou. (2000). Panax notoginseng saponins attenuated cisplatin-induced nephrotoxicity. Acta Pharmacol Sin. 21(3), 257-260. doi:

Liu, X., Z. Huang, X. Zou, Y. Yang, Y. Qiu and Y. Wen. (2015). Possible mechanism of PNS protection against cisplatin-induced nephrotoxicity in rat models. Toxicol Mech Methods. 25(5), 347-354. doi: 10.3109/15376516.2015.1006492

Liu, X. H., J. Li, Q. X. Li, Y. X. Ai and L. Zhang. (2008). Protective effects of ligustrazine on cisplatin-induced oxidative stress, apoptosis and nephrotoxicity in rats. Environ Toxicol Pharmacol. 26(1), 49-55. doi: 10.1016/j.etap.2008.01.006

Ma, Z. N., Y. Z. Li, W. Li, X. T. Yan, G. Yang, J. Zhang, L. C. Zhao, et al. (2017). Nephroprotective Effects of Saponins from Leaves of Panax quinquefolius against Cisplatin-Induced Acute Kidney Injury. Int J Mol Sci. 18(7). doi: 10.3390/ijms18071407

Ma, Z. N., Z. Liu, Z. Wang, S. Ren, S. Tang, Y. P. Wang, S. Y. Xiao, et al. (2017). Supplementation of American ginseng berry extract mitigated cisplatin-evoked nephrotoxicity by suppressing ROS-mediated activation of MAPK and NF-κB signaling pathways. Food Chem Toxicol. 110, 62-73. doi: 10.1016/j.fct.2017.10.006

Mahgoub, E., S. M. Kumaraswamy, K. H. Kader, B. Venkataraman, S. Ojha, E. Adeghate and M. Rajesh. (2017). Genipin attenuates cisplatin-induced nephrotoxicity by counteracting oxidative stress, inflammation, and apoptosis. Biomedicine and Pharmacotherapy. 93, 1083-1097. doi: 10.1016/j.biopha.2017.07.018

Mahran, Y. F. and H. M. Hassan. (2020). Ganoderma lucidum Prevents Cisplatin-Induced Nephrotoxicity through Inhibition of Epidermal Growth Factor Receptor Signaling and Autophagy-Mediated Apoptosis. Oxidative Medicine and Cellular Longevity. 2020. doi: 10.1155/2020/4932587

Malkani, N., A. Naeem, F. Ijaz, S. Mumtaz, S. Ashraf and M. I. Sohail. (2020). Silybum marianum (milk thistle) improves vancomycin induced nephrotoxicity by downregulating apoptosis. Mol Biol Rep. 47(7), 5451-5459. doi: 10.1007/s11033-020-05635-9

Manikandan, R., M. Beulaja, R. Thiagarajan, A. Priyadarsini, R. Saravanan and M. Arumugam. (2011). Ameliorative effects of curcumin against renal injuries mediated by inducible nitric oxide synthase and nuclear factor kappa B during gentamicin-induced toxicity in Wistar rats. Eur J Pharmacol. 670(2-3), 578-585. doi: 10.1016/j.ejphar.2011.08.037

Meng, X. M., H. D. Li, W. F. Wu, P. Ming-Kuen Tang, G. L. Ren, L. Gao, X. F. Li, et al. (2018). Wogonin protects against cisplatin-induced acute kidney injury by targeting RIPK1-mediated necroptosis. Lab Invest. 98(1), 79-94. doi: 10.1038/labinvest.2017.115

Michel, H. E. and E. T. Menze. (2019). Tetramethylpyrazine guards against cisplatin-induced nephrotoxicity in rats through inhibiting HMGB1/TLR4/NF-κB and activating Nrf2 and PPAR-γ signaling pathways. Eur J Pharmacol. 857, 172422. doi: 10.1016/j.ejphar.2019.172422

Murali, V. P. and G. Kuttan. (2016). Curculigo orchioides Gaertn Effectively Ameliorates the Uro- and Nephrotoxicities Induced by Cyclophosphamide Administration in Experimental Animals. Integr Cancer Ther. 15(2), 205-215. doi: 10.1177/1534735415607319

Naidu, M. U. R., A. A. Shifow, K. V. Kumar and K. S. Ratnakar. (2000). Ginkgo biloba extract ameliorates gentamicin-induced nephrotoxicity in rats. Phytomedicine. 7(3), 191-197. doi: 10.1016/S0944-7113(00)80003-3

Nasr, A. Y. and H. A. Saleh. (2014). Aged garlic extract protects against oxidative stress and renal changes in cisplatin-treated adult male rats. Cancer Cell Int. 14(1), 92. doi: 10.1186/s12935-014-0092-x

Negrette-Guzmán, M., W. R. García-Niño, E. Tapia, C. Zazueta, S. Huerta-Yepez, J. C. León-Contreras, R. Hernández-Pando, et al. (2015). Curcumin Attenuates Gentamicin-Induced Kidney Mitochondrial Alterations: Possible Role of a Mitochondrial Biogenesis Mechanism. Evid Based Complement Alternat Med. 2015, 917435. doi: 10.1155/2015/917435

Ortega-Domínguez, B., O. E. Aparicio-Trejo, F. E. García-Arroyo, J. C. León-Contreras, E. Tapia, E. Molina-Jijón, R. Hernández-Pando, et al. (2017). Curcumin prevents cisplatin-induced renal alterations in mitochondrial bioenergetics and dynamic. Food Chem Toxicol. 107(Pt A), 373-385. doi: 10.1016/j.fct.2017.07.018

Osman, A. M., S. A. Telity, S. A. Telity, Z. A. Damanhouri, S. E. Al-Harthy, H. M. Al-Kreathy, W. S. Ramadan, et al. (2015). Chemosensitizing and nephroprotective effect of resveratrol in cisplatin -treated animals. Cancer Cell Int. 15, 6. doi: 10.1186/s12935-014-0152-2

Parhizgar, S., S. Hosseinian, M. A. Hadjzadeh, M. Soukhtanloo, A. Ebrahimzadeh, R. Mohebbati, Z. Naji Ebrahimi Yazd, et al. (2016). Renoprotective Effect of Plantago Major Against Nephrotoxicity and Oxidative Stress Induced by Cisplatin. Iran J Kidney Dis. 10(4), 182-188. doi:

Park, J. Y., P. Choi, T. Kim, H. Ko, H. K. Kim, K. S. Kang and J. Ham. (2015). Protective Effects of Processed Ginseng and Its Active Ginsenosides on Cisplatin-Induced Nephrotoxicity: In Vitro and in Vivo Studies. J Agric Food Chem. 63(25), 5964-5969. doi: 10.1021/acs.jafc.5b00782

Park, Y. J., K. S. Kim, J. H. Park, S. H. Lee, H. R. Kim, S. H. Lee, H. B. Choi, et al. (2020). Protective effects of dendropanoxide isolated from Dendropanax morbifera against cisplatin-induced acute kidney injury via the AMPK/mTOR signaling pathway. Food Chem Toxicol. 145, 111605. doi: 10.1016/j.fct.2020.111605

Periasamy, S., C. T. Liu, D. Z. Hsu and M. Y. Liu. (2010). Sesame oil accelerates kidney healing following gentamicin-induced kidney injury in rats. Am J Nephrol. 32(5), 383-392. doi: 10.1159/000319853

Pongjit, K., C. Ninsontia, C. Chaotham and P. Chanvorachote. (2011). Protective effect of Glycine max and Chrysanthemum indicum extracts against cisplatin-induced renal epithelial cell death. Hum Exp Toxicol. 30(12), 1931-1944. doi: 10.1177/0960327111402242

Poon, M. K., P. Y. Chiu, H. Y. Leung, A. H. Siu and K. M. Ko. (2008). A 'Yang-Invigorating' Chinese herbal formula protects against gentamicin-induced nephrotoxicity in rats. Phytother Res. 22(1), 131-133. doi: 10.1002/ptr.2257

Qi, Z., W. Li, J. Tan, C. Wang, H. Lin, B. Zhou, J. Liu, et al. (2019). Effect of ginsenoside Rh(2) on renal apoptosis in cisplatin-induced nephrotoxicity in vivo. Phytomedicine. 61, 152862. doi: 10.1016/j.phymed.2019.152862

Qi, Z. L., Z. Wang, W. Li, J. G. Hou, Y. Liu, X. D. Li, H. P. Li, et al. (2017). Nephroprotective Effects of Anthocyanin from the Fruits of Panax ginseng (GFA) on Cisplatin-Induced Acute Kidney Injury in Mice. Phytother Res. 31(9), 1400-1409. doi: 10.1002/ptr.5867

Ruan, H., J. Luo, L. Wang, J. Wang, Z. Wang and J. Zhang. (2019). Sika deer antler protein against acetaminophen-induced nephrotoxicity by activating Nrf2 and inhibition FoxO1 via PI3K/Akt signaling. International Journal of Biological Macromolecules. 141, 961-986. doi: 10.1016/j.ijbiomac.2019.08.164

Russo, E. R., I. Facincani, K. C. Nakazato, T. M. Coimbra, E. J. Crevelin, A. M. S. Pereira and F. Carmona. (2018). Oral administration of powdered dried rhizomes of Curcuma longa L. (turmeric, Zingiberaceae) is effective in the treatment of doxorubicin-induced kidney injury in rats. Phytother Res. 32(12), 2408-2416. doi: 10.1002/ptr.6176

Sahu, B. D., M. Kuncha, G. J. Sindhura and R. Sistla. (2013). Hesperidin attenuates cisplatin-induced acute renal injury by decreasing oxidative stress, inflammation and DNA damage. Phytomedicine. 20(5), 453-460. doi: 10.1016/j.phymed.2012.12.001

Sakalli Çetin, E., H. Tetiker, Ö. İlhan Çelik, N. Yılmaz and H. Ciğerci İ. (2017). [Methotrexate-Induced Nephrotoxicity in Rats: Protective Effect of Mistletoe (Viscum album L.) Extract]. Complement Med Res. 24(6), 364-370. doi: 10.1159/000468984

Sandeep, D. and C. K. Krishnan Nair. (2010). Amelioration of cisplatin-induced nephrotoxicity by extracts of Hemidesmus indicus and Acorus calamus. Pharm Biol. 48(3), 290-295. doi: 10.3109/13880200903116048

Seo, C. S., T. W. Kim, Y. J. Kim, S. R. Park, H. Ha, H. K. Shin and J. Y. Jung. (2015). Trichosanthes kirilowii ameliorates cisplatin-induced nephrotoxicity in both in vitro and in vivo. Nat Prod Res. 29(6), 554-557. doi: 10.1080/14786419.2014.952229

Sharma, S., P. Sharma, P. Kulurkar, D. Singh, D. Kumar and V. Patial. (2017). Iridoid glycosides fraction from Picrorhiza kurroa attenuates cyclophosphamide-induced renal toxicity and peripheral neuropathy via PPAR-γ mediated inhibition of inflammation and apoptosis. Phytomedicine. 36, 108-117. doi: 10.1016/j.phymed.2017.09.018

Sherif, I. O., N. H. Al-Shaalan and D. Sabry. (2019). Ginkgo Biloba Extract Alleviates Methotrexate-Induced Renal Injury: New Impact on PI3K/Akt/mTOR Signaling and MALAT1 Expression. Biomolecules. 9(11). doi: 10.3390/biom9110691

Shin, H. K., T. W. Kim, Y. J. Kim, S. R. Park, C. S. Seo, H. Ha and J. Y. Jung. (2017). Protective Effects of Dendrobium nobile against Cisplatin Nephrotoxicity Both In-vitro and In-vivo. Iran J Pharm Res. 16(Suppl), 197-206. doi:

Shin, H. S., M. Yu, M. Kim, H. S. Choi and D. H. Kang. (2014). Renoprotective effect of red ginseng in gentamicin-induced acute kidney injury. Lab Invest. 94(10), 1147-1160. doi: 10.1038/labinvest.2014.101

Soetikno, V., S. D. P. Sari, L. Ul Maknun, N. K. Sumbung, D. N. I. Rahmi, B. A. W. Pandhita, M. Louisa, et al. (2019). Pre-Treatment with Curcumin Ameliorates Cisplatin-Induced Kidney Damage by Suppressing Kidney Inflammation and Apoptosis in Rats. Drug Res (Stuttg). 69(2), 75-82. doi: 10.1055/a-0641-5148

Song, K. I., J. Y. Park, S. Lee, D. Lee, H. J. Jang, S. N. Kim, H. Ko, et al. (2015). Protective effect of tetrahydrocurcumin against cisplatin-induced renal damage: in vitro and in vivo studies. Planta Med. 81(4), 286-291. doi: 10.1055/s-0035-1545696

Sue, Y. M., C. F. Cheng, C. C. Chang, Y. Chou, C. H. Chen and S. H. Juan. (2009). Antioxidation and anti-inflammation by haem oxygenase-1 contribute to protection by tetramethylpyrazine against gentamicin-induced apoptosis in murine renal tubular cells. Nephrol Dial Transplant. 24(3), 769-777. doi: 10.1093/ndt/gfn545

Tan, R. Z., J. Liu, Y. Y. Zhang, H. L. Wang, J. C. Li, Y. H. Liu, X. Zhong, et al. (2019). Curcumin relieved cisplatin-induced kidney inflammation through inhibiting Mincle-maintained M1 macrophage phenotype. Phytomedicine. 52, 284-294. doi: 10.1016/j.phymed.2018.09.210

Tomar, A., S. Vasisth, S. I. Khan, S. Malik, T. C. Nag, D. S. Arya and J. Bhatia. (2017). Galangin ameliorates cisplatin induced nephrotoxicity in vivo by modulation of oxidative stress, apoptosis and inflammation through interplay of MAPK signaling cascade. Phytomedicine. 34, 154-161. doi: 10.1016/j.phymed.2017.05.007

Topcu-Tarladacalisir, Y., M. Sapmaz-Metin and T. Karaca. (2016). Curcumin counteracts cisplatin-induced nephrotoxicity by preventing renal tubular cell apoptosis. Ren Fail. 38(10), 1741-1748. doi: 10.1080/0886022x.2016.1229996

Trujillo, J., E. Molina-Jijón, O. N. Medina-Campos, R. Rodríguez-Muñoz, J. L. Reyes, M. L. Loredo, D. Barrera-Oviedo, et al. (2016). Curcumin prevents cisplatin-induced decrease in the tight and adherens junctions: relation to oxidative stress. Food Funct. 7(1), 279-293. doi: 10.1039/c5fo00624d

Tsai, M. S., C. C. Chien, T. H. Lin, C. C. Liu, R. H. Liu, H. L. Su, Y. T. Chiu, et al. (2015). Galangin Prevents Acute Hepatorenal Toxicity in Novel Propacetamol-Induced Acetaminophen-Overdosed Mice. J Med Food. 18(11), 1187-1197. doi: 10.1089/jmf.2014.3328

Ueki, M., M. Ueno, J. Morishita and N. Maekawa. (2013). Curcumin ameliorates cisplatin-induced nephrotoxicity by inhibiting renal inflammation in mice. J Biosci Bioeng. 115(5), 547-551. doi: 10.1016/j.jbiosc.2012.11.007

Ugur, S., R. Ulu, A. Dogukan, A. Gurel, I. P. Yigit, N. Gozel, B. Aygen, et al. (2015). The renoprotective effect of curcumin in cisplatin-induced nephrotoxicity. Ren Fail. 37(2), 332-336. doi: 10.3109/0886022x.2014.986005

Ulusoy, H. B., İ. Öztürk and M. F. Sönmez. (2016). Protective effect of propolis on methotrexate-induced kidney injury in the rat. Ren Fail. 38(5), 744-750. doi: 10.3109/0886022x.2016.1158070

Vazin, A., R. Heidari and Z. Khodami. (2020). Curcumin Supplementation Alleviates Polymyxin E-Induced Nephrotoxicity. J Exp Pharmacol. 12, 129-136. doi: 10.2147/jep.s255861

Venkatesan, N., D. Punithavathi and V. Arumugam. (2000). Curcumin prevents adriamycin nephrotoxicity in rats. Br J Pharmacol. 129(2), 231-234. doi: 10.1038/sj.bjp.0703067

Wang, H., L. Kong, J. Zhang, G. Yu, G. Lv, F. Zhang, X. Chen, et al. (2014). The pseudoginsenoside F11 ameliorates cisplatin-induced nephrotoxicity without compromising its anti-tumor activity in vivo. Sci Rep. 4, 4986. doi: 10.1038/srep04986

Wang, S., S. Tang, X. Chen, X. Li, S. Jiang, H. P. Li, P. H. Jia, et al. (2020). Pulchinenoside B4 exerts the protective effects against cisplatin-induced nephrotoxicity through NF-κB and MAPK mediated apoptosis signaling pathways in mice. Chem Biol Interact. 331, 109233. doi: 10.1016/j.cbi.2020.109233

Wang, Z., Y. F. Li, X. Y. Han, Y. S. Sun, L. X. Zhang, W. Liu, X. X. Liu, et al. (2018). Kidney Protection Effect of Ginsenoside Re and Its Underlying Mechanisms on Cisplatin-Induced Kidney Injury. Cell Physiol Biochem. 48(5), 2219-2229. doi: 10.1159/000492562

Wans, E. M., M. M. Ahmed, A. A. Mousa, E. A. Tahoun and S. H. Orabi. (2021). Ameliorative effects of corn silk extract on acetaminophen-induced renal toxicity in rats. Environ Sci Pollut Res Int. 28(2), 1762-1774. doi: 10.1007/s11356-020-10588-4

Waseem, M., P. Kaushik and S. Parvez. (2013). Mitochondria-mediated mitigatory role of curcumin in cisplatin-induced nephrotoxicity. Cell Biochem Funct. 31(8), 678-684. doi: 10.1002/cbf.2955

Wei, X. M., S. Jiang, S. S. Li, Y. S. Sun, S. H. Wang, W. C. Liu, Z. Wang, et al. (2021). Endoplasmic Reticulum Stress-Activated PERK-eIF2α-ATF4 Signaling Pathway is Involved in the Ameliorative Effects of Ginseng Polysaccharides against Cisplatin-Induced Nephrotoxicity in Mice. ACS Omega. 6(13), 8958-8966. doi: 10.1021/acsomega.0c06339

Wei, Y., Z. Luo, K. Zhou, Q. Wu, W. Xiao, Y. Yu and T. Li. (2021). Schisandrae chinensis fructus extract protects against hepatorenal toxicity and changes metabolic ions in cyclosporine A rats. Nat Prod Res. 35(17), 2915-2920. doi: 10.1080/14786419.2019.1672688

Wu, S., W. Yu, X. Jiang, R. Huang, X. Zhang, J. Lan, G. Zhong, et al. (2021). Protective effects of curcumin on ATO-induced nephrotoxicity in ducks in relation to suppressed autophagy, apoptosis and dyslipidemia by regulating oxidative stress. Ecotoxicol Environ Saf. 219, 112350. doi: 10.1016/j.ecoenv.2021.112350

Wu, W. F., J. N. Wang, Z. Li, B. Wei, J. Jin, L. Gao, H. D. Li, et al. (2020). 7-Hydroxycoumarin protects against cisplatin-induced acute kidney injury by inhibiting necroptosis and promoting Sox9-mediated tubular epithelial cell proliferation. Phytomedicine. 69, 153202. doi: 10.1016/j.phymed.2020.153202

Wu, W. J., Y. F. Tang, S. Dong and J. Zhang. (2021). Ginsenoside Rb3 Alleviates the Toxic Effect of Cisplatin on the Kidney during Its Treatment to Oral Cancer via TGF-β-Mediated Mitochondrial Apoptosis. Evid Based Complement Alternat Med. 2021, 6640714. doi: 10.1155/2021/6640714

Xing, J. J., J. G. Hou, Z. N. Ma, Z. Wang, S. Ren, Y. P. Wang, W. C. Liu, et al. (2019). Ginsenoside Rb3 provides protective effects against cisplatin-induced nephrotoxicity via regulation of AMPK-/mTOR-mediated autophagy and inhibition of apoptosis in vitro and in vivo. Cell Prolif. 52(4), e12627. doi: 10.1111/cpr.12627

Xiong, Y., B. Shang, S. Xu, R. Zhao, H. Gou and C. Wang. (2016). Protective effect of Bu-zhong-yi-qi decoction, the water extract of Chinese traditional herbal medicine, on 5-fluorouracil-induced renal injury in mice. Renal Failure. 38(8), 1240-1248. doi: 10.1080/0886022X.2016.1209380

Yang, C., Y. Guo, T. S. Huang, J. Zhao, X. J. Huang, H. X. Tang, N. An, et al. (2018). Asiatic acid protects against cisplatin-induced acute kidney injury via anti-apoptosis and anti-inflammation. Biomed Pharmacother. 107, 1354-1362. doi: 10.1016/j.biopha.2018.08.126

Yang, Q., H. M. Zang, T. Xing, S. F. Zhang, C. Li, Y. Zhang, Y. H. Dong, et al. (2021). Gypenoside XLIX protects against acute kidney injury by suppressing IGFBP7/IGF1R-mediated programmed cell death and inflammation. Phytomedicine. 85, 153541. doi: 10.1016/j.phymed.2021.153541

Yokozawa, T. and E. Dong. (2001). Role of ginsenoside-Rd in cisplatin-induced renal injury: special reference to DNA fragmentation. Nephron. 89(4), 433-438. doi: 10.1159/000046116

Yokozawa, T., E. Dong, H. Oura, G. Nonaka and I. Nishioka. (1997). Magnesium lithospermate B ameliorates cisplatin-induced injury in cultured renal epithelial cells. Exp Toxicol Pathol. 49(5), 343-346. doi: 10.1016/s0940-2993(97)80104-2

Yokozawa, T. and Z. W. Liu. (2000). The role of ginsenoside-Rd in cisplatin-induced acute renal failure. Ren Fail. 22(2), 115-127. doi: 10.1081/jdi-100100858

Yousef, M. I. and H. M. Hussien. (2015). Cisplatin-induced renal toxicity via tumor necrosis factor-α, interleukin 6, tumor suppressor P53, DNA damage, xanthine oxidase, histological changes, oxidative stress and nitric oxide in rats: protective effect of ginseng. Food Chem Toxicol. 78, 17-25. doi: 10.1016/j.fct.2015.01.014

Yousef, M. I., S. A. Omar, M. I. El-Guendi and L. A. Abdelmegid. (2010). Potential protective effects of quercetin and curcumin on paracetamol-induced histological changes, oxidative stress, impaired liver and kidney functions and haematotoxicity in rat. Food Chem Toxicol. 48(11), 3246-3261. doi: 10.1016/j.fct.2010.08.034

Youssef Nasr, A. and A. Al Shahat Ibrahim. (2015). Aged garlic extract ameliorates the oxidative stress, histomorphological, and ultrastructural changes of cisplatin-induced nephrotoxicity in adult male rats. Microsc Res Tech. 78(6), 452-461. doi: 10.1002/jemt.22494

Yuluğ, E., S. Türedi, Ö. Yıldırım, E. Yenilmez, Y. Aliyazıcıoğlu, S. Demir, S. Özer-Yaman, et al. (2019). Biochemical and morphological evaluation of the effects of propolis on cisplatin induced kidney damage in rats. Biotech Histochem. 94(3), 204-213. doi: 10.1080/10520295.2018.1543895

Zhai, J., H. Gao, S. Wang, S. Zhang, X. Qu, Y. Zhang, L. Tao, et al. (2021). Ginsenoside Rg3 attenuates cisplatin-induced kidney injury through inhibition of apoptosis and autophagy-inhibited NLRP3. J Biochem Mol Toxicol. 35(11), e22896. doi: 10.1002/jbt.22896

Zhang, M., C. Wang, H. L. Cai, J. Wen and P. F. Fang. (2019). Licorice Extracts Attenuate Nephrotoxicity Induced by Brucine Through Suppression of Mitochondria Apoptotic Pathway and STAT3 Activation. Curr Med Sci. 39(6), 890-898. doi: 10.1007/s11596-019-2126-z

Zhang, Y., J. Chang, H. Gao, X. Qu, J. Zhai, L. Tao, J. Sun, et al. (2021). Huaiqihuang (HQH) granule alleviates cyclophosphamide-induced nephrotoxicity via suppressing the MAPK/NF-κB pathway and NLRP3 inflammasome activation. Pharm Biol. 59(1), 1425-1431. doi: 10.1080/13880209.2021.1990356

Zhang, Y., X. Chi, Z. Wang, S. Bi, Y. Wang, F. Shi, S. Hu, et al. (2019). Protective effects of Panax notoginseng saponins on PME-Induced nephrotoxicity in mice. Biomed Pharmacother. 116, 108970. doi: 10.1016/j.biopha.2019.108970

Zhang, Y., X. Tao, L. Yin, L. Xu, Y. Xu, Y. Qi, X. Han, et al. (2017). Protective effects of dioscin against cisplatin-induced nephrotoxicity via the microRNA-34a/sirtuin 1 signalling pathway. Br J Pharmacol. 174(15), 2512-2527. doi: 10.1111/bph.13862

Zhou, L., D. W. Liu, F. F. Zhang, S. Y. Liu, F. Wang, Z. S. Liu and Q. Zhao. (2021). [Effects and mechanisms of Xuezhikang on preventing contrast-induced acute kidney injury in diabetic rats]. Zhonghua Yi Xue Za Zhi. 101(10), 727-731. doi: 10.3760/cma.j.cn112137-20210120-00184

Zhou, Y. D., J. G. Hou, G. Yang, S. Jiang, C. Chen, Z. Wang, Y. Y. Liu, et al. (2019). Icariin ameliorates cisplatin-induced cytotoxicity in human embryonic kidney 293 cells by suppressing ROS-mediated PI3K/Akt pathway. Biomed Pharmacother. 109, 2309-2317. doi: 10.1016/j.biopha.2018.11.108

Zhu, M. M., L. Wang, D. Yang, C. Li, S. T. Pang, X. H. Li, R. Li, et al. (2019). Wedelolactone alleviates doxorubicin-induced inflammation and oxidative stress damage of podocytes by IκK/IκB/NF-κB pathway. Biomed Pharmacother. 117, 109088. doi: 10.1016/j.biopha.2019.109088

Zhu, S., Y. Wang, M. Chen, J. Jin, Y. Qiu, M. Huang and Z. Huang. (2012). Protective effect of schisandrin B against cyclosporine A-induced nephrotoxicity in vitro and in vivo. Am J Chin Med. 40(3), 551-566. doi: 10.1142/s0192415x12500425
