# Supplementary figures and images for: Potential therapeutic effects of Chinese meteria medica in mitigating drug-induced acute kidney injury
Source: Front Pharmacol. 2023 Apr 3;14:1153297. doi: 10.3389/fphar.2023.1153297 (PMC10106589; doi:10.3389/fphar.2023.1153297)

**Ginsenoside Rg1**


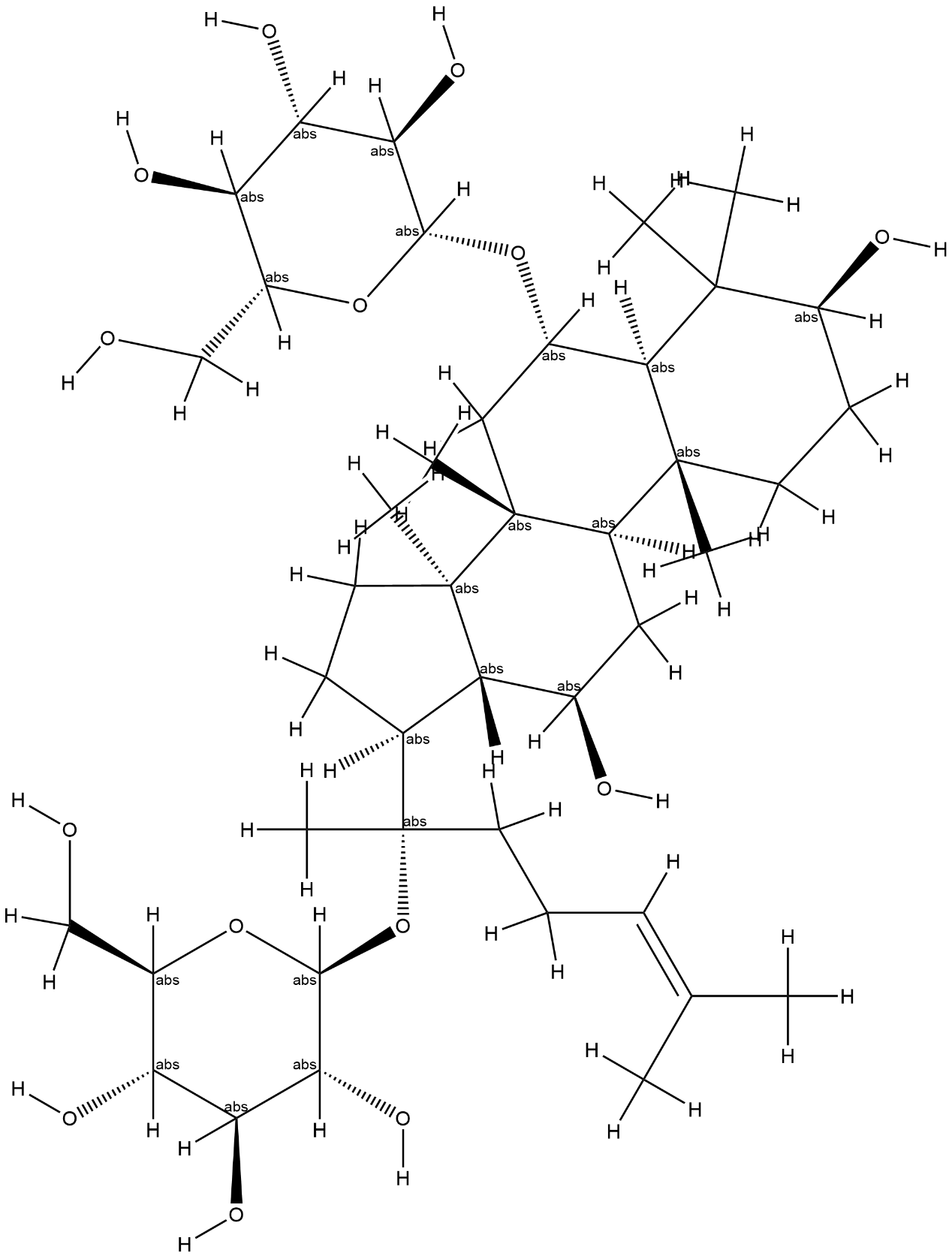


**Ginsenoside Rb1**


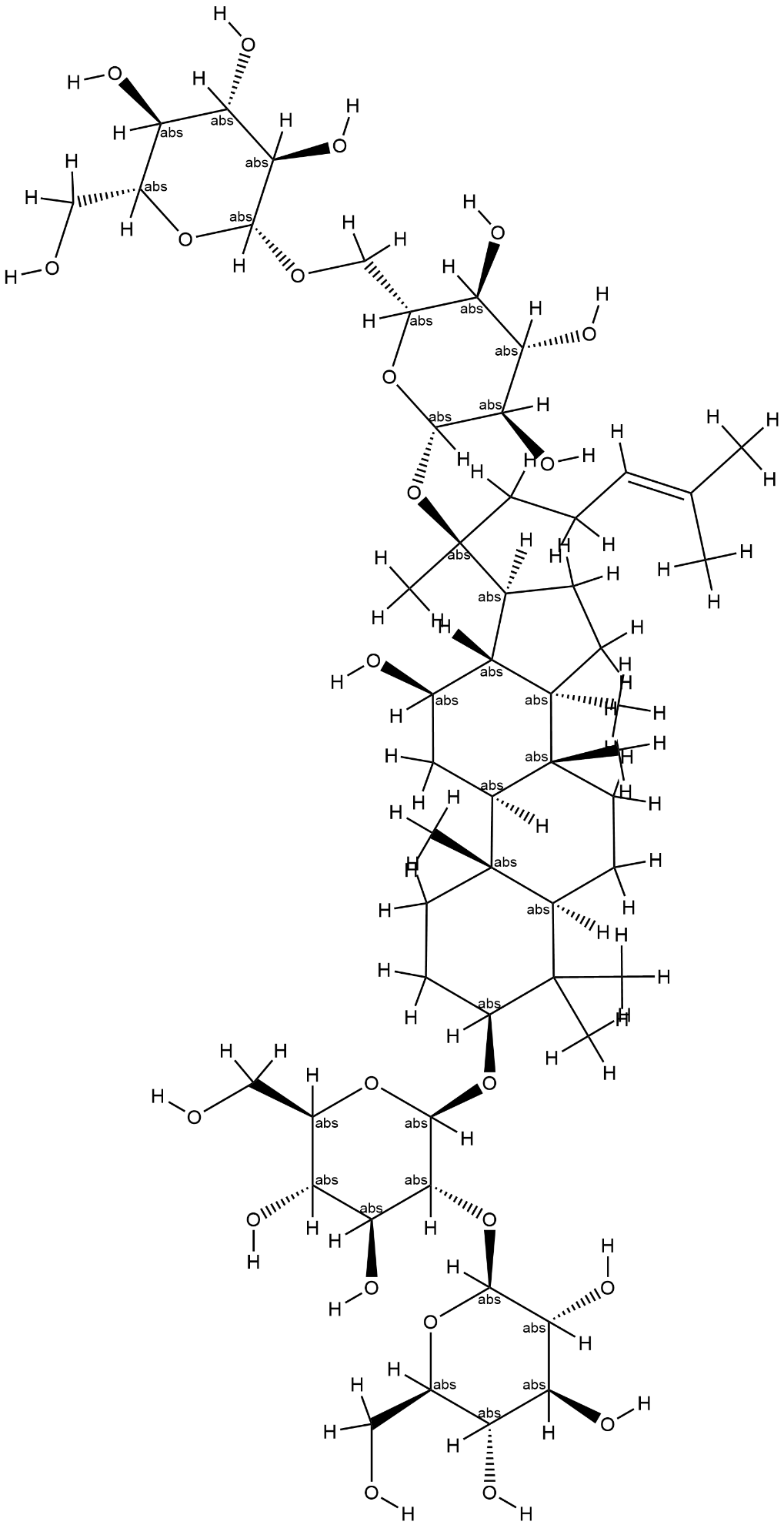


**Ginsenoside Re**


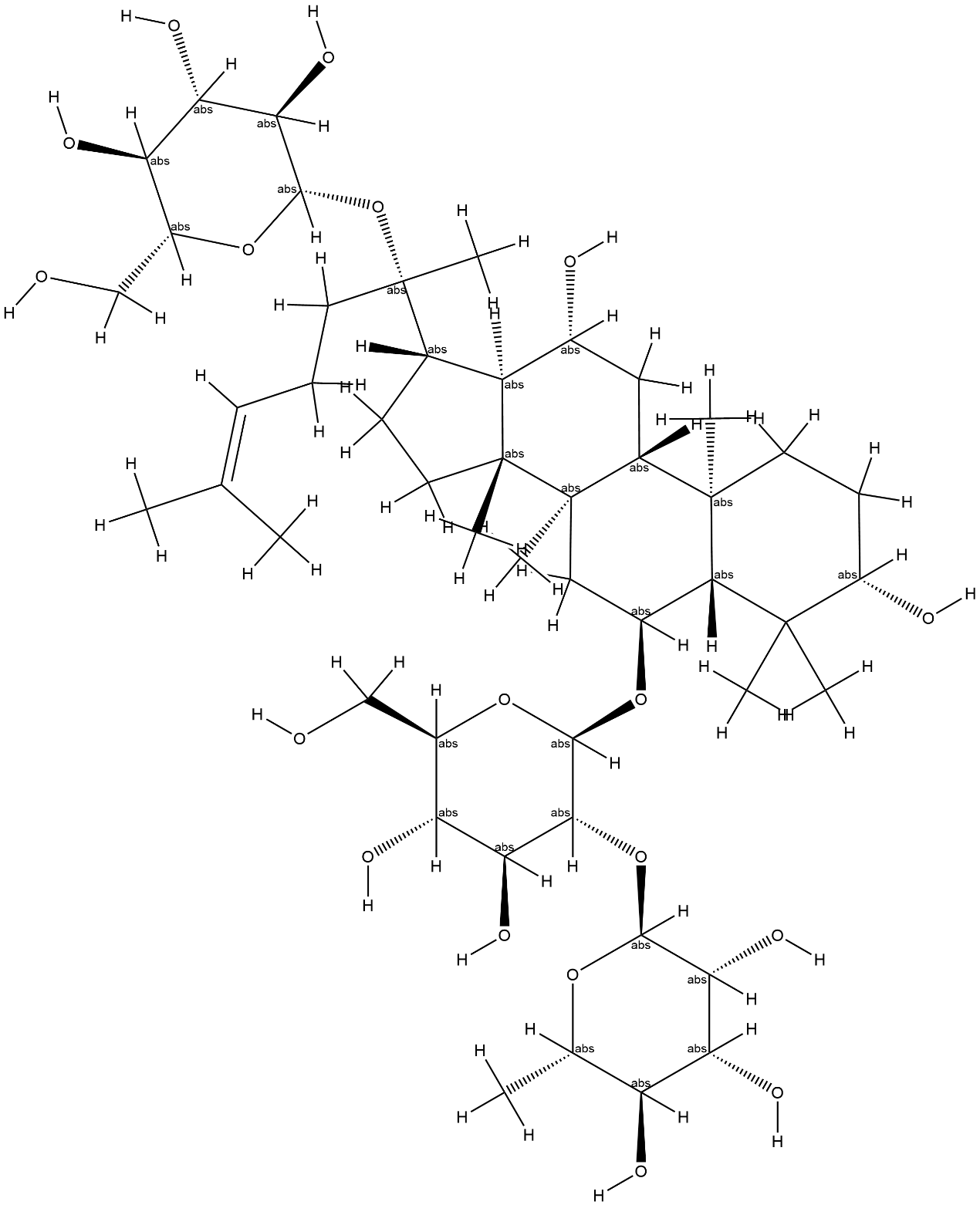


**Ginsenoside Rd**


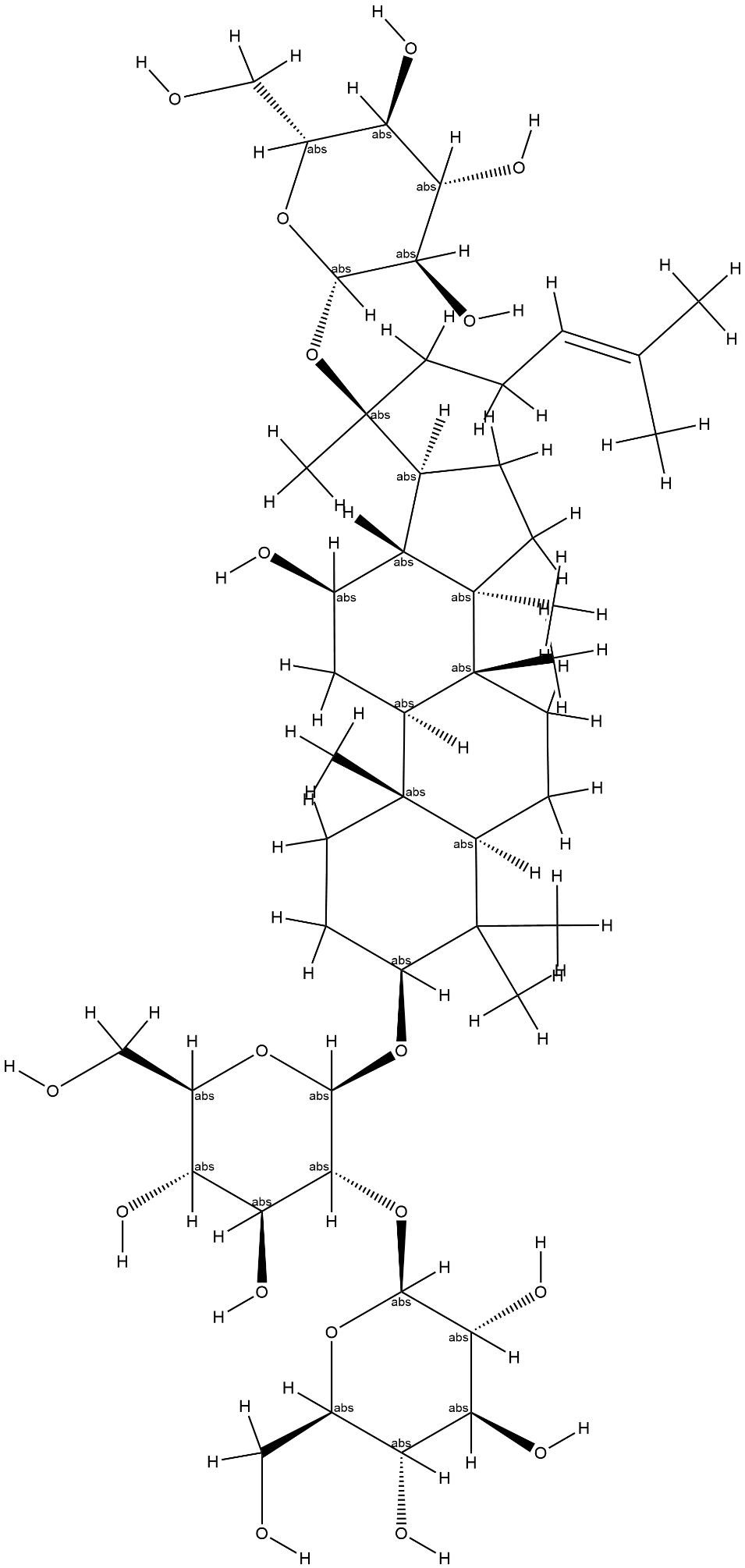


**Notoginsenoside R1**


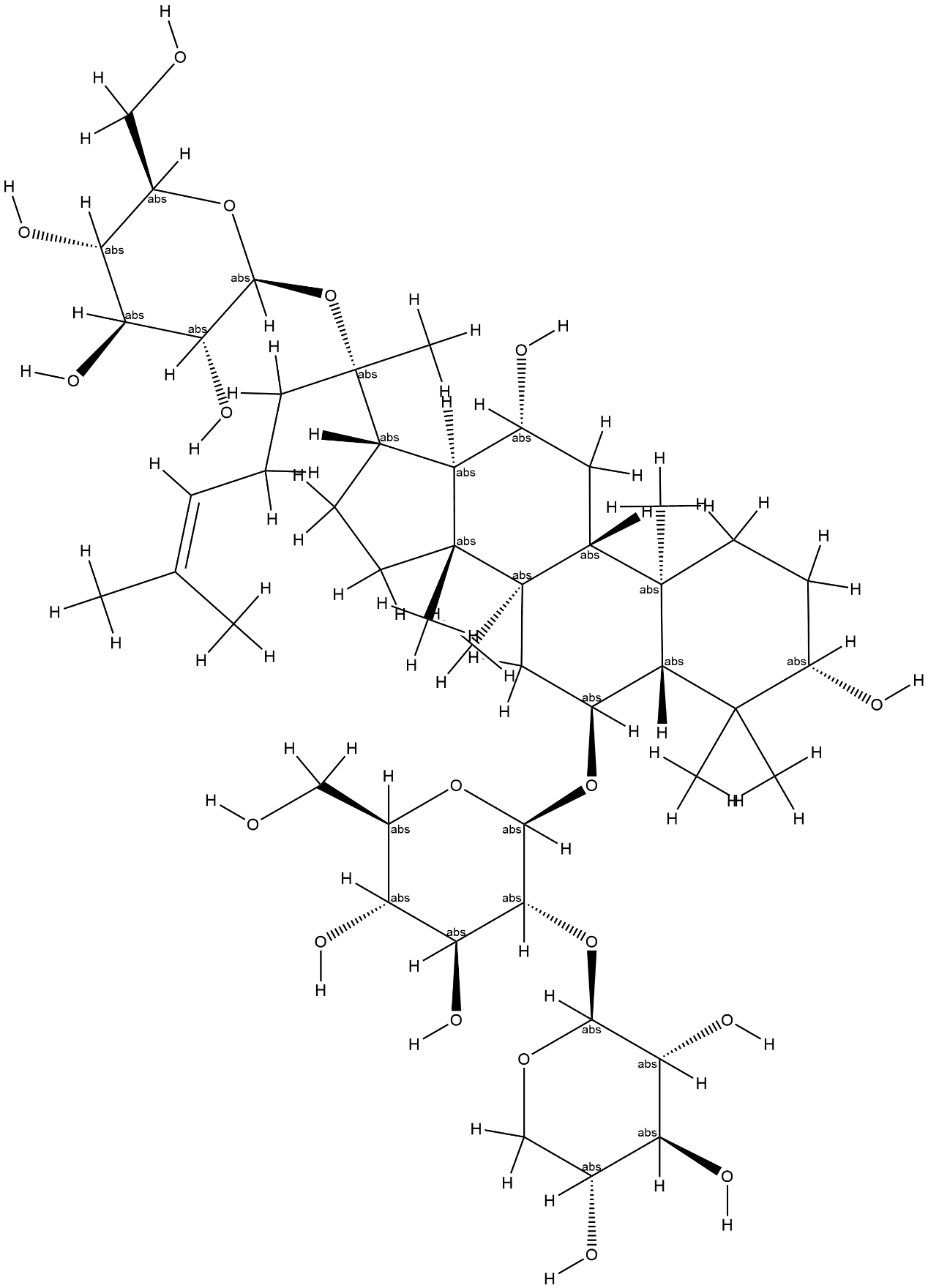

Supplement: Supplementary file 2 [file DataSheet2.docx]
