# Supplementary material for: Potential therapeutic effects of Chinese meteria medica in mitigating drug-induced acute kidney injury
Source: Front Pharmacol. 2023 Apr 3;14:1153297. doi: 10.3389/fphar.2023.1153297 (PMC10106589; doi:10.3389/fphar.2023.1153297)

**Ginsenoside Rb3**


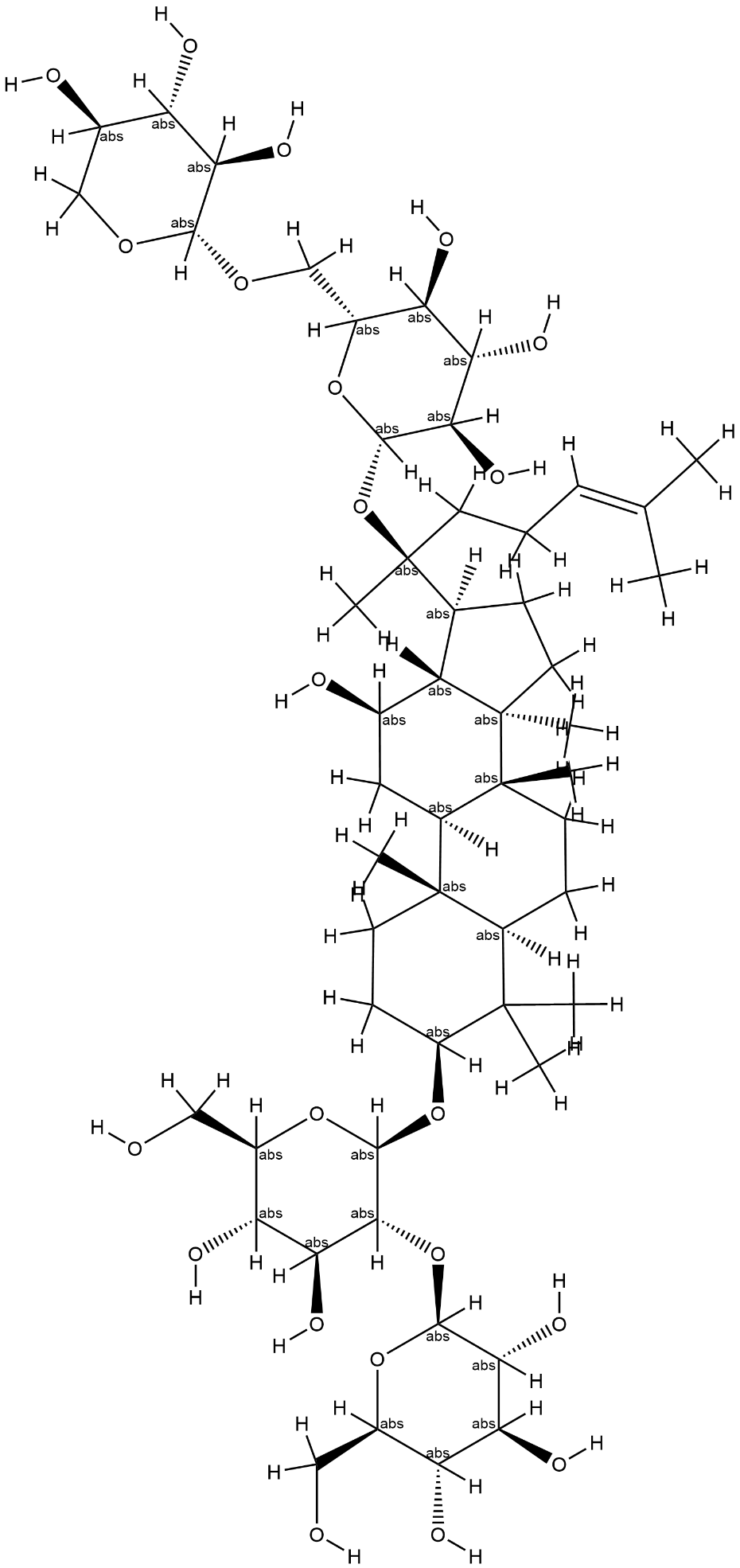


**Ginsenoside Rb**


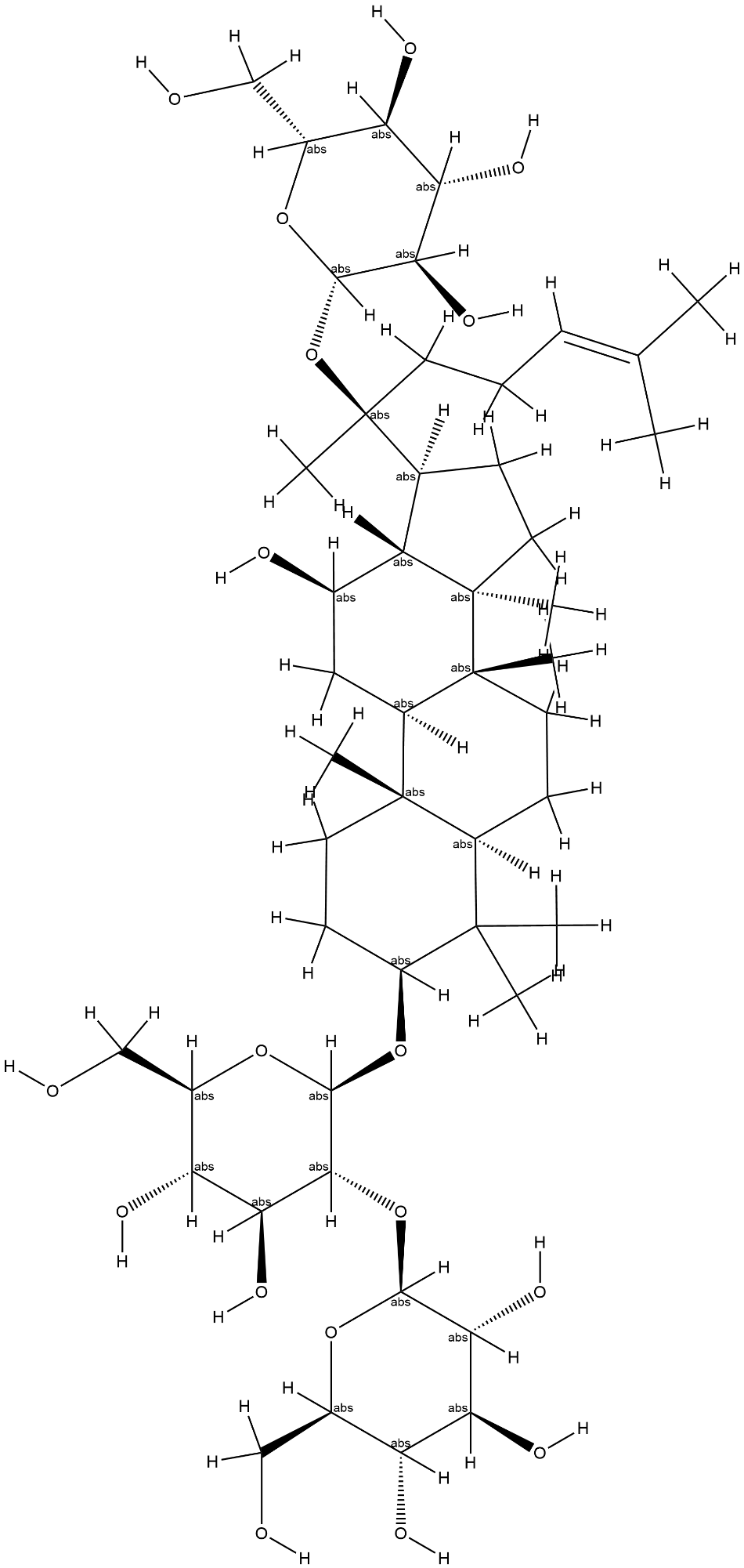


**Ginsenoside Re**


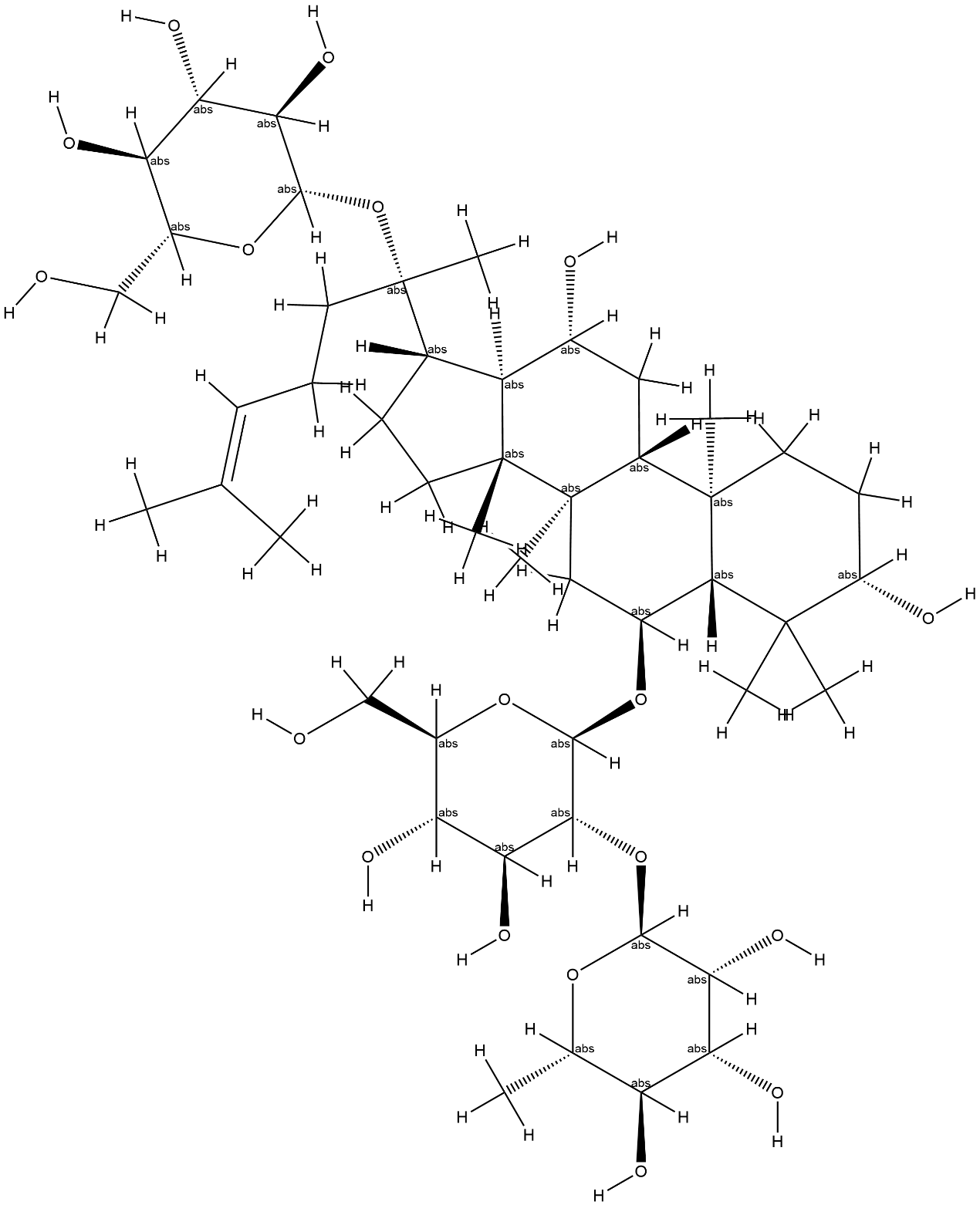


**Ginsenoside Rg3**


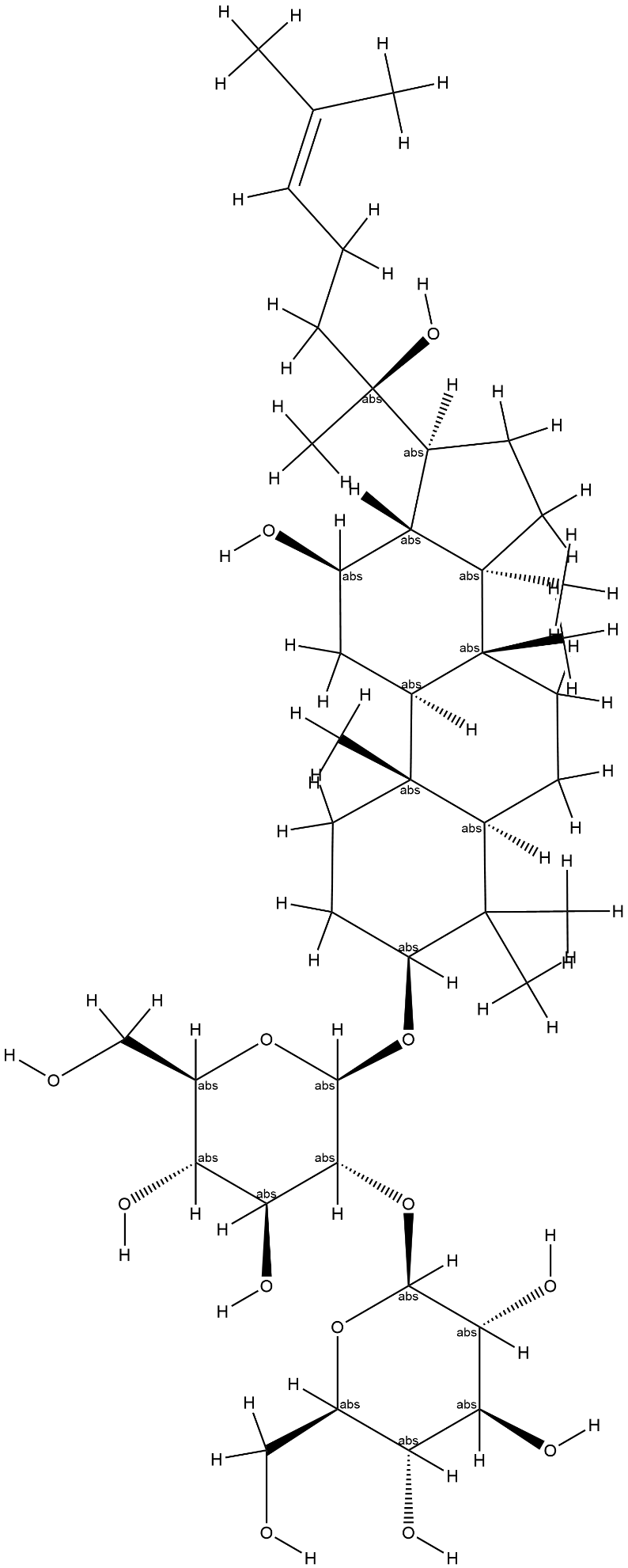


**Ginsenoside Rg5**


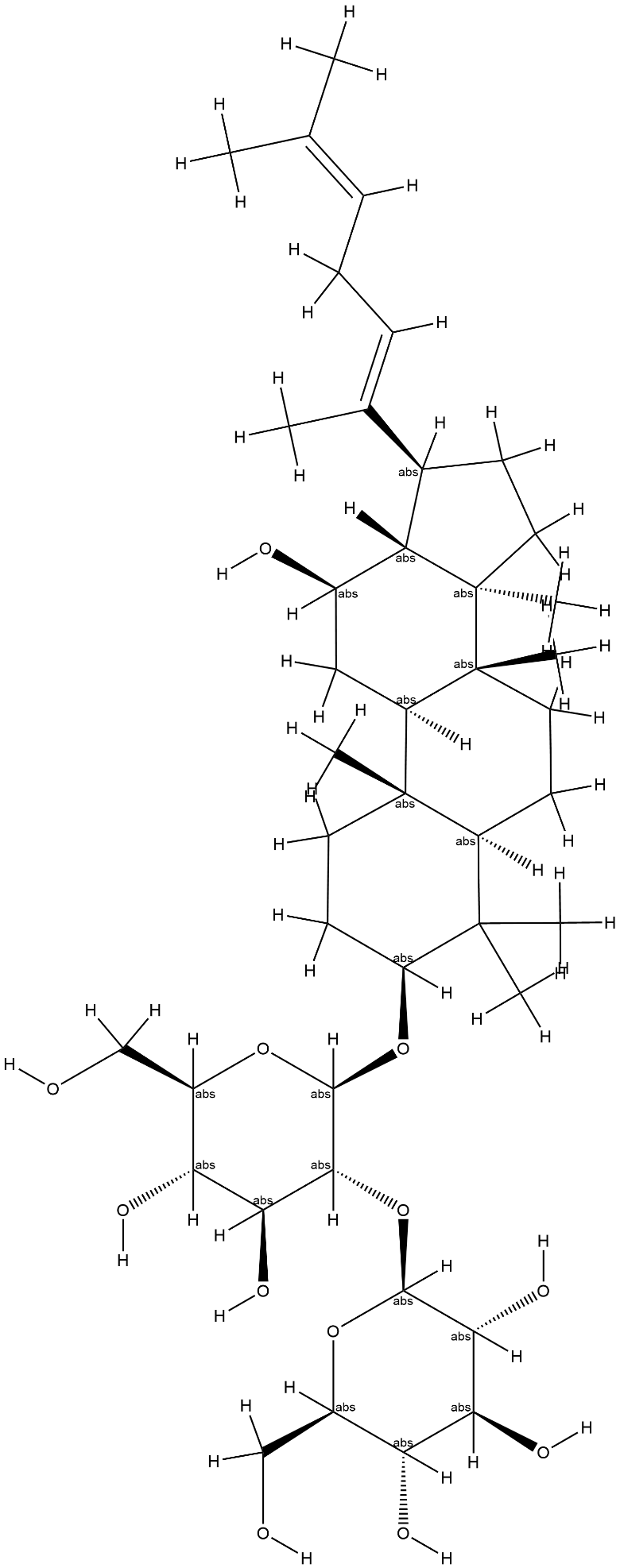


**Ginsenoside Rh2**


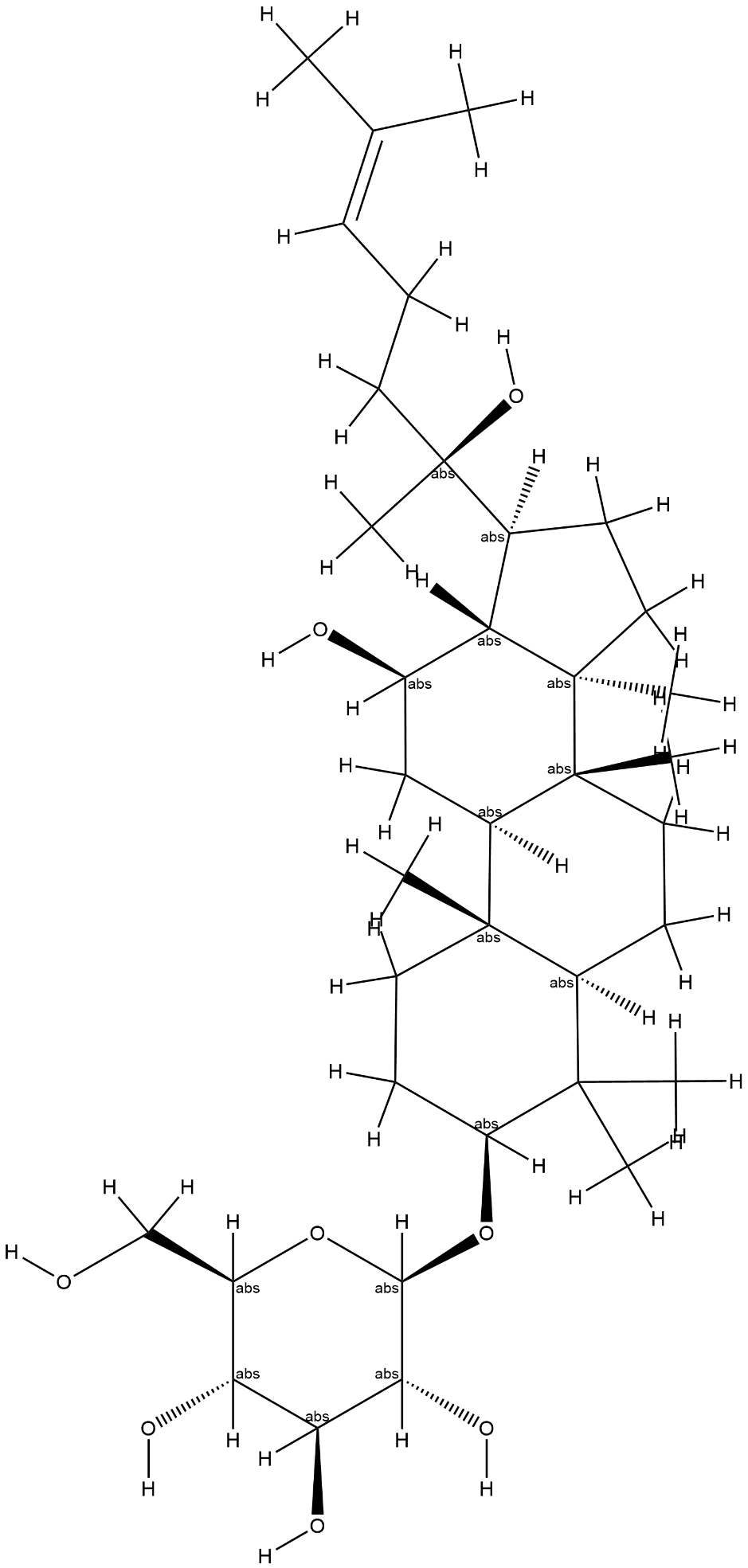


**Ginsenoside Rh3**


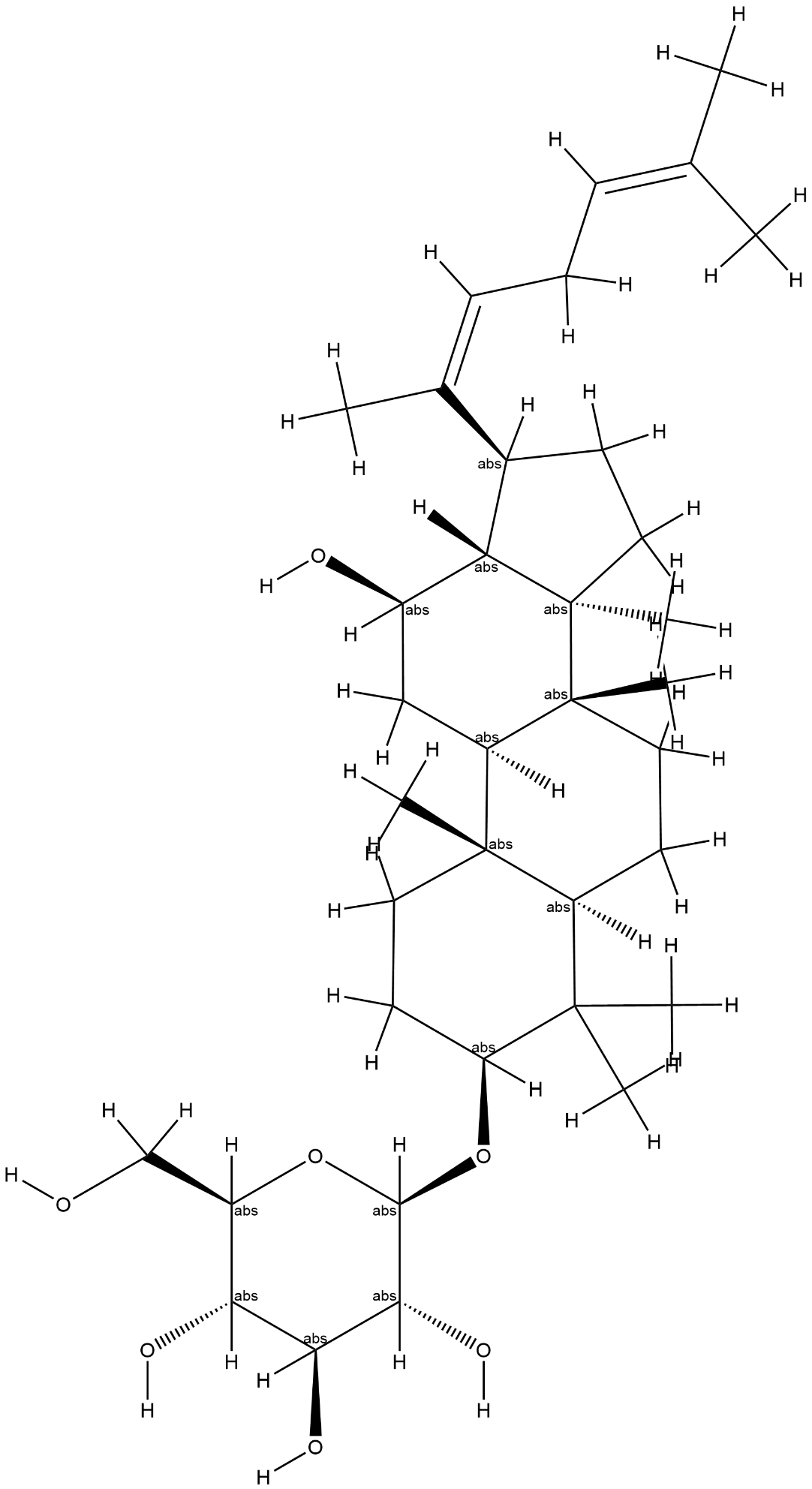


**Ginsenoside Rh4**


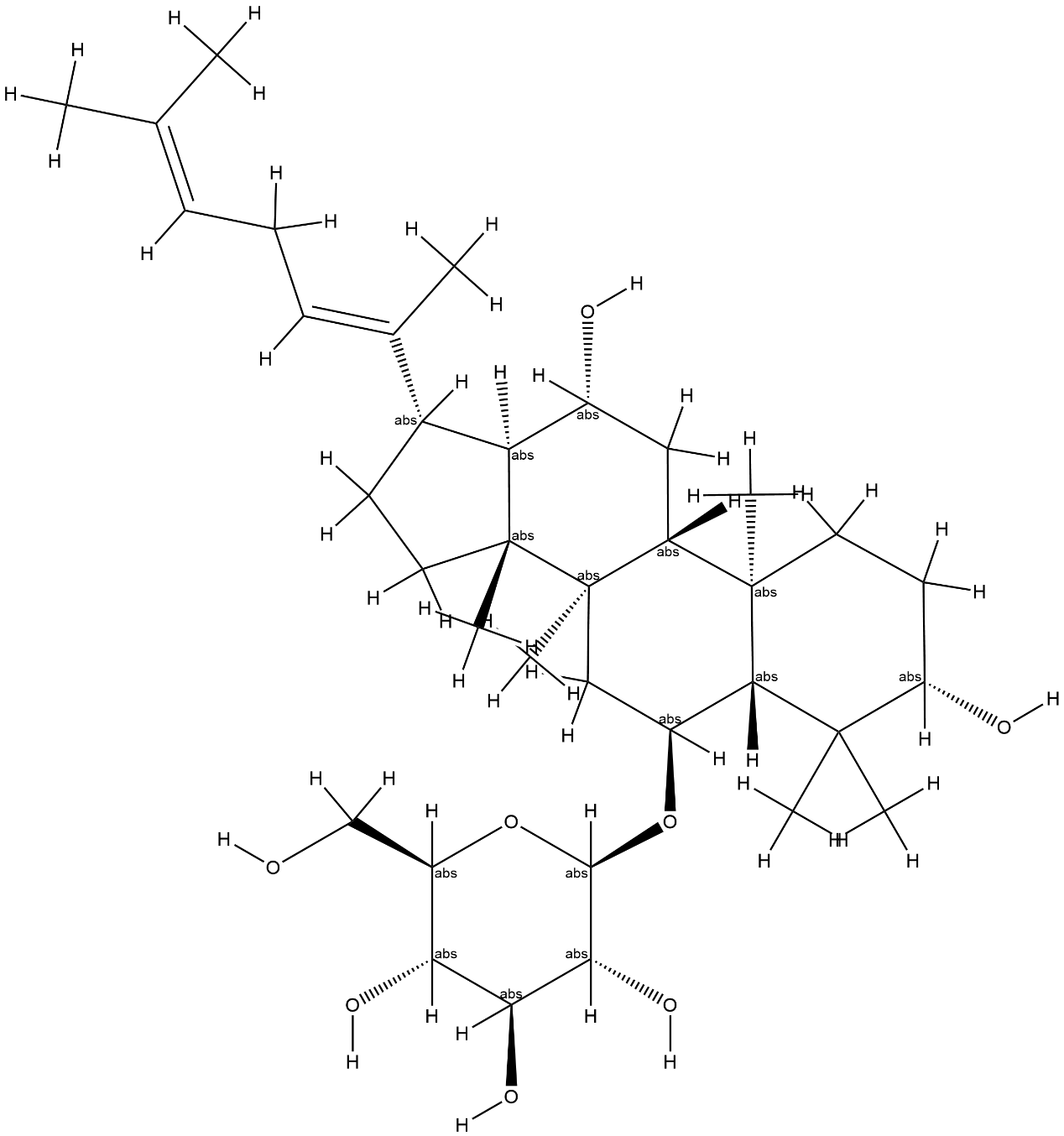


**Ginsenoside Rk1**


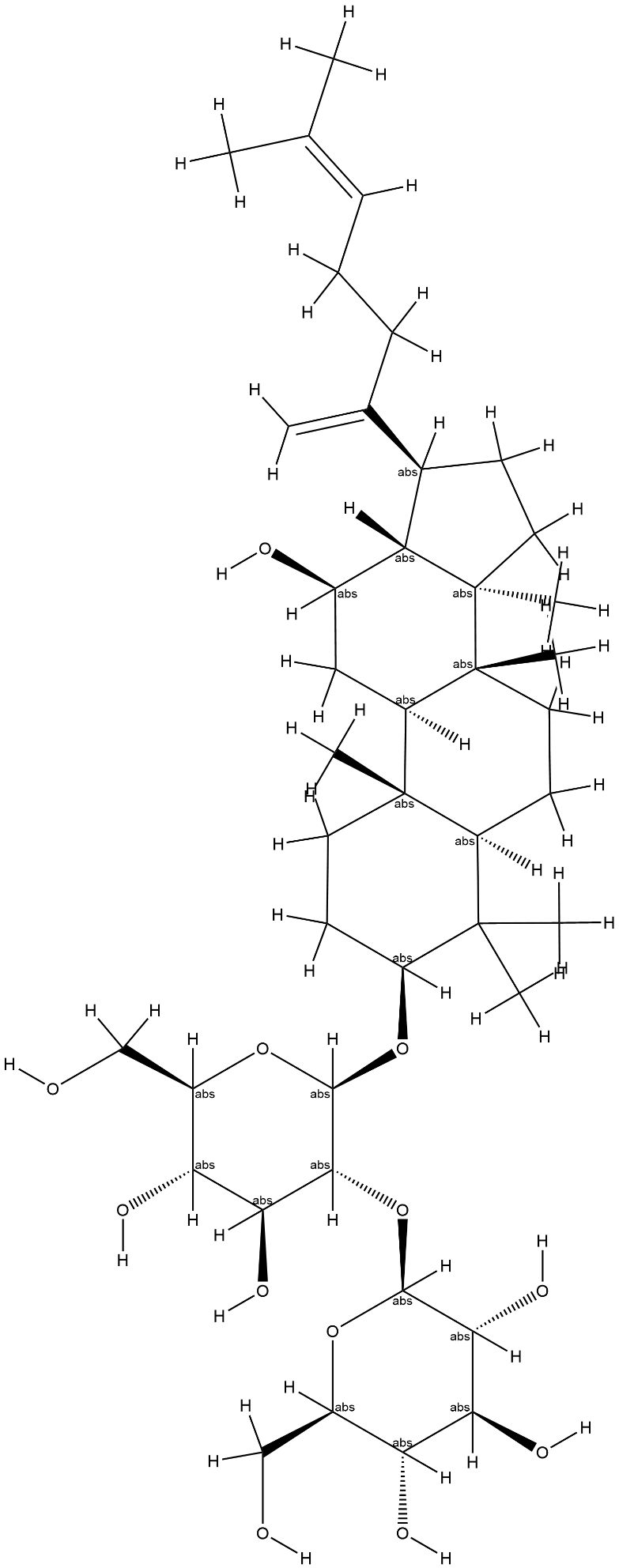


**Ginsenoside Rk3**


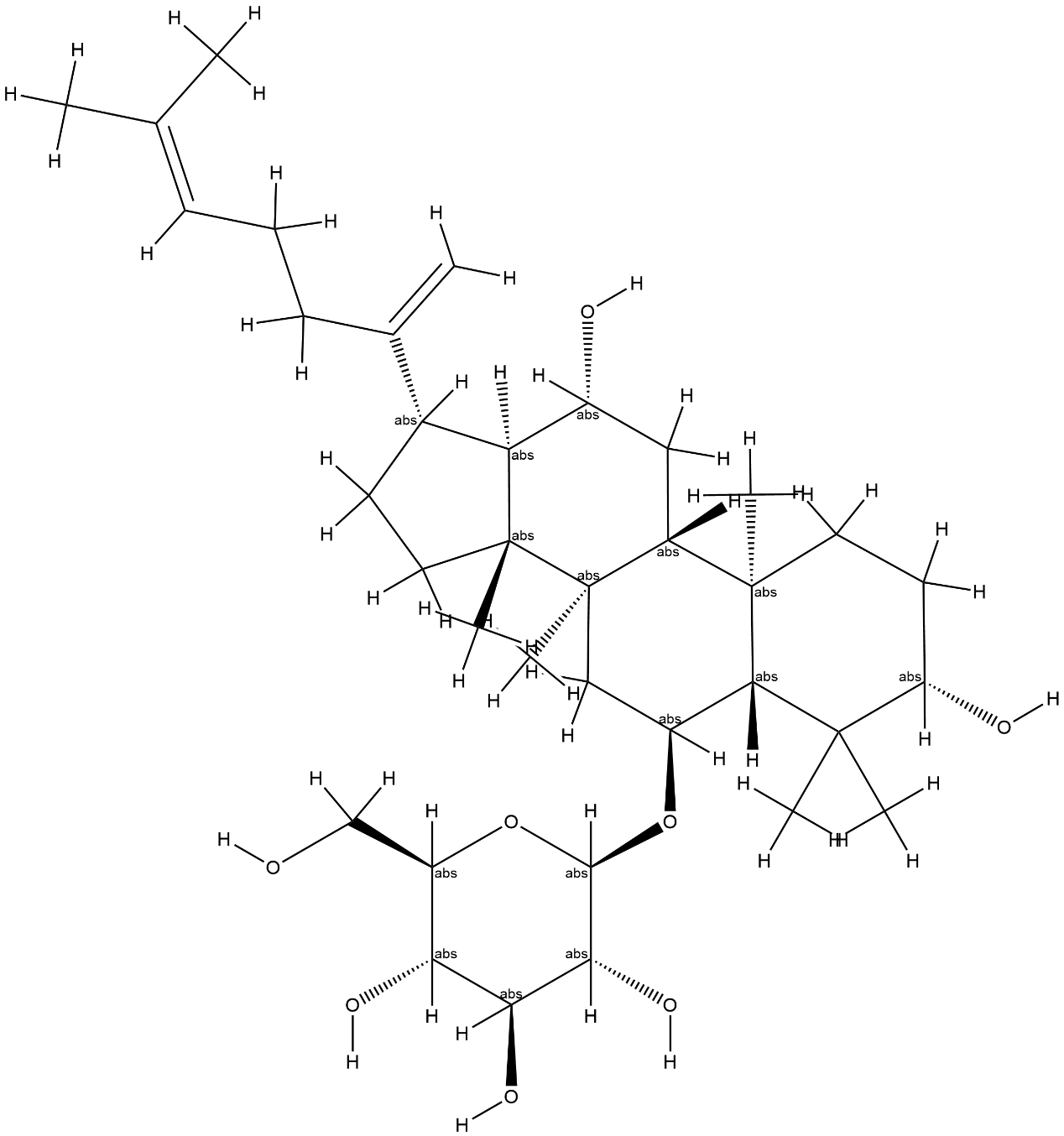


**Ginsenoside F11**


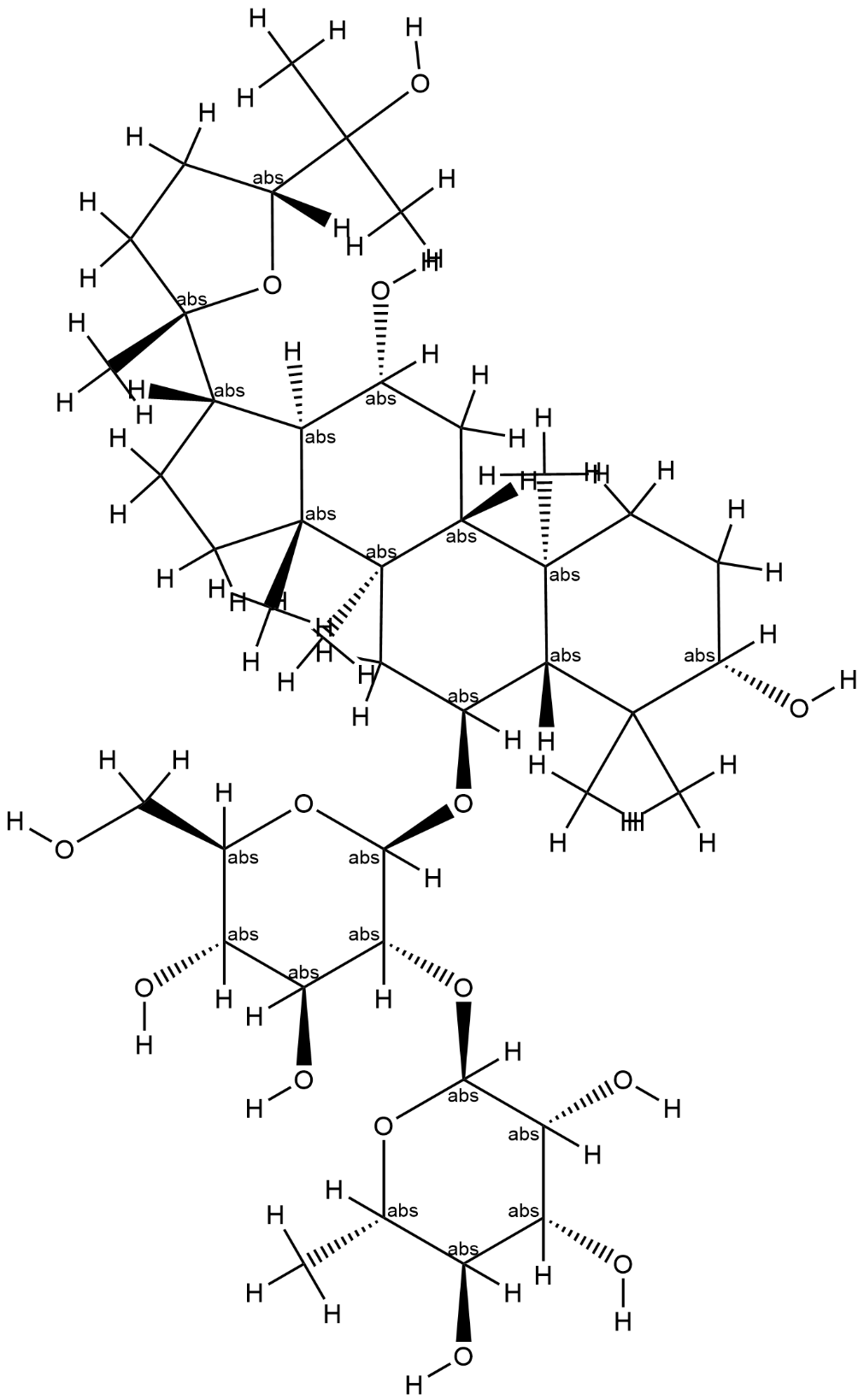

Supplement: Supplementary file 3 [file DataSheet1.docx]
